# Vaping during pregnancy and maternal, fetal, and infant health outcomes: A scoping review of human and animal studies

**DOI:** 10.1371/journal.pone.0356305

**Published:** 2026-09-29

**Authors:** Stella Fielder, Chloe Barnes, Laura Ormesher, Sarah Arrowsmith, Rehan Junejo, Michael Ussher, Alexander Heazell

**Affiliations:** 1 Maternal and Fetal Health Research Centre 5th floor (Research), Saint Mary’s Hospital, Manchester, United Kingdom; 2 Manchester University NHS Foundation Trust, Obstetrics, Manchester, England, United Kingdom; 3 Department of Life Sciences, Faculty of Science and Engineering, Manchester Metropolitan University, Manchester, England, United Kingdom; 4 Institute of Social Marketing and Health, University of Stirling, Stirling, United Kingdom; UCLA School of Dentistry, UNITED STATES OF AMERICA

## Abstract

**Background:**

The prevalence of vaping, defined as the use of e-cigarettes, during pregnancy is increasing, but its effects on maternal, fetal, and infant health remain incompletely understood. Human studies are heterogeneous and largely observational with a primary focus on neonatal and infant outcomes and limited assessment of maternal physiology. In contrast, pre-clinical animal studies suggest potential biological effects across maternal vascular, respiratory, metabolic, and neurodevelopmental systems. A comprehensive mapping of the existing evidence is therefore needed to characterise the scope of research to date and identify key gaps in the literature.

**Objective:**

To map the extent, range, and nature of human and animal studies examining the effects of vaping during pregnancy on maternal, fetal, and infant health outcomes, and to describe key methodological features and gaps to inform future research.

**Materials and methods:**

A scoping review was conducted following Joanna Briggs Institute methodology and PRISMA-ScR guidelines. Electronic database searches for human and animal studies examining the effects of vaping during pregnancy were conducted in MEDLINE, EMBASE, CINAHL, CENTRAL, PsycInfo, and grey literature, which were completed September 10th, 2025. Data were extracted on study design, population, exposure definitions, outcome domains, and use of biochemical exposure validation. No formal risk-of-bias assessment was undertaken, consistent with scoping review methodology, instead study characteristics were mapped descriptively.

**Results:**

64 studies were included (34 animal, 30 human). Human studies were predominantly observational and varied in design. Most relied on retrospective self-report without biochemical validation, potentially contributing to contradictory findings. Outcomes were focused mainly on perinatal measures, with maternal outcomes limited to breastfeeding, postpartum depression, and gestational weight gain, and little or no assessment of maternal physiological outcomes such as vascular or autonomic function. In contrast, animal studies frequently investigated mechanistic outcomes, suggesting potential biological effects across multiple systems including metabolic, respiratory, neurobehavioural, and cardiovascular impairment, with greater severity in nicotine-exposed groups. However, exposure paradigms and species differences limit direct translation to human pregnancy.

**Conclusion:**

The existing evidence base on vaping in pregnancy is characterised by substantial heterogeneity and notable gaps. In particular, there is a lack of prospective human studies incorporating objective exposure assessment and mechanistic maternal outcomes. Comparative analyses frequently evaluated vaping relative to combustible cigarette smoking. However, substantial variability in exposure classification, comparator groups, and adjustment for confounding limits interpretability. Animal studies more thoroughly examine mechanistic pathways and multiple organ systems. However physiological differences and unrealistic exposure models limit generalisability to human pregnancy. Overall, the current literature is insufficient to draw firm conclusions regarding risk. These findings highlight priorities for future research to strengthen the evidence base and improve understanding of the biological effects of vaping during pregnancy.

## Implications

This scoping review includes 34 animal and 30 human studies examining the effects of vaping during pregnancy. It demonstrates a rapidly expanding but methodologically diverse evidence base. By distinguishing exclusive vaping (EV), dual use, and combustible cigarette (CC) smoking this review highlights the substantial variability in exposure definitions, comparator groups, and outcome measurement across studies. Comparative analyses frequently evaluated vaping relative to CC smoking for outcomes such as low birth weight, preterm birth, and small-for-gestational-age. However, differences in exposure classification, adjustment for confounding, and reliance on retrospective self-reported data limit comparability and interpretability. Evidence comparing vaping with non-use was similarly inconsistent. This review also highlights mechanistic outcomes investigated by animal studies including vascular and endothelial dysfunction and the effects of flavouring constituents, areas that remain largely unexplored in human pregnancy research. Finally, this review identifies priorities for future research including prospective, biochemically validated studies that characterise device, nicotine, and flavourings, and assess maternal outcomes using standardised definitions.

## Introduction

Cigarette smoking in pregnancy is linked to low birth weight, preterm prelabour rupture of membranes, placental complications, stillbirth, fetal anomalies, and longer-term child risks including obesity, hypertension, asthma, and neurodevelopmental disorders such as attention deficit hyperactivity disorder [1–6]. Nicotine, present in cigarettes, many vapes, and nicotine replacement therapy (NRT), has been associated with adverse developmental effects in animal models on cardiovascular, neurological, and respiratory systems via placental oxidative stress [7–9]. Human data are limited, and animal studies often use nicotine-naïve models (i.e., animals with no prior nicotine exposure before experimental gestational exposure) and high doses, limiting generalisability. Evidence is unclear about the harm of NRT [10]. In some countries, NRT is recommended during pregnancy when the expected benefits of using NRT to achieve smoking cessation outweigh the risks of continued cigarette smoking [11,12].

In recent years, electronic-cigarette use, hereafter referred to as vaping (or exclusive vaping), has become popular and is commonly perceived as a safer alternative to smoking during pregnancy [13]. Vaping does not involve tobacco combustion, which is an important source of smoking harm. Vaping is increasingly used for smoking cessation and harm reduction in pregnancy, with US and UK data indicating a notable uptake [14,15].

Evidence from the general population suggests that vaping may be an effective smoking cessation tool [16]. One trial of vaping for smoking cessation in pregnancy suggested greater cessation with vaping than NRT in sensitivity analysis, with no short-term perinatal differences [17]. However, samples were small, there were no non-nicotine controls and follow-up ended at 3 months postpartum. While vaping avoids combustion, users may be exposed to nicotine and toxicants including volatile organic compounds (VOCs), polycyclic aromatic hydrocarbons, and heavy metals [18,19]. A separate rigorous pregnancy study found exclusive vapers had substantially lower selected VOCs and heavy metals than smokers, with levels comparable to never-users [20]. However, toxicants not assessed in this study, and exposures associated with higher intensity vaping remain poorly characterised.

Human studies assessing the associations between vaping during pregnancy and health outcomes have methodological limitations which affect the reliability of findings; most rely on retrospective, self-reported data without biochemical verification, leaving findings vulnerable to recall bias and confounding, particularly from previous and concurrent cigarette use. In addition, most human studies prioritise neonatal and infant outcomes, with limited assessment of maternal physiological outcomes. Two systematic reviews published in 2024 illustrate the methodological complexity of this evidence base [21,22]. Ussher et al. conducted a systematic review narrative synthesis of 26 studies and concluded that the evidence was inconclusive. They reported substantial variation in exposure definitions, comparator groups, and outcome measurement, and noted that inconsistent adjustment for smoking precluded quantitative pooling [21]. In contrast, Deprato et al. undertook a meta-analysis of 23 studies and generated pooled estimates, reporting 53% increased odds of both adverse maternal and neonatal outcomes associated with vaping during pregnancy. However, included studies varied considerably in exposure classification, comparator group (including non-use and combustible cigarette use), timing of exposure assessment, and adjustment for confounding. Statistical heterogeneity was moderate to high for several pooled analyses (e.g., I^2^ = 80% for maternal outcomes), reflecting variability across study designs and populations [22]. These differences in synthesis approach and underlying variability likely contributed to the contrasting interpretations [21,22]. Although these authors disagree about whether a meta-analysis was warranted, they agree on the limitations in exposure assessment and the urgent need for higher-quality research, particularly studies that incorporate biochemical verification and distinguish between EV, dual use, and non-use using strict criteria, to exclude any confounding effects of smoking [23,24].

In contrast to human data, animal studies frequently report growth, metabolic, respiratory, and neurological harms, implicating both nicotine and non-nicotine components and providing mechanistic insights largely absent from human pregnancy studies [25,26]. Given the increasing prevalence of vaping during pregnancy, rigorous prospective research with objective exposure assessment is urgently needed. This scoping review synthesises human and animal evidence to map outcome domains, characterise methodological features, and identify evidence gaps to inform future research and guidance. To our knowledge, no previous review has synthesised both human and animal studies to characterise translational gaps between mechanistic findings and clinical outcomes in pregnancy.

## Materials and methods

### Protocol and reporting framework

This scoping review was conducted in accordance with Joanna Briggs Institute (JBI) methodology and reported following the PRISMA-ScR guidelines [27,28]. The protocol was prospectively registered with INPLASY (registered 27 September 2024, Registration number: INPLASY202490123 [29]). The review was guided by the Population–Concept–Context (PCC) framework to define eligibility and scope.

### Eligibility Criteria (PCC Framework)

#### Population.

Eligible studies included pregnant humans and *in vivo* mammalian models of pregnancy (e.g., murine and rat models). Studies assessing health outcomes in offspring following antenatal exposure were eligible. *Ex vivo* tissue studies and non-mammalian models were excluded. Studies examining postnatal-only exposure were also excluded.

#### Concept.

The core concept was antenatal vape exposure (nicotine-containing or nicotine-free) including both active and passive aerosol exposure in animal models. Studies examining exclusive vaping (EV), dual use (vaping plus combustible cigarette use), combustible cigarette comparison, nicotine-free exposure, and passive exposure were eligible. Studies evaluating exposure to specific device components (e.g., flavouring constituents) within *in vivo* mammalian models were also included.

#### Outcomes.

Eligible outcomes included maternal, fetal, neonatal, and infant health outcomes.

***Outcome domains included:***

- Pregnancy complications (e.g., preterm birth, fetal growth restriction)
- Anthropometric measures (e.g., birth weight, fetal and placental weight, biometric indices)
- Maternal physiological endpoints (e.g., autonomic function, endothelial function)
- Neonatal and infant outcomes (e.g., breastfeeding, neonatal mortality, NICU admission)
- Organ system outcomes (respiratory, metabolic, neurological, cardiovascular, renal, immune, neurobehavioural)

#### Context.

No geographic restrictions were applied. Studies were included if published in English or where an English translation was available.

### Study design

Primary research studies were eligible, including randomised controlled trials, cohort studies, case–control studies, cross-sectional studies, other observational designs, and experimental mammalian animal studies. Case reports were eligible where relevant to predefined outcome domains. Systematic reviews and meta-analyses were screened to identify additional primary studies but were not included in the synthesis.

### Information sources and search strategy

Electronic searches were conducted in MEDLINE, EMBASE, CINAHL, CENTRAL, and PsycInfo from database inception to 10 September 2025. Grey literature was searched using Google Scholar, with the first 100 results screened using predefined search terms. This was undertaken as a pragmatic supplementary search, informed by JBI guidance recommending review of the first 5–10 pages of results from supplementary Google searches [30]. Searches were conducted in incognito mode to minimise algorithmic personalisation. Search strategies combined controlled vocabulary and free-text terms relating to e-cigarettes/vaping and pregnancy. The search strategies were developed by the review team and included both human and mammalian *in vivo* animal studies, reflecting the broader scope of this review compared with previous systematic reviews that focused exclusively on human outcomes. Full database-specific search strategies are provided in S1 Appendix.

### Study selection

All records were imported into Covidence for duplicate removal and screening. Titles and abstracts were independently screened by two reviewers (SF and CB). Full-text articles of potentially eligible studies were independently assessed. Disagreements were resolved through discussion or consultation with a third reviewer (AH).

### Data extraction

Data were extracted using a standardised data collection form developed prior to review commencement.

For human studies, extracted variables included:

- Study design, country, and setting
- Sample size and population characteristics
- Exposure definition (EV, dual use, combustible cigarette use, non-use)
- Comparator group
- Timing of exposure assessment
- Biochemical exposure validation (e.g., cotinine, anabasine, carbon monoxide (CO) monitoring)
- Adjustment for confounding variables
- Outcome domains assessed
- Effect measures reported (e.g., odds ratio (OR), relative risk (RR))

For animal studies, extracted variables included:

- Species and strain (mammalian models only)
- Exposure modality (e.g., aerosol inhalation, injection)
- Nicotine concentration and device characteristics where reported
- Flavourings and additives
- Timing and duration of exposure (pre-gestational, gestational, lactational)
- Comparator groups (nicotine-containing vs nicotine-free vs control)
- Outcome domains assessed, including cardiovascular, metabolic, respiratory, neurological, immune, behavioural, anthropometric, and placental measures
- Measurement modality for physiological endpoints (e.g., pulse wave velocity, endothelial function testing)

Methodological characteristics were systematically extracted, including exposure classification, comparator definition, timing of exposure, and use of biochemical validation, to enable structured characterisation of differences across studies. The full extracted dataset, including study characteristics and outcomes, is provided in S2 Appendix.

### Data synthesis

Data were synthesised narratively and organised by outcome domain to enable structured comparison across studies. Human and animal studies were summarised separately to characterise methodological variability, exposure definitions, comparator structures, and translational gaps between mechanistic and clinical findings. In keeping with scoping review methodology, no formal risk-of-bias assessment was undertaken. Rather than grading study quality, the review aimed to map study characteristics and identify evidence gaps. Detailed study level data are presented in S2 Appendix.

Although formal risk-of-bias assessment is not routinely performed in scoping reviews, we undertook a structured interpretability appraisal of the included human studies to support transparent synthesis [28]. The appraisal considered study design and data source, prospective versus retrospective exposure ascertainment, timing and frequency of exposure assessment, definitions of EV, combustible cigarette smoking, dual use and non-use, biochemical validation, previous smoking and switching behaviour, sample size, particularly the number of exclusive vaping participants, confounder adjustment, and potential residual confounding from previous or concurrent combustible cigarette use. Studies were then categorised descriptively as higher, moderate, moderate-to-lower, or lower interpretability. Higher interpretability reflected stronger features across these domains, including prospective or repeated exposure assessment, biochemical validation, clearly defined exposure groups, appropriate consideration of previous smoking and dual use, larger exposed groups, and more comprehensive adjustment for relevant confounders. Interpretability was reduced where several limitations were present, particularly retrospective self-reported exposure, absence of biochemical validation, small vaping groups, incomplete characterisation of previous smoking or dual use, limited confounder adjustment, or substantial residual confounding. Categories were assigned holistically across these domains rather than using a weighted numerical score. This appraisal was not intended to constitute a formal risk-of-bias or quality assessment, but to map the methodological features most relevant to interpreting associations between vaping and pregnancy outcomes. The structured appraisal is presented in S4 Appendix.

### Ethics

Ethical approval was not required because this scoping review synthesised data from previously published studies and did not involve direct recruitment of participants or collection of identifiable individual-level data

## Results

### Description of the included studies

Fig 1 (PRISMA) summarises study identification and selection. We included 34 animal (mouse/rat) and 30 human studies. Animal outcomes spanned anthropometric (n = 18), metabolic (n = 5), respiratory (n = 11), neurodevelopmental/behavioural (n = 10), cardiovascular (n = 8), and other (n = 5) domains, often with multiple outcomes per study and both nicotine and non-nicotine exposures. Human studies most commonly assessed low birth weight (LBW) (n = 16), preterm birth (PTB) (n = 15), and small for gestational age (SGA) (n = 15), with smaller numbers addressing other maternal, fetal and neonatal outcomes.

**Fig 1:**
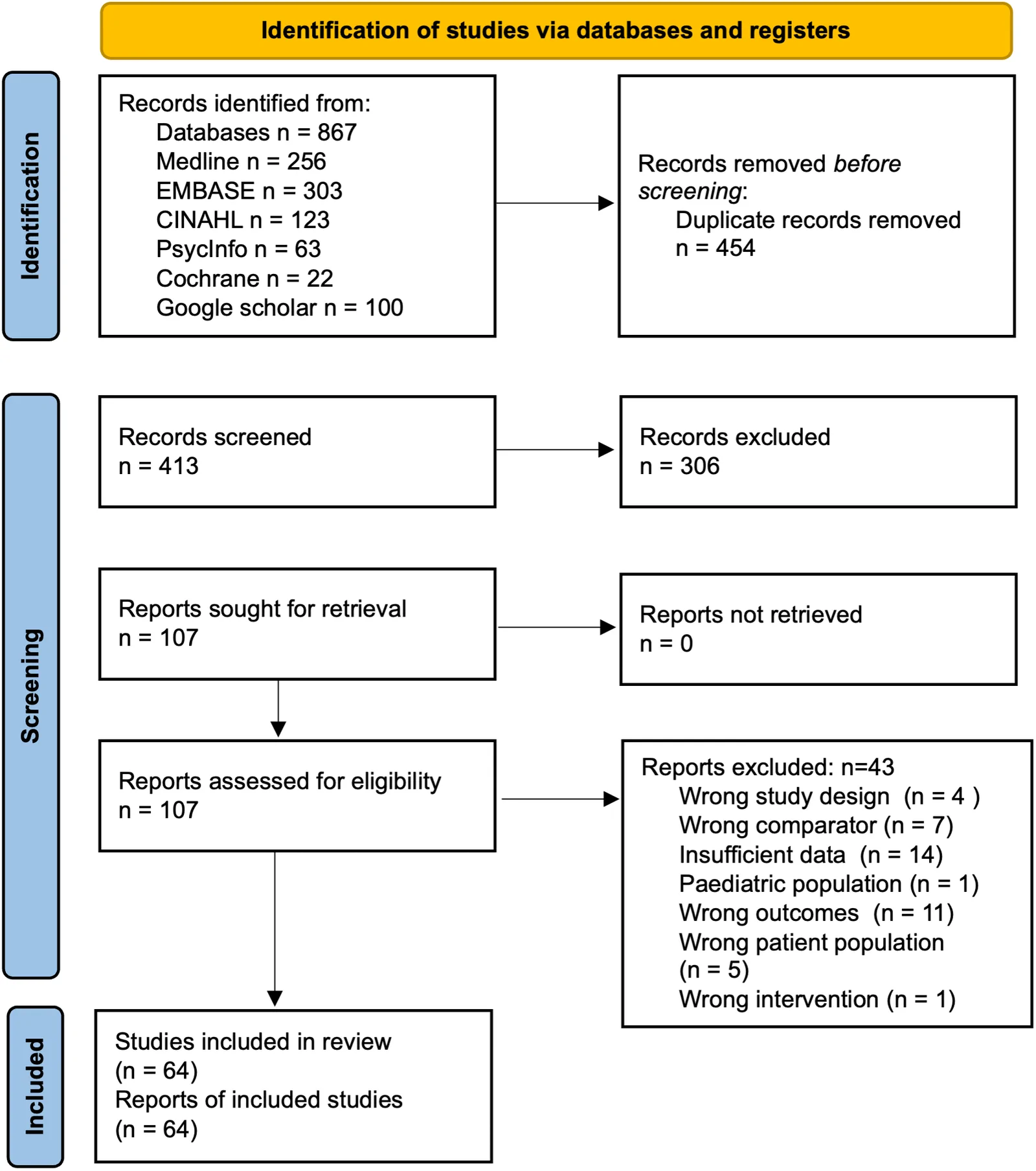
Preferred reporting items for systematic reviews and meta‐analyses (PRISMA) flow diagram.

A structured interpretability appraisal of the human studies is provided in S4 Appendix. This summarises exposure timing, definitions of EV, dual use and non-use, biochemical validation, previous smoking and dual use, residual confounding from prior smoking, confounder adjustment, key interpretability limitations and overall interpretability rating.

### Evidence mapping of human and animal outcomes

Fig 2 maps the distribution of human and animal evidence across outcome domains and highlights areas of thematic overlap and translational uncertainty. Human evidence was concentrated on low birth weight, preterm birth and small for gestational age, whereas animal studies more extensively examined respiratory, neurodevelopmental, cardiovascular, endothelial and metabolic outcomes.

**Fig 2:**
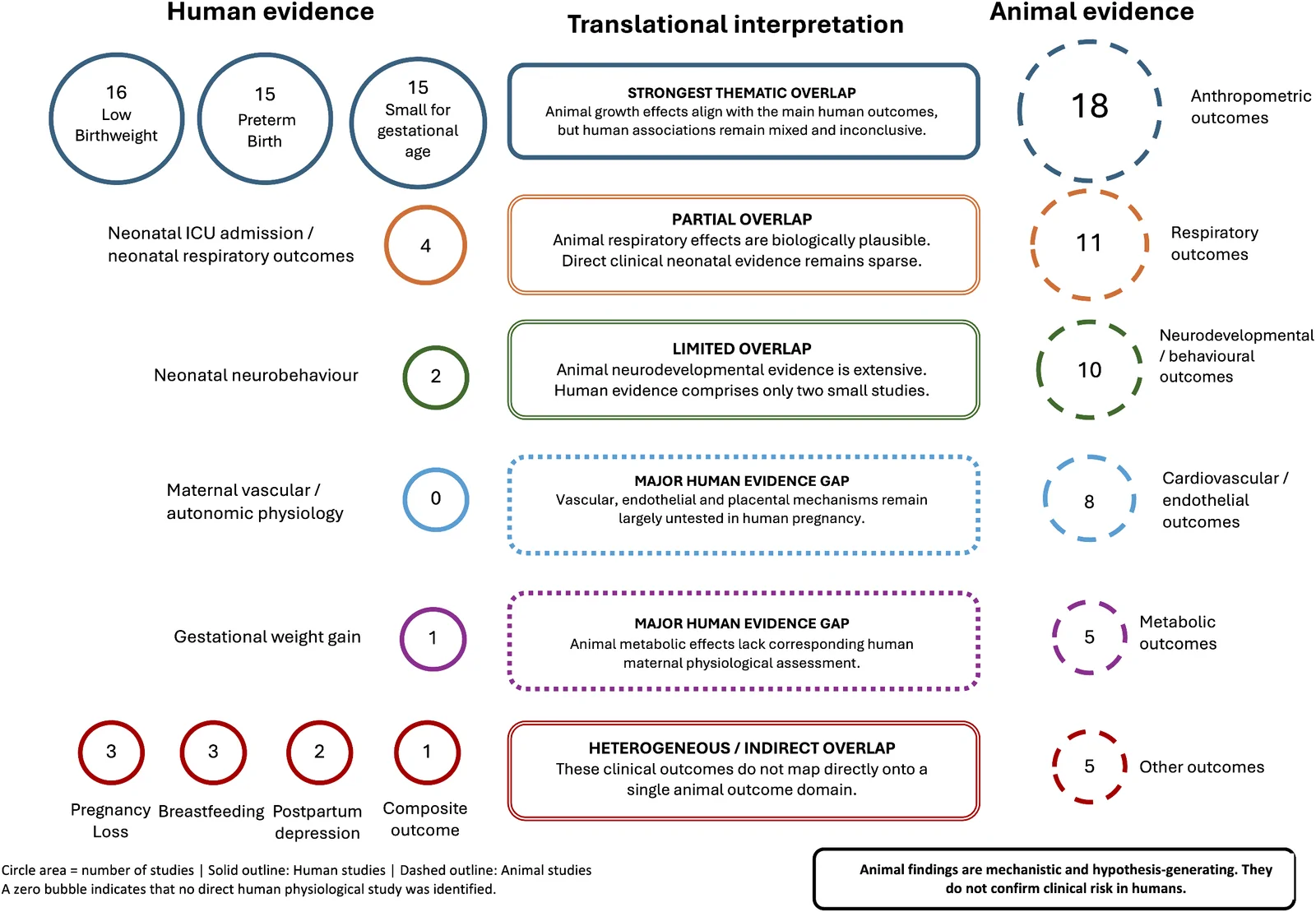
Evidence map comparing human and animal studies of vaping during pregnancy. Bubble size represents the number of studies assessing each outcome domain. Solid circles show human evidence and dashed circles show animal evidence. The central panels highlight areas of overlap and key translational gaps.

### Animal studies

A summary of study findings is presented in Table 1.

**Table 1:** Results of animal studies.

Outcome	Exclusive vaping (EV) with nicotine vs non-use			Exclusive vaping (EV) without nicotine vs non-use		
	Increased Risk	Reduced Risk	No Difference	Increased Risk	Reduced Risk	No Difference
**Low birth weight**	Archie (2023) [31] Cahill & Johnson (2022) [32] Cahill & Gartia (2022) [33] Kishinchand (2022) [34] Mills (2023) [35] Noel (2020) [36] Orzabal (2019) [37] Orzabal (2021) [38] Orzabal (2022) [39] Reynolds (2025) [40] Sifat (2020) [41] Walayat (2021) [42]		Church (2020) [43]	Mills (2023) [35] Richlak (2025) [44]		Church (2020) [43] Orzabal (2019) [37]
**Cardiovascular and endothelial dysfunction**	Aboaziza (2023) [45] Burrage (2020) [46] Burrage (2021) [47] Mills & Corbin (2025) [48] Orzabal (2019) [37] Mills & Dakhallah (2025) [49] Nassabeh (2023) [50] Reynolds (2025) [40]			Aboaziza (2023) [45] Burrage (2020) [46] Burrage (2021) [47] Mills & Corbin (2025) [48] Mills & Dakhallah (2025) [49]		
**Metabolic dysfunction**:	Aboaziza (2023) [45] AlHarthi (2023) [51] Aslander (2022) [52] Li (2020) [53] Mills (2023) [35] Mills & Corbin (2025) [48]	Chen & Chapman (2018) [54] Chen & Nguyen (2018) [55]	Burrage (2021) [47]	Aboaziza (2023) [45] AlHarthi (2023) [51] Aslander (2022) [52] Chen & Chapman (2018) [54] Chen & Nguyen (2018) [55] Li (2020) [53] Mills (2023) [35] Mills & Corbin (2025) [48]		Burrage (2021) [47] Yu (2024) [56]
**Hematological dysfunction**	Alarabi (2024) [57] Yu (2024) [56]					
**Neurobehavioural dysfunction**	AlHarthi (2023) [51] Archie (2023) [31] Church (2020) [43] Mills & Corbin (2025) [48] Nguyen (2018) [58]			AlHarthi (2023) [51] Church (2020) [43] Mills & Corbin (2025) [48] Nguyen (2018) [58]		
**Neurological dysfunction**	Al-Saewalha (2020) [59] Archie (2023) [31] Awada (2024) [60] Church (2020) [43] Mills & Corbin (2025) [48] Sifat (2020) [41] Walayat (2021) [42]			Awada (2024) [60] Church (2020) [43] Mills & Corbin (2025) [48]		
**Respiratory dysfunction**	Aslander (2022) [52] Cahill & Johnson (2022) [32] Cahill & Gartia (2022) [33] Chen & Chapman (2018) [54] McAlinden (2017) [61] Noel (2020) [36] Noel (2023) [62] Orzabal (2021) [38] Orzabal (2022) [39] Ozekin (2023) [63] Wang & Sundar (2020) [64]			Aslander (2022) [52] Chen & Chapman (2018) [54] Orzabal (2021) [38] Wang & Sundar (2020) [64]		McAlinden (2017) [61] Orzabal (2022) [39] Ozekin (2023) [63]
**Skeletal abnormality**	Kishinchand (2022) [34] Ozekin (2023) [63]			Kishinchand (2022) [34] Richlak (2025) [44]		Ozekin (2023) [63]
**Reduced litter size**	Mills & Corbin (2025) [48] Mills & Dakhallah (2025) [49]		Church (2020) [43] Orzabal (2019) [37]	Mills & Corbin (2025) [48] Mills & Dakhallah (2025) [49]		Church (2020) [43] Orzabal (2019) [37]
**Reduced maternal gestational weight gain**	Archie (2023) [31]		Orzabal (2022) [39]			Orzabal (2022) [39]

#### Anthropometric data.

Eighteen studies examined anthropometric data; primarily birthweight, weanling weight, and crown–rump length. Nicotine-containing vapour exposure was consistently associated with growth restriction, only Church et al. reported no significant difference in fetal weight [43]. Orzabal et al. demonstrated 25–47% fetal weight reduction and 12–24% reduction in crown–rump length, relative to controls [37–39]. Ten other studies noted similar restrictions in growth [31–34,36,40–42,54]. All but one associated this with nicotine-containing vapes [44]. Findings from nicotine-free vapour exposure were inconsistent. One study identified a 16–18% weight reduction at postnatal day (PND) 21, with similar effects seen in another study [35,65]. Conversely, Aslander et al. identified a higher body weight in females and Chen et al. found a higher PND 20 weanling weight [52,54]. Two studies found no difference between the non-nicotine vaping cohorts and controls [37,43]. In summary, most studies reported reduced fetal or postnatal growth following exposure to nicotine-containing vapour. Associations with non-nicotine vapes remain inconclusive.

Three studies reported skeletal abnormalities, including shorter long bones and impaired craniofacial development, less pronounced without nicotine [34,44,63]. Notably, one study found that the ratio of propylene glycol (PG) to vegetable glycerin (VG) influenced outcomes, with skeletal abnormalities observed at a 30PG/70VG ratio but not at 50PG/50VG [44]. Orzabal et al. identified maternal amino acid dysregulation, especially in growth and nitrogen metabolism pathways, which were more pronounced in nicotine exposed dams [38].

#### Metabolic health.

Five studies identified systemic and hepatic metabolic dysregulation [38,45,48,51,53]. Aboaziza et al. reported increased adiposity and reduced lean mass after both nicotine and non-nicotine exposure, persisting into adulthood in the nicotine-free group, suggesting a predisposition to obesity [45]. Two studies corroborated these findings, identifying increased weight gain during adolescence and higher body mass at 6 and 12 months [48,51]. Li et al. demonstrated that both nicotine-containing and nicotine-free vapour exposure impaired hepatic metabolic function in dams and pups [53]. Dams from both groups exhibited increased expression of glucose transporter GLUT4 and an increased HOMA-IR index, an indicator of insulin resistance [53]. The nicotine-free group also demonstrated elevated liver triglycerides and plasma non-esterified fatty acids (NEFA), suggesting increased lipolysis [53,66]. In addition, nicotine-exposed pups had increased expression of FOXO1, a hepatic gluconeogenesis marker, and elevated liver triglycerides suggesting increased lipogenesis [53,67]. Nicotine-free pups had increased mRNA expression of glucose transporters GLUT2 and GLUT4 and elevated FOXO1. Orzabal et al. noted increased levels of branched-chain amino acids in the nicotine-exposed group, which is frequently linked to insulin resistance and amino acid dysregulation [68]. These findings suggest potential long-term metabolic and epigenetic changes following in utero exposure to nicotine and non-nicotine containing vapour which may predispose to both obesity and insulin resistance.

#### Respiratory outcomes.

Eleven studies reported adverse effects on respiratory development, with more severe outcomes with nicotine exposure [32,33,36,38,39,52,54,61–64]. Across several studies, lung structural and histological alterations were observed, with features indicative of fibrotic remodelling, emphysematous changes, and allergic/asthmatic phenotypes. Fibrosis, characterised by increased lung stiffness, reduced compliance, tissue deposition, and a lower lung tissue-to-airspace ratio, was described in five studies [33,36,52,63,64]. Orzabal et al. noted an emphysema-like phenotype [39], two additional studies supported these findings, observing decreased lung fibrillar collagen [32,52]. Two described asthma-like features, including eosinophilia, neutrophilic inflammation, and elevated airway resistance, less pronounced in nicotine-free offspring [36,61].

Immune dysregulation and inflammation were noted. Nicotine mediated upregulation of inflammatory markers, with greater predisposition to allergic inflammation and eosinophilia, was reported in two studies [32,61]. Chen et al. found altered respiratory cytokine profiles across nicotine and nicotine-free groups. Dams exhibited elevated TNF-α, IL-1β, and IL-6, indicating heightened pulmonary inflammation, while pups had reduced IL-1β, suggesting a dysregulated postnatal immune response [54]. Lung-specific genetic dysregulation was also reported, again more marked with nicotine-containing vapour. Orzabal et al. identified 3,306 dysregulated genes in fetal lung tissue [39], supported by Ozekin et al. and Noel et al., who reported 2,563 and 75 dysregulated genes respectively [36,63]. Affected biological pathways included mitochondrial dysfunction, protein degradation, metabolic regulation, DNA damage response, lung development, and inflammatory signalling [63]. Together, these studies report structural, immunological and epigenetic alterations in pulmonary development, with generally greater effects from nicotine-containing vapes when compared to an unexposed control group.

#### Neurological, developmental, and behavioural outcomes.

Ten studies investigated neurodevelopmental and behavioural outcomes following antenatal vape exposure, broadly categorised into cognitive, neurochemical, metabolic and epigenetic impacts. Several studies reported cognitive dysfunction, characterised by impaired memory and increased hyperactivity, in offspring irrespective of nicotine exposure [31,41,43,48,51,58,59]. All nicotine-containing studies also reported reduced anxiety-like behaviours, whereas nicotine-free findings were mixed [58,65].

Oxidative stress emerged as a recurrent mechanism. Al-Sawalha et al. reported elevated hippocampal superoxide dismutase levels, indicating compensation for oxidative damage [59]. While Walyat et al. found elevated levels of reactive oxygen species were associated with greater infarct sizes following hypoxic ischaemic injury (HII) [42]. Sifat et al. reported similar findings with nicotine-exposed offspring demonstrating vulnerability to HII, with larger infarcts, delayed reflex recovery and impaired neuronal variability when compared to an unexposed control group [41]. Church et al. noted reduced neuronal anti-inflammatory cytokines in nicotine and non-nicotine groups, suggesting impairment mediated by multiple interacting pathways [43]. Awada et al. identified increased hypothalamic expression of glucose metabolism proteins (GLUT-1, AMPAR, PPARγ) in both nicotine and non-nicotine groups and Sifat et al. observed increased neuronal glucose uptake and ATP production, suggesting altered cerebral energy metabolism [41,60]. Mills & Corbin reported altered expression of clock genes (Arnt1, Per1, Per3, Clock, Cry1, Cry2), which regulate circadian rhythms, alongside evidence of neuronal damage (silver staining), increased astrocyte activation and astrocyte–endothelial interactions, and reduced SIRT1 with elevated NOX1, Aβ1–40, and Aβ1–42 in both nicotine and non-nicotine groups. Importantly, these changes persisted to 12 months, suggesting ongoing neuroinflammation and oxidative stress that may underlie chronic neurological dysfunction [48,49].

A diverse range of epigenetic modifications were also observed. In nicotine-exposed pups, Archie et al. reported reduced expression of proteins critical for blood–brain barrier integrity (ZO-1, claudin-5, occludin), astrocyte function (GFAP), glucose transport (GLUT-1) and water homeostasis (AQP4) [31,69]. Decreased cerebral glucose transporter expression was also noted by Sifat et al. [41]. Neurodevelopmental disruption was not confined to nicotine groups. Awada et al. identified upregulation of neuroinflammatory genes (PPARγ, LepRb, MC4R, AMPK, POMC, SLC2A1) in both nicotine and non-nicotine-exposed pups [60]. Similarly, Nguyen et al. reported increased global DNA methylation and downregulation of epigenetic regulators (DNMT1, DNMT3A, HDAC1, MECP) in carrier-aerosol exposed pups [58]. These findings suggest that both nicotine-containing and nicotine-free vapour exposure disrupts neurodevelopment through overlapping cognitive, oxidative, metabolic, and epigenetic pathways.

#### Cardiovascular outcomes.

Eight studies assessed the effect of antenatal exposure to both nicotine and non-nicotine-containing vapes on cardiovascular development and function when compared to an unexposed control group [35,40,45–50]. Both nicotine-containing and nicotine-free exposures were associated with vascular alterations across studies, with oxidative stress repeatedly implicated as a key mechanism.

Two studies identified increased arterial stiffness, evidenced by elevated pulse wave velocity (PWV), with Aboaziza et al. noting persistence into adulthood [45,50]. Cerebrovascular dysfunction was observed by Burrage et al. and Mills et al. who reported reduced endothelial-dependent dilation of the middle cerebral artery (MCA) [35,46–49]. Partial restoration of vascular function with oxidative stress modulators further implicates this pathway [46,48,49]. Histological assessment revealed altered aortic structure; nicotine exposure reduced elastin and elastin-to-collagen ratio, while non-nicotine exposure was associated with increased collagen content and impaired reactivity [45]. Reynolds et al. reported increased maternal cardiac arrhythmias, accelerated ventricular repolarization, and elevated cardiac output in nicotine-containing vape–exposed groups, with raised 3-methoxytyramine levels suggesting a mechanism of sympathetic dominance [40]. These findings suggest that antenatal vape exposure, with or without nicotine, may be associated with long-term cardiovascular dysregulation in animal models, with oxidative stress implicated as a potential mechanism.

#### Other outcomes.

Five studies examined effects beyond major organ systems. Alarabi et al. identified disrupted haemostasis, in nicotine-exposed pups, with shortened bleeding/occlusion times and altered platelet gene and miRNA expression [57]. Yu et al. noted impaired bone marrow stem cell function with reduced proliferation, migration, and wound healing capacity, and molecular changes suggesting a fibrotic shift but only in nicotine-exposed pups [56].

Two studies reported increased pup resorption and smaller litter sizes, irrespective of nicotine content [48,49]. Orzabal et al. provided a potential mechanistic explanation, demonstrating marked reductions in maternal uterine (−49.5%) and fetal umbilical (−65.3%) artery blood flow following nicotine exposure, consistent with impaired placental perfusion as a driver of growth restriction and fetal loss, although this was only seen in the nicotine-exposed group [37]. Overall, these studies report alterations in haematological, immune, and placental function, particularly with nicotine-containing vapour.

### Methodological characteristics of included animal studies

Across animal studies, exposure patterns and formulations varied substantially in nicotine concentration, vehicle composition (propylene glycol/vegetable glycerin ratios), flavouring additives, and aerosol delivery systems. Timing of exposure differed across studies, including combined pre-gestational/gestational [57], gestational [32–36,38–45,47–52,54,59,60,62–64], and combined pre-gestational/gestational/lactational models [31,37,53,55,56,58,61]. Dose standardisation and quantification of aerosol exposure were inconsistently reported. Many studies used nicotine-naïve dams and relatively high exposure concentrations, which may limit direct translational inference to human pregnancy. Outcome measures ranged from molecular and epigenetic markers to structural and physiological endpoints, with varying follow-up durations into adolescence or adulthood. These methodological differences complicate direct comparison across models and should be considered when interpreting mechanistic findings, especially when trying to extrapolate mechanistic pathways to human physiology.

### Human studies

A summary of study findings is presented in Table 2.

**Table 2:** Summary of key outcomes and study findings.

Outcome	Exclusive vaping (EV) vs non-use			Exclusive vaping vs combustible cigarette (CC) use and/or dual use		
	Increased Risk	Reduced Risk	No association	Increased Risk	Reduced Risk	No Difference
**Low birth weight**	Ammar (2023) [70] Azar (2024) [71] Bailey (2024) [72] Cui (2022) [73] Galbo (2022) [74] Kim (2020) [75] Nian (2025) [76] Regan & Bombard (2021) [77] Regan & Pereira (2021) [78]		Ashford (2021) [79] Froggatt (2020) [80] Hawkins (2022) [81] Lin (2023) [82] McDonnell (2020) [83] Opondo (2021) [84] Pesola (2024) [85]	Regan & Bombard (2021) [77] (Dual)	Ammar (2023) [70] Azar (2024) [71] Cui (2022) [73] Froggatt (2020) [80] Hawkins (2022) [81] Lin (2023) [82] McDonnell (2020) [83] Nian (2025) [76] Pesola (2024) [85]	Ashford (2021) [79] Kim (2020) [75] Regan & Pereira (2021) [78]
**Preterm birth**	Ammar (2023) [70] Galbo (2022) [74] Kim (2020) [75] Nian (2025) [76] Regan & Bombard (2021) [77] Regan & Pereira (2021) [78]	Pesola (2024) [85]	Cui (2022) [73] Hawkins (2022) [81] Lin (2023) [82] Nanninga (2023) [86] Opondo (2021) [84] Wang (2020) [87] Wang (2022) [88] Wen & Moe (2024) [89]	Ammar (2023) [70] (dual) Regan & Bombard (2021) [77] (dual)	Cui (2022) [73] Hawkins (2022) [81] (CC) Lin (2023) [82] Nian (2025) [76] Wang (2020) [87] Wang (2022) [88]	Ammar (2023) [70] (CC) Kim (2020) [75] Nanninga (2023) [86] Pesola (2024) [85] Regan & Pereira (2021) [78]
**Small for gestational age**	Bailey (2024) [72] Cardenas (2019) [90] Cardenas (2020) [91] Cui (2022) [73] Kim (2020) [75] Nanninga (2023) [86] Nian (2025) [76] Regan & Pereira (2021) [78] Shittu (2022) [92] Wang (2020) [87]		Ammar (2023) [70] Hawkins (2022) [81] McDonnell (2020) [83] Opondo (2021) [84] Regan & Bombard (2021) [77] Wang (2022) [88] Wen & Liu (2023) [93]	Ammar (2023) [70] Nanninga (2023) [86] Wang (2020) [87]	Cui (2022) [73] Hawkins (2022) [81] McDonnell (2020) [83] Nian (2025) [76] Shittu (2022) [92] Wang (2022) [88]	Kim (2020) [75] Regan & Bombard (2021) [77] (dual) Regan & Pereira (2021) [78] Wen & Liu (2023) [93]
**Neonatal respiratory distress/antibiotics**		Azar (2024) [71]			Azar (2024) [71]	Ashford (2021) [79]
**NICU admission/transfer**			Azar (2024) [71] Pesola (2024) [85]		Azar (2024) [71]	Ashford (2021) [79] Pesola (2024) [85]
**Need for neonatal antibiotics**						
**Postpartum depression**	Wen & Kwak (2024) [94]		Choi (2024) [95]			Wen & Kwak (2024) [94]
**Neonatal neurobehavioural disruption**	Froggatt (2020) [80]		Froggatt (2021) [96]		Froggatt (2020) [80] Froggatt (2021) [96]	
**Miscarriage/ pregnancy loss**	Nanninga (2023) [86]		Le (2025) [97] Lin (2023) [82]	Nanninga (2023) [86]		Le (2025) [97]
**Breastfeeding problems**	McBride (2021) [98] McDonnell (2020) [83]				McDonnell (2020) [83]	
**Adverse maternal outcome**: including HTN, GDM, PPH and respiratory symptoms			Nanninga (2023) [86]		Pesola (2024) [85]	Nanninga (2023) [86]
**Low maternal gestational weight gain**			Wen & Thomas (2023) [99]		Wen & Thomas (2023) [99]	

#### Low birth weight.

Sixteen studies examined vaping and LBW, with mixed findings. Only two studies employed biochemical validation [79,85], the others relied on self-report. Nine studies reported an increased risk of LBW among exclusive vapers versus non-users. Six were large Pregnancy Risk Assessment Monitoring System (PRAMS) analyses. Reported adjusted risk ratios (aRR) for LBW included 1.38 (95% CI 1.09–1.75), while adjusted odds ratios (aOR) ranged from 1.53 (95% CI 1.06–2.22) to 1.68 (95% CI 1.15–2.47), an adjusted prevalence ratio (aPR) of 1.33 (95% CI 1.06–1.66) was reported for LBW [70,73,75–77]. One study additionally reported a lower prevalence of LBW among individuals who quit vaping during pregnancy compared with ongoing vapers (aPR 0.53, 95% CI 0.47–0.60) [78]. Galbo et al. reported higher odds of a composite adverse birth outcome, which included LBW, identifying a 62% increase among exclusive vapers (aOR 1.62, 95% CI 1.16–2.26) [74]. However, repeated reliance on a single surveillance dataset (PRAMS), which is based on retrospective self-report, may limit external replication across independent cohorts. Bailey et al. found a significantly lower birth weight, length and head circumference in the EV cohort compared with non-users [72]. In a large US retrospective cohort study spanning 70 hospitals (n = 29,103), Azar et al. found exclusive vapers had higher odds of LBW compared with non-users (aOR 1.6 [95% CI 1.03–2.61]) [71].

In contrast, seven studies identified no association between LBW and EV [79–85]. These were derived from independent prospective cohorts and non-PRAMS datasets [80–84], and two included biochemical validation [79,85]. A secondary analysis of PRAMS data found no association between vaping and birthweight (−57.8g [95% CI −134.3, 18.6]) and two small UK studies (EV n = 10 and n = 27) also supported this, but conclusions were limited by sample size [80,81,84]. McDonnell et al. (EV n = 218) in a prospective cohort study including 218 exclusive vapers, observed no difference in birth weight (BW EV 3470 ± 555 g vs BW non-use 3471 ± 504 g p = 0.97) [83], consistent with Lin et al. who, using Population Assessment of Tobacco and Health (PATH) data, found no association with composite “high-risk birth” outcome, including LBW, among exclusive vapers (aOR 1.19 [95% CI 0.38–3.73]) [82]. Pesola et al. analysed data from the Pregnancy Trial of E-cigarette and Patches (PREP) and identified no association between a composite EV and nicotine replacement therapy (NRT) group and LBW (aRR 0.64 [95% CI 0.24–1.72]) [85], replicated by Ashford et al. [79]. Both studies incorporated biochemical validation using salivary/urinary cotinine (a nicotine metabolite), anabasine (a tobacco alkaloid biomarker) and exhaled carbon monoxide (CO), although saliva sample return rates were only 55% in Pesola et al. [79,85].

Most comparative analyses reported lower effect estimates for EV compared with CC or dual use groups [70,71,73,76,80–83,85]. Three PRAMS studies showed reduced risk versus CC and dual (concurrent vaping and smoking) users, though vapers still exceeded non-user risk (aRR 1.38 vs CC 1.78 and dual 2.01) [70,76,81]. A UK prospective observational cohort study (EV n = 218) reported a similar pattern [83]. Four studies using different data sources found lower odds of LBW when EV groups were compared to CC groups [71,73,80,82]. Pesola et al. reported higher birthweights in EV/NRT than CC and dual (difference 0.15 kg, [95% CI 0.05–0.25]), supported by biochemical validation [85]. Three studies reported similar effect estimates for EV compared with CC or dual use groups [75,78,79].

In summary, findings were mixed. Nine studies identified increased LBW risk [70–78] and while seven found none [79–85]. Studies reporting no association more frequently incorporated biochemical validation and were more often conducted in independent prospective cohorts. By contrast, PRAMS-based studies dominated the positive associations. Comparative analyses more consistently reported lower effect estimates for EV compared with combustible cigarette or dual use groups.

#### Small for gestational age.

Fifteen studies examined the association between vaping during pregnancy and the risk of small for gestational age (SGA) infants, yielding mixed results. Nine studies reported an increased risk compared to non-use [72,73,75,76,78,86,87,90,92]. Bailey et al. identified reduced third trimester ultrasound growth parameters (estimated weight, head circumference and femur length) in regular vapers [72]. Cardenas et al. identified a 5.1-fold increase in SGA risk for exclusive vapers (aRR 5.1 [95% CI 1.2–22.2]) with biochemical validation, though only recent use was measured so smoking in early pregnancy may have contributed [90]. Nanninga et al. also reported increased risk, though conclusions were limited by a small sample (EV n = 10) and lack of validation [86]. Several large PRAMS analyses reported increased risk (aRR 1.52–2.4), though these rely on self-reported exposure with potential for recall bias and exposure misclassification [73,75,76,78,87,92]. In contrast, six PRAMS-based studies reported no increased risk when compared to non-use [70,77,81,88,93]. McDonnell et al. (EV n = 218), also found no association, however, exposure was self-reported [83].

Comparisons with CC and dual use were variable. Seven studies found lower risk of SGA in EV groups [70,73,76,81,83,88,92]. Hawkins et al. reported EV risk similar to non-smokers (aRR 0.78 [95% CI 0.48–1.27]) but lower than CC (aRR 2.30, [95% CI 2.01–2.63]) and dual use (aRR 1.93, [95% CI 1.31–2.83]) [81]. Similar patterns were identified by five other studies [70,73,76,88,92]. However, six of these seven analyses were derived from PRAMS data. McDonnell et al. observed significantly lower SGA rates in EV (11%) vs CC (28% P < 0.001) and dual use (30.7% p < 0.001) groups, providing findings from an independent prospective cohort [83]. Four studies reported no difference between EV and CC/dual use [75,77,78,93]. Again, all were based on the PRAMS dataset.

Four studies reported higher SGA risk with EV compared to CC or dual use [86,88,90,91]. Cardenas et al. found elevated risk in vapers (aRR 5.1, 95% CI 1.2–22.2) compared to CC (aRR 2.6, [95% CI 0.9–7.2]) and dual use (aRR 2.5, [95% CI 0.7–8.8]) [90]. This prospective cohort study had independent data and biochemical validation but a small sample (EV n = 6) and does not consider the effects of smoking earlier in pregnancy. Cardenas et al. also identified higher SGA rates in dual users who switched to EV (RR 3.2, [95% CI 1.5–6.6]), when compared to smokers who quit (RR 2.9 [95% CI 1.4, 6.3]) [91]. Nanninga et al. also observed increased risk, although generalisability is limited by sample size (EV n = 10) [86]. Wang et al. reported similar findings in a PRAMS study, showing higher odds of SGA in their EV group (aOR 2.4 [95% CI 1.0–5.7]) compared with the CC (aOR 2.3 [95% CI 1.8–2.9]) or dual use groups (aOR 2.3 [95% CI 1.3–4.1]) [87].

Overall, findings were mixed. Several studies reported increased SGA risk among exclusive vapers compared with non-users, while others reported no association. Comparative analyses variably reported lower, similar, or higher effect estimates for EV relative to combustible cigarette or dual use groups. Across studies, exposure was frequently self-reported, comparator definitions varied, and biochemical validation was uncommon. A substantial proportion of analyses were derived from PRAMS data. These methodological characteristics should be considered when interpreting patterns of association.

#### Preterm birth.

Fifteen studies assessed associations between vaping and PTB; only one used biochemical validation [85]. All six studies reporting increased PTB risk were derived from PRAMS data [70,74–78]. Three studies reported higher risk among vapers Ammar et al. (aRR 1.29, [95% CI 1.00–1.65]), Kim et al. (OR 1.86, [95% CI 1.11–3.12]), and Regan & Bombard et al. (adjusted Prevalence Ratio [aPR] 1.69, [95% CI 1.20–2.39]) [70,75,77]. Regan & Pereira found lower PTB risk in quitters than ongoing EV users (aPR 0.70 [95% CI 0.61–0.81]) [78]. Galbo et al. included PTB in a composite adverse outcome, reporting increased odds with vaping (aOR 1.62, [95% CI 1.16–2.26]) [74].

In contrast, nine studies, using a range of study designs, found no association [73,76,81,82,84–88]. These studies included prospective cohorts, PRAMS analyses, and independent datasets. Five PRAMS studies showed no significant association [73,76,81,87,88]. Nanninga (n = 10) reported 0% PTB, though sample size was small (n = 10) [86]. Opondo et al. also found no association (aRR 1.29 [95% CI 0.54–2.04]) [84]. Lin et al. included PTB within a composite “high-risk birth” outcome and found no association (aOR 1.19 [95% CI 0.38–3.73]) [82]. Pesola et al. reported reduced PTB risk in EV/NRT users vs non-users (aRR 0.29 [95% CI 0.12–0.70]), though the non-user group was very small (n = 25) [85].

Comparative analyses examining EV relative to CC or dual use reported variable findings. No study reported higher estimate of risk for PTB in EV groups compared with CC groups. Five reported overlapping confidence intervals between EV and CC use [70,75,78,85,86]. Six studies spanning two US data sets, PRAMS and PATH, reported lower effect estimates for EV compared with combustible cigarette use [81,82,87,88]. Two studies found EV risk to be lower than both CC (aOR 1.30, [95% CI 1.21–1.40], prevalence 4.8% vs 13%) and dual use (aOR 1.43, [95% CI 1.01–2.08], prevalence 4.8% vs 13.1%) [73,76].

In summary, findings were mixed. Some PRAMS-based analyses reported increased PTB risk among exclusive vapers compared with non-use. However, a larger number of studies, including prospective cohorts and independent datasets, reported no association. Comparative analyses reported either overlapping or more frequently lower associations between EV and preterm birth relative to smoking or dual use.

#### Maternal health outcomes.

The association between vaping and maternal mental health was explored by two studies, both using PRAMS data. Choi et al. found no association between vaping and postpartum depression (PPD) compared with non-use (aOR 1.09, [95% CI 0.98–1.29]) [95]. These findings contrast with Wen and Kwak et al., who reported higher PPD prevalence among exclusive vapers compared with non-users (13.2% vs 9.7%). The prevalence of PPD was similar between exclusive vapers and smokers (13.2% vs 13.4%), but lower in vapers compared with dual users (13.2% vs 19.8%) [94].

Maternal gestational weight gain (GWG), which when inadequate is linked to PTB, SGA, and LBW, was examined by Wen et al. via PRAMS data [100]. They reported no association between EV and low GWG compared with non-use (aOR 0.99, [95% CI 0.78–1.27]) and observed a lower likelihood of inadequate GWG compared with CC users [99]. Nanninga et al. examined a composite adverse maternal outcome (Hypertension [HTN], Gestational diabetes [GDM] and Postpartum haemorrhage [PPH]) and found no association with EV compared with non-use (n = 4 p = 0.27) [86]. This was no different when compared to CC or dual users. Overall, evidence on the association between vaping in pregnancy and adverse maternal health outcomes is limited, predominantly derived from PRAMS data, and does not demonstrate consistent associations.

Maternal outcomes remain one of the clearest gaps in the human literature. Most studies focused on fetal or neonatal outcomes, while maternal physiological endpoints were rarely assessed. This is important because animal studies suggest vascular, metabolic and placental mechanisms that could plausibly relate to maternal complications, including endothelial dysfunction, altered arterial stiffness, hypertensive disorders, impaired placental perfusion and gestational metabolic disturbance. Future human studies should therefore incorporate maternal vascular, autonomic, metabolic and placental endpoints alongside fetal and neonatal outcomes, to assess whether the mechanistic pathways identified in animal studies translate to human pregnancy.

#### Fetal and neonatal outcomes.

Pregnancy loss was assessed in three studies. Lin et al., using PATH data (EV n = 41), found no association between vaping and fetal death compared with non-use (aOR 0.33, [95% CI 0.04–2.83]) or between EV and CC groups (aOR 0.56 [95% CI 0.15–2.15]). They did however, observe an association between mint/menthol-flavoured e-liquids and fetal death (aOR 3.27, [95% CI 1.17–9.19] p < 0.05), though case numbers were unreported and adjustment for smoking history was not described [82]. Nanninga et al. reported higher miscarriage rates in their EV group (30% p = 0.01) when compared to non-users and CC users, but their small sample size (EV n = 10), with three using non-nicotine vapes) and lack of biochemical validation limit interpretability [86]. In contrast Le et al. found no association between vaping and miscarriage (aHR 0.64 [95% CI 0.22–1.89]) in a large multi-national cohort study (EV n = 127) [97].

Two small UK studies by Froggatt et al. examined infant neurobehaviour. Their 2020 case-control study (EV n = 10, self-reported exposure) identified neurobehavioural disruption in vapers’ neonates, with abnormal reflexes (p = 0.036), reduced motor maturity (p = 0.036) and borderline self-regulation (p = 0.057). Outcomes were more adverse in CC users compared with exclusive vapers [80]. A 2021 prospective cohort study (EV n = 15, CO validated), found no difference in fetal mouth movements (FMM) between the EV and non-use groups, but heavy CC use (11–20 cigarettes/day) increased FMM (p = 0.038) [96]. Authors noted that diurnal variation limits FMM as a neurobehavioural measure, but both studies reported more pronounced adverse findings in combustible cigarette users.

Four studies assessed neonatal intensive care unit (NICU) admissions. Pesola et al. identified no increased risk of NICU admission in the combined EV/nicotine replacement therapy (EV/NRT) group compared with non-users (aRR 0.57 [95% CI 0.21–1.59]), and equivalent risk compared with CC (6.8%), dual (9.6%), and EV/NRT (9%) groups [85]. An Irish prospective observational cohort study replicated these findings [83] Azar et al. found no association between EV and NICU admission compared with non-use (aOR 0.6 [95% CI 0.37–1.01]) alongside significantly reduced odds of neonatal antibiotic use among exclusive vapers compared with non-users (aOR 0.4 [95% CI 0.21–0.78]). In direct comparisons, combustible cigarette users had higher odds than exclusive vapers of NICU admission (aOR 1.8, 95% CI 1.03–2.99) and neonatal antibiotic use (aOR 2.7 [95% CI 1.35–5.36]) [71]. A Kentucky-based cohort study observed no differences in neonatal respiratory distress (OR 0.75 [95% CI 0.26, 2.21]) and NICU transfer (OR 1.08 [95% CI 0.47, 2.50]) between vaping, smoking and dual use [79].

Three studies examined breastfeeding [83,84,98]. McBride et al. identified reduced odds of breastfeeding beyond 3 months in vapers compared to non-users (OR 0.63 [95% CI 0.44–0.89]) [98]. McDonnell et al. (EV n = 218) similarly observed reduced breastfeeding at discharge (48.6% vs. 61.1% in non-users, p = 0.03), but higher rates compared with CC (27.2%, p < 0.001) and dual users (29.7%, p < 0.001) [83]. In contrast, Opondo et al. found no association (aRR 1.03 [95% CI 0.98 to 1.08]), however, interpretation is limited by small sample size (EV n = 27) and reliance on self-reported exposure [84].

Together, these studies suggest potential neurobehavioural differences associated with EV compared with non-use, however, findings are inconsistent and limited by small sample sizes, retrospective exposure assessment, and limited biochemical validation. Evidence relating to NICU admission was inconsistent, with most studies reporting no statistically significant differences between EV and non-use, and numerically lower point estimates compared with smokers.

#### Methodological characteristics of included human studies.

Across human studies, substantial methodological heterogeneity was observed. Exposure was most frequently classified using retrospective self-report, commonly during the third trimester only, with limited and inconsistent quantification of vaping frequency or dose. Biochemical validation of exposure was rare, incorporated in only a small number of prospective cohorts. Comparator groups varied considerably and included non-use, combustible cigarette smoking, dual use, and in some cases nicotine replacement therapy. However, definitions of EV and dual use were not uniform across studies. Adjustment for confounding was inconsistently reported, and prior smoking history was variably accounted for. A large proportion of positive associations were derived from repeated analyses of PRAMS surveillance data, whereas independent prospective cohorts were fewer in number and often smaller in sample size. These methodological characteristics complicate direct comparison across studies and limit interpretation of apparent consistency or inconsistency in reported associations.

The structured appraisal, presented in S4 Appendix, highlighted substantial variation in exposure classification, confounder adjustment and residual confounding from previous tobacco use across human studies. In several PRAMS analyses, EV referred to self-reported e-cigarette use without concurrent combustible cigarette smoking during the last 3 months of pregnancy, rather than lifelong EV or biochemically confirmed abstinence from tobacco. Other studies included former smokers, quitters, or participants with unclear prior smoking history within lower-exposure comparison groups. Non-use was also inconsistently defined, ranging from no e-cigarette use to no reported nicotine or tobacco use. Dual use was not always analysed separately, and previous smoking was inconsistently captured. These differences limit direct comparison across studies, particularly where apparent EV groups included former smokers or individuals who had switched from combustible cigarettes earlier in pregnancy.

Confounder adjustment also varied substantially. Commonly adjusted variables included maternal age, race or ethnicity, education, BMI, parity, prenatal care, insurance status, WIC participation, income or area-level deprivation, prior preterm birth and pregnancy complications. However, adjustment for prior smoking intensity, switching behaviour, dual use, partner or household smoking, alcohol, cannabis and other substance use was inconsistent. This variation limits comparison across studies reporting adjusted effect estimates and may contribute to residual confounding.

## Discussion

This scoping review included 34 animal and 30 human studies. Animal models frequently reported adverse outcomes of vaping across growth, metabolic, respiratory, neurobehavioural, and vascular domains, with nicotine-containing exposures often demonstrating greater magnitude of effect. These animal studies suggest several biologically plausible pathways through which antenatal vape exposure could affect human pregnancy and offspring health, including oxidative stress, inflammation, endothelial dysfunction, altered placental perfusion, metabolic dysregulation, epigenetic modification and neurodevelopmental disruption [101]. These pathways are relevant to human outcomes such as fetal growth restriction, hypertensive disorders, placental insufficiency, respiratory morbidity and altered neurodevelopment. However, these mechanistic pathways have not yet been confirmed in human pregnancy studies.

Animal findings should therefore be interpreted as mechanistic and hypothesis-generating rather than confirmatory evidence of clinical risk in humans. Direct translation is limited by physiological differences, differing placental structures, differences in nicotine metabolism, and exposure patterns which poorly mimic human usage. For example, many studies used continuous or high-concentration aerosol delivery that may not reflect typical human use patterns. In most models, animals were naïve to nicotine prior to gestational exposure, potentially exaggerating biological responses when compared with humans who vape during pregnancy, many of whom have prior nicotine exposure through smoking.

Compared with animal studies human data were less consistent. Approximately half reported increased risk of adverse outcomes among exclusive vapers compared with non-users, while the remainder found no association. In contrast, among studies comparing EV to smoking, over half suggested reduced risk. One potential explanation for this inconsistency is the variation in exposure classification and dose measurement across human studies. Most relied on single-timepoint, retrospective self-report without quantification of frequency, device type, or nicotine concentration, whereas animal studies use controlled exposure paradigms with defined dosing schedules, allowing clearer attribution of observed effects.

Difficulty in interpreting the literature is consistent with two previous reviews, assessing a very similar set of human studies, which reached divergent conclusions. Ussher et al.’s systematic review found EV generally carried no increased risk compared to non-use, with risk broadly similar to smoking, and dual use comparable to smoking but greater than EV [21]. In contrast, a systematic review and meta-analysis by Deprato et al. reported 53% higher odds of both maternal and neonatal adverse outcomes versus non-use [22]. Both reviews acknowledged substantial variability in exposure classification and residual confounding.

A lack of biochemical validation is a common problem across the studies. The importance of biochemical validation is exemplified by Cardenas et al, who compared self-reported vaping and cigarette smoking status with biochemical measures demonstrating significant underreporting of tobacco use with over half of self-identified ‘never-users’ testing positive for cotinine or other nicotine and tobacco biomarkers [90]. Only five studies used biochemical markers to validate exposure groups. Of these, two identified increased risk of adverse perinatal outcomes [90,91], while three found no increase [79,85,96].

A related limitation was data source clustering. Eighteen of the 30 human studies (60%) were secondary analyses of PRAMS data [70,73–78,81,87–89,92,94,95,98,99]. Although PRAMS provides large, population-based samples of US births, repeated analyses of overlapping PRAMS years should not be interpreted as independent evidence. The same underlying limitations, including reliance on postpartum recall, self-reported smoking and vaping status, limited detail on vaping patterns, incomplete capture of earlier pregnancy smoking, and absence of biochemical validation may result in errors from misclassification and recall bias being reproduced across multiple publications. This clustering may give an inflated impression of evidence volume, particularly for LBW, PTB, SGA, postpartum depression and gestational weight gain outcomes. Three studies were further constrained by very small EV groups (n = 6–10), limiting generalisability [80,86,90].

From a clinical perspective, comparisons between EV, smoking, and medically approved NRT are all relevant. As vaping has been evaluated as a smoking cessation aid, its clinical relevance in pregnancy depends on its safety profile relative to both combustible cigarettes and NRT. While some included studies compared EV to smoking or dual use, few directly compared vaping to NRT. This represents a key evidence gap and limits conclusions regarding the relative safety and clinical role of vaping compared with established smoking-cessation strategies in pregnancy.

In light of the available evidence base, interpretation of relative risk comparisons requires caution. Lin et al. reported a possible association between mint/menthol flavoured vapes and increased odds of fetal death (OR 3.27) [82]. However, this was based on a small subgroup analysis with limited adjustment for confounders and wide confidence intervals and should therefore be interpreted with caution. This tentative finding aligns with non-pregnancy research, where sweet, menthol, and fruit-flavoured e-liquids have been linked to greater DNA damage in a dose-dependent manner [102]. Menthol, at concentrations reported in the blood of maternal vapers, has also been shown to disrupt calcium homeostasis, impair mitochondrial function, and induce embryonic stem cell death [103]. Animal studies similarly suggest flavourings may independently contribute to harm, with non-nicotine exposures linked to obesity, endothelial dysfunction, and cognitive impairment in offspring [45,47,51]. In light of these findings, more pregnancy-specific research on the safety of flavourings is needed. While complete cessation remains the safest option, the limited and exploratory evidence regarding flavour-specific effects underscores the need for targeted investigation before any safety distinctions can be inferred.

Although several studies reported lower levels of association for EV compared with combustible cigarette use across LBW and PTB outcomes, confidence intervals frequently overlapped and potential exposure misclassification limits firm conclusions. For SGA, most comparative analyses reported either lower or comparable point estimates for EV relative to smoking or dual use. However, variability in exposure definition, outcome measurement, and residual confounding preclude definitive conclusions. Importantly, no study demonstrated a statistically significant increase in LBW or PTB risk for EV compared with smoking, but absence of evidence should not be interpreted as evidence of safety.

## Implications

This review highlights important gaps in the evidence on vaping in pregnancy, particularly regarding maternal health outcomes. Recurrent methodological limitations, most notably lack of exposure validation and small sample sizes, underscore the need for more rigorous research.

Future research should adopt prospective designs to minimise recall bias and incorporate biochemical validation to ensure accurate exposure classification, including tests for anabasine to distinguish between smokers and users of other nicotine products. Adequate sample sizes across all groups, including vapers, non-users and smokers, are needed to detect meaningful differences. Maternal physiology, as well as fetal and neonatal outcomes should be assessed. Key perinatal outcomes such as LBW, PTB and SGA require evaluation using standardised definitions and validated measures. Smoking and vaping status before conception should be clearly established, with never-users distinguished from current non-users or quitters to avoid residual confounding. Future studies should also collect partner and household smoking exposure, including second-hand smoke, alongside standard confounders such as socioeconomic status, maternal education, age, ethnicity, BMI, parity, alcohol and cannabis use, other substance use, and relevant obstetric history.

Detailed characterisation of vaping behaviour, including device type, frequency of use, nicotine concentration and flavourings or additives, will be essential given growing evidence of dose-dependent effects and the toxicity of additives. Crucially, studies should compare EV with non-use, combustible cigarette smoking, dual use and medically approved NRT, to establish relative risk and support evidence-based harm reduction strategies. With the increasing popularity of vaping in those of reproductive age, high-quality evidence is urgently needed to guide clinical care and public health messaging.

## Strengths and limitations

The strengths of this review include the broad inclusion criteria spanning both animal and human models and including a range of study designs. The review was guided by the established Joanna Briggs Institute methodology and PRISMA-ScR guidelines. Screening was conducted independently by two reviewers, and the comparison of EV to both smoking and dual use adds clinical relevance.

Limitations include its scoping rather than systematic design, which was intended to map the breadth and characteristics of the evidence rather than generate pooled effect estimates or formal risk-of-bias ratings. Although we undertook a structured interpretability appraisal of human studies, this was descriptive and should not be interpreted as a formal quality score. Although Google Scholar was searched as a supplementary grey-literature source, screening was limited to the first 100 results and relevant grey literature, including conference abstracts or regulatory reports, may therefore have been missed.

## Conclusion

Current evidence on the independent harms of vaping during pregnancy remains inconclusive. Animal studies frequently report adverse biological effects, particularly with nicotine-containing vapes; however, differences in dosing paradigms and species-specific physiology limit direct translation to human pregnancy. Furthermore, animal studies identify biologically plausible mechanisms of harm, but these findings remain hypothesis-generating and have not been consistently confirmed in human pregnancy studies. Whilst human studies do not consistently demonstrate adverse outcomes when EV is compared with non-use, findings are limited by retrospective self-report, inconsistent exposure classification, small sample sizes, and substantial reliance on overlapping datasets. When EV is compared with smoking, most studies report numerically lower or comparable point estimates across common perinatal outcomes. However, heterogeneity in study design, exposure measurement, and residual confounding preclude conclusions regarding relative safety.

As a scoping review, this study aimed to map the breadth, methodological characteristics, and translational gaps within the existing literature rather than to establish causal effect estimates. The evidence base is characterised by substantial methodological variability. Future research should prioritise large prospective cohorts with measures at multiple time points ideally from pre-conception, biochemical validation, clear differentiation between EV and dual use, standardised outcome definitions, and detailed characterisation of device type, nicotine concentration, and flavouring constituents. Longitudinal follow-up into adolescence and adulthood is also required to better understand the potential long-term consequences of maternal vaping in pregnancy on long-term health and wellbeing.

## Supporting information

S1 Appendix Full search strategies. Full database-specific search strategies used to identify human and animal studies.
(DOCX)

S2 Appendix Extracted study characteristics and outcomes. Full extracted study characteristics and outcome data for all included animal and human studies.
(DOCX)

S3 Appendix PRISMA-ScR checklist. Completed PRISMA-ScR checklist for the scoping review.
(DOCX)

S4 Appendix Structured study appraisal and detailed exposure classification. Structured appraisal of included human studies, including exposure timing, exposure group definitions, biochemical validation, previous smoking and dual use, residual confounding, confounder adjustment and overall interpretability.
(DOCX)
