# Supplementary material for: Vaping during pregnancy and maternal, fetal, and infant health outcomes: A scoping review of human and animal studies
Source: PLoS One. 2026 Sep 29;21(9):e0356305. doi: 10.1371/journal.pone.0356305 (PMC13623096; doi:10.1371/journal.pone.0356305)
Supplement: Supplementary file 1 [file pone.0356305.s001.docx]

# S1 Appendix. Full search strategies

Search strategies were developed by the review team and adapted for each database using appropriate controlled vocabulary and syntax. Searches combined terms relating to electronic cigarettes/vaping and pregnancy. All searches were conducted on 10 September 2025. Full database-specific search strategies are provided to ensure reproducibility of the search process.

## EMBASE (Ovid)

| 1. | vaping.ti,ab. |
| --- | --- |
| 2. | (vape or vapes).ti,ab. |
| 3. | e cigarette*.ti,ab. |
| 4. | electronic cigarette*.ti,ab. |
| 5. | vaping/ or vaping associated lung injury/ |
| 6. | electronic cigarette/ or smoking device/ |
| 7. | ELECTRONIC NICOTINE DELIVERY SYSTEM*.ti,ab. |
| 8. | 1 or 2 or 3 or 4 or 5 or 6 or 7 |
| 9. | pregnan*.ti,ab. |
| 10. | pregnancy/ |
| 11. | pregnancy outcome/ |
| 12. | pregnancy outcom*.ti,ab. |
| 13. | 9 or 10 or 11 or 12 |
| 14. | 8 and 13 |
| 15. | limit 14 to english language |

## MEDLINE (Ovid)

| 1. | vaping.ti,ab. |
| --- | --- |
| 2. | (vape or vapes).ti,ab. |
| 3. | e cigarette*.ti,ab. |
| 4. | electronic cigarette*.ti,ab. |
| 5. | Vaping/ |
| 6. | Electronic Nicotine Delivery Systems/ |
| 7. | ELECTRONIC NICOTINE DELIVERY SYSTEM*.ti,ab. |
| 8. | 1 or 2 or 3 or 4 or 5 or 6 or 7 |
| 9. | pregnan*.ti,ab. |
| 10. | pregnancy/ or pregnancy outcome/ |
| 11. | pregnancy outcome*.ti,ab. |
| 12. | 9 or 10 or 11 |
| 13. | 8 and 12 |
| 14. | vaping associated lung injur*.ti,ab. |
| 15. | 8 or 14 |
| 16. | 12 and 15 |
