# Supplementary material for: Vaping during pregnancy and maternal, fetal, and infant health outcomes: A scoping review of human and animal studies
Source: PLoS One. 2026 Sep 29;21(9):e0356305. doi: 10.1371/journal.pone.0356305 (PMC13623096; doi:10.1371/journal.pone.0356305)
Supplement: Supplementary file 2 [file pone.0356305.s002.docx]

# S2: Appendix. Extracted study characteristics and outcomes

These tables present the full extracted data for all included studies. Data were extracted using a standardised form and include study design, exposure characteristics, comparator groups, and reported outcomes. NR indicates not reported.

### Table S1:Animal studies:

Details and Results of Animal studies

| **Author (Year)** | **Species** | **Sample Size** | **Exposure Method** | **Main outcomes** | |
| --- | --- | --- | --- | --- | --- |
|  |  |  |  | **E-cigarette with nicotine** | **E-cigarette without nicotine** |
| 1. **Aboaziza (2023) ^(1)^** | Sprague–Dawley rats | Pups:  n=15 | **E-liquid:** 75% VG / 25% PG **Nicotine:** Tested (0 mg/mL and 18 mg/mL) **Flavourings:** French Vanilla **Exposure:** Whole-body **Regimen:** 1 h/day, 5 days/wk (20 or 60 puffs) | **Pups:**   - Elevated PWV at 3 and 7 months (increased arterial stiffness) - Increased body fat mass, reduced lean mass PND21 - Altered arterial structure and function: decreased elastin, lower elastin/collagen ratio, impaired aortic reactivity to methacholine | **Pups:**   - Elevated PWV at 3 and 7 months - Increased body fat mass reduced lean mass sustained to adulthood (7m). - Altered arterial structure and function: increased collagen, lower elastin/collagen ratio, impaired aortic reactivity to methacholine |
| 2. **Alarabi (2024) ^(2)^** | Mice | NR | **E-liquid:** NR (not reported) **Nicotine:** NR (implied present but concentration not specified) **Flavourings:** NR **Exposure:** Whole-body (implied) **Regimen:** Pre-mating (1 week) + entire gestation (daily, duration/frequency NR) | **Pups:**   - Shortened bleeding and occlusion times. - Altered platelet gene and miRNA expression linked to activation, haemostasis, and inflammation. | NR |
| 3. **AlHarthi (2023) ^(3)^** | C57BL/6 mice | Pups:  n=18 | **E-liquid:** 70% VG / 30% PG **Nicotine:** Tested (0 mg/mL and 25 mg/mL [2.5%]) **Flavourings:** Berry **Exposure:** Whole-body  **Regimen:** 60 min/day × 11 days | **Pups:**   - Reduced marble burying, novel object recognition and cognitive function. - Increased locomotor activity. - Increased adolescent weight gain. - Increased sucrose preference. - Increased nicotine preference by day 6. | **Pups:**   - Reduced marble burying - Increased adolescent weight gain. |
| 4. **Al-Saewalha (2020)^(4)^** | Wistar rats | Pups:  n=40 | **E-liquid:** 70% PG / 30% VG **Nicotine:** Tested (18 mg/mL) **Flavourings:** None **Exposure:** Whole-body **Regimen:** 1 h/day, 6 puffs/min | **Pups:**   - Impaired long-term memory performance - Elevated hippocampal superoxide dismutase (SOD) levels, potentially compensating for increased oxidative stress | NR |
| 5. **Archie (2023)^(5)^** | C57BL/6J mice | Dams:  n=16  Pups:  n=160 | **E-liquid:** NR (Blu™ commercial cartridge) **Nicotine:** Tested (24 mg/mL, 2.4%) **Flavourings:** NR (implied by commercial Blu™ use) **Exposure method:** Direct inhalation  **Regimen:** 6 sessions/day, 32 puffs/session from E5-PD7 | **Dams:**   - Reduced gestational weight gain.   **Pups:**   - Lower birthweight. - Reduced brain expression of ZO-1, claudin-5, occludin, GFAP, GLUT-1, and AQP4. - Impaired short-term memory (novel object recognition) and increased locomotor activity in females. | NR |
| 6. **Aslander (2022) ^(6)^** | CD-1 mice | Dams:  N=6  Pups:  N=30 | **E-liquid:** 50% PG / 50% VG **Nicotine:** Tested (0 mg/mL and 20 mg/mL, 2%) **Flavourings:** None **Exposure method:** Whole-body  **Regimen:** 4 h/day, 5 days/wk | **Pups:**   - Decreased lung compliance. - Histology showed increased fibrotic tissue around airways and vessels, and increased goblet cell hyperplasia. - Females had higher body weight; males had reduced liver weight/body weight ratio. | **Pups:**   - Increased lung stiffness, reduced lung compliance, decreased FEV0.1, and increased alveolar tissue stiffness. - Histology showed increased fibrotic tissue, greater mean linear intercept, and goblet cell hyperplasia. - Females had higher body weight; males had reduced liver weight/body weight ratio. |
| 7. **Awada (2024)^(7)^** | C57BL/6 mice | Pups:  n=25 | **E-liquid:** Commercial Blu™ cartridges (PG/VG ratio NR) **Nicotine:** Tested (0 mg/mL and 13-16 mg/mL)  **Flavourings:** Classic tobacco **Exposure method:** Whole-body inhalation **Regimen:** 3 h/day, 5 days/wk 20 puffs total | **Dams:**   - No significant changes in neuroinflammatory or neurotrophic markers (TNF-α, IL-6, BDNF, NGF)   **Pups:**   - Increased hypothalamic glucose metabolism protein levels (GLUT-1, AMPAR, PPARγ) - Upregulation of genes linked to neuroinflammation (PPARγ, LepRb, MC4R, AMPK, POMC, SLC2A1) | **Dams:**   - No significant changes in neuroinflammatory or neurotrophic markers ( TNF-α, IL-6, BDNF, NGF)   **Pups:**   - Increased hypothalamic glucose metabolism protein levels (GLUT-1, AMPAR, PPARγ) - Upregulation of genes linked to neuroinflammation (LepRb, MC4R, SLC2A1) |
| 8. **Burrage (2020) ^(8)^** | Sprague-Dawley rats | Pups:  N=2–4 per dam | **E-liquid:** NR (composition not specified) **Nicotine:** Tested (0 mg/mL and 18 mg/mL) **Flavourings:** NR **Exposure method:** Whole-body (implied by maternal exposure) **Regimen:** 1 h/day, 5 days/wk (20 or 60 puffs total) | **Pups:**   - Impaired middle cerebral artery (MCA) dilation to acetylcholine (ACh) - Dysfunction reversed by Tempol, suggesting oxidative stress mechanism - No dose-dependent difference between 20 and 60 puffs | **Pups:**   - Impaired MCA dilation to Ach - Dysfunction reversed by Tempol - Impairment similar to nicotine group despite absence of nicotine |
| 9. **Burrage (2021)^(9)^** | Sprague-Dawley rats | Pups:  n=15 | **E-liquid:** 75% VG / 25% PG **Nicotine:** Tested (0 mg/mL and 18 mg/mL) **Flavourings:** French Vanilla **Exposure method:** Whole-body **Regimen:** 1 h/day, 20 puffs total | **Pups:**   - 40% reduction in endothelial-dependent dilation (EDD) in the middle cerebral artery (MCA) - No change in body mass - No effect on 5HT or SNP response | **Pups:**   - 33.33% reduction in endothelial-dependent dilation (EDD) in the MCA - No change in body mass - No effect on 5HT or SNP response |
| 10. **Cahill & Johnson (2022) ^(10)^** | BALB/c mice | Dams:  n=17  Pups:  n = 134 | **E-liquid:** Commercial JUUL mint pods **Nicotine:** Tested (5% nicotine salt) **Flavourings:** Mint **Exposure method:** Whole-body **Regimen:** 1 h/day × 20 days | **Dams:**   - Dysregulation of placental genes linked to oxidative stress and hypoxia - Mild reduction in placental HSD2 enzyme expression   **Pups:**   - 10.2% lower birthweight and shorter crown–rump length - Reduced lung airspace and disrupted architecture - Upregulated inflammatory markers and pro-inflammatory lung environment - Increased allergic inflammation after dust mite exposure - Altered expression of Wnt pathway and chromatin modifier genes - Increased methylation of *Il10ra* gene (asthma-associated) | NR |
| 11. **Cahill & Gartia (2022) ^(11)^** | BALB/c mice | Dams:  n=13  Pups:  n=80 | **E-liquid:** 50% PG / 50% VG **Nicotine:** Tested (36 mg/mL) **Flavourings:** Cinnamon Fireball (contains cinnamaldehyde) **Exposure method:** Whole-body  **Regimen:** 2 h/day × 20 days | **Pups:**   - Lower birth weight sustained to postnatal day 5 - Decreased lung fibrillar collagen and increased Newtonian resistance (airway narrowing) - Dysregulation of Wnt and chromatin modification genes (18 in males, 20 in females), associated with lung development and asthma/ emphysema pathogenesis in humans | NR |
| 12. **Chen & Chapman (2018) ^(12)^** | BALB/c mice | Dams: n=24 Pups: n=60 | **E-liquid:** 50% PG / 50% VG **Nicotine:** Tested (0 mg/mL and 18 mg/mL) **Flavourings:** Tobacco (commercial blend) **Exposure method:** Whole-body  **Regimen:** 2×15-min sessions/day | **Dams:**   - Increased IL-1β, IL-6, and TNF-α in lung tissue.   **Pups:**   - Lower weanling weight (PND 20) - Altered lung cytokine profile (increased TNF-α and decreased IL-1β) - Global DNA hypermethylation. | **Dams:**   - Increased IL-1β, IL-6, and TNF-α in lung tissue.   **Pups:**   - Higher weanling weight (PND 20) - Altered lung cytokine profile (increased TNF-α and decreased IL-1β) - Global DNA hypermethylation. |
| 13. **Chen & Nguyen (2018)^(13)^** | BALB/c mice | Pups:  n=19 | **E-liquid:** Commercial tobacco-flavoured (exact PG/VG ratio NR) **Nicotine:** Tested (0 mg/mL and 18 mg/mL) **Flavourings:** Tobacco (commercial blend) **Exposure method:** Whole-body  **Regimen:** 2×30-min sessions/day for 6 weeks pre-mating + gestation/lactation | **Note:** Dams were initially exposed to cigarette smoke, then switched to nicotine-containing e-cigarettes during pregnancy.   **Pups:**   - Lower weanling weight and abdominal fat mass. - Nicotine-containing e-vapour replacement normalized SE-induced dysregulation of central metabolic regulators. - No increased mRNA expression of central metabolic regulators. | **Pups:**   - Higher weanling weight and greater abdominal fat mass. - Elevated brain mRNA expression of metabolic regulators (NPY, iNOS, Ob-Rb, MC4R). |
| 14. **Church (2020) ^(14)^** | CD-1 mice | Dams:  n=27  Pups:  n=135 | **E-liquid:** 50% PG / 50% VG **Nicotine:** Tested (0 mg/mL and 16 mg/mL) **Flavourings:** None (base solution only) **Exposure method:** Whole-body **Regimen:** 3 h/day, 7 days/wk | **Dams:**   - No differences in maternal weight - No differences in litter size   **Pups:**   - No differences in fetal weight - Increased locomotor activity (elevated plus maze, open field test) - Impaired memory performance (novel object recognition test) - Altered stress coping behaviour: reduced immobility and increased swim time (forced swim test) - Reduced anti-inflammatory cytokines in brain regions associated with neurodevelopment | **Dams:**   - No differences in maternal weight or litter size.   **Pups:**   - No differences in fetal weight - Impaired memory performance (novel object recognition test) - Reduced anti-inflammatory cytokines in brain regions associated with neurodevelopment |
| 15. **Kishinchand (2022) ^(15)^** | C57BL/6 mice | Dams: n=9 Pups: n=27 | **E-liquid:** 50% PG / 50% VG **Nicotine:** Tested (0 mg/mL and 20 mg/mL) **Flavourings:** None (vehicle only) **Exposure method:** Whole-body  **Regimen:** 4 h/day, 5 days/wk | **Pups:**   - Lower birth weight. - Impaired craniofacial development: reduced facial width/length, palatal width, and cranial base length. | **Pups:**   - Mild craniofacial changes: narrower palates. |
| 16. **Li (2020)^(16)^** | C57BL/6 mice | Dams: n=6  Pups: n=24 | **E-liquid:** 50% PG / 50% VG **Nicotine:** Tested (0 mg/mL and 18 mg/mL) **Flavourings:** Tobacco (commercial blend) **Exposure method:** Whole-body  **Regimen:** 2 sessions/day × 6 weeks pre-mating + gestation/lactation | **Dams:**   - No difference in blood glucose, insulin, or triglycerides. - Elevated metabolic dysfunction markers and hepatic mRNA expression. - No rise in pro-inflammatory liver markers. - Reduced hepatic protein autophagy.   **Pups:**   - Glucose intolerance without significant hepatic inflammation or mitochondrial injury. - Increased gluconeogenesis marker expression. - No change in body weight or plasma markers. | **Dams:**   - Elevated liver weight, ALT, and glucose metabolic markers. - Multiple elevated pro-inflammatory liver markers. - Hepatic mitochondrial damage (reduced mtDNA copy number), increased hepatocyte damage, reduced antioxidants.   **Pups:**   - Increased liver weight. - Increased expression of glycolysis and gluconeogenesis markers. - Glucose intolerance with increased hepatic oxidative stress and inflammation. - Evidence of mitochondrial injury and increased autophagy. |
| 17. **McAlinden (2017)^(17)^** | BALB/c mice | NR | **E-liquid:** NR (composition not specified) **Nicotine:** Tested (presence/absence) **Flavourings:** NR **Exposure method:** Whole-body **Regimen:** Pre-mating through gestation/lactation | **Pups:**   - Increased eosinophilia and airway resistance (indicating enhanced allergic asthma features). - Increased mitochondrial oxygen consumption. - Effects similar to those seen with combustible cigarette exposure. | **Pups:**   - No increase in eosinophilia or airway resistance. - Less pronounced asthma phenotype compared to nicotine group. |
| 18. **Mills (2023) ^(18)^** | Rats | Pups:  n=15 | **E-liquid:** 50% PG / 50% VG **Nicotine:** Tested (0 mg/mL and 50 mg/mL) **Flavourings:** None **Exposure method:** Whole-body  **Regimen:** 60 puffs/day (1.5 h/day, 5 days/wk) | **Pups:**   - Lower birth weight (only seen in females in the 30W group) - Higher body weight in adulthood (obesity) by 6–12 months. - No cerebrovascular outcome data available. | **Pups:**   - Lower birth weight at postnatal day 21 (16–18% reduction). - Higher body weight in adulthood (obesity) by 6–12 months. - Impaired cerebrovascular function (reduced MCA reactivity at 12 months). |
| 19. **Mills & Corbin (2025)^(19)^** | Sprague-Dawley rats | Dams: n=37 Pups: n=210 | **E-liquid:**  50% PG / 50% VG **Nicotine:** Tested (0 mg/mL and 50 mg/mL) **Flavourings:** None **Exposure method:**  Whole-body **Regimen:** 60 puffs/day (1 puff/3 min over 90 min), 5 days/week, from Gestation Day 2 to 21. | **Dams:**   - Increased pup resorption, smaller litter size.   **Pups:**   - Effects worse at 30W than 5W. - Higher 6- & 12-month body mass - Blunted MCA response to ACh, improved in response to TEMPOL sex selective improvement with Febuxostat (better in males). Persistent to 12 months. Indicates oxidate stress mechanism. - Short term memory loss (lack of NOR), decrease in maze escape hatch interaction. - Greater anxiety in OFT. - Increased plasma Extracellular Vesicles. - Altered expression of clock genes (Arnt1, Per1, Per3, Clock, Cry1, Cry2) in brain tissue of 1-month-old offspring, more pronounced. - Increased silver staining (neuronal damage), increased astrocyte activation and astrocyte-endothelial cell interactions at 12 months. - Decreased SIRT1 and increase in NOX1, Aβ1-40, and Aβ1-42 at 12 months. | **Dams:**   - Increased pup resorption, smaller litter size.   **Pups:**   - Effects worse at 30W than 5W. - Higher 6- & 12-month body mass. - Blunted MCA response to ACh, improved in response to TEMPOL sex selective improvement with Febuxostat (better in males). Persistent to 12 months. - Short term memory loss (lack of NOR). - Greater anxiety in OFT. - Increased plasma Extracellular Vesicles. - Altered expression of clock genes (Arnt1, Per1, Per3, Clock, Cry1, Cry2) in brain tissue of 1-month-old offspring. - Increased astrocyte activation at 12 months. - Decreased SIRT1 and increase in NOX1, Aβ1-40, and Aβ1-42 at 12 months. |
| 20. **Mills & Dakhallah (2025)^(20)^** | Sprague-Dawley rats | Dams: n=37 Pups: n=347 | **E-liquid:**  50% PG / 50% VG **Nicotine:** Tested (0 mg/mL and 50 mg/mL) **Flavourings:** None **Exposure method:**  Whole-body **Regimen:** 60 puffs/day (1 puff every 1.5 min over 90 min), 5 days/week, from Gestation Day 2-4 until Day 21. | **Dams:**   - Elevated plasma Extracellular Vesicles. - Reduced GD20 maternal weight gain. - Increased pup resorption and pup death. - Effects partially reversed by TEMPOL suggesting oxidative stress mechanism. - Decreased SIRT1 expression. - Blunted MCA response to ACh. 24% decline in 5W. 50% decline in 50W. - Decreased plasma prolactin. | **Dams:**   - Elevated plasma Extracellular Vesicles. - Reduced GD20 maternal weight gain. - Increased pup resorption and pup death. - Effects partially reversed by TEMPOL suggesting oxidative stress mechanism. - Decreased SIRT1 expression. - Blunted MCA response to ACh. 21% decline in 5W. 24% decline in 51% decline in 50W. - Decreased plasma prolactin. |
| 21. **Nassabeh (2023)^(21)^** | Sprague-Dawley rats | Dams: n=19 Pups: n=210 | **E-liquid:** 50% PG / 50% VG **Nicotine:** No **Flavourings:** None **Exposure method:** Whole-body  **Regimen:** 60 puffs/day, 5 days/wk | **Pups:**   - Impaired endothelial function with reduced nitric oxide bioavailability. - Increased pulse wave velocity indicating elevated arterial stiffness. - Aortic dilation response significantly blunted. | NR |
| 22. **Nguyen (2018) ^(22)^** | BALB/c mice | Dams: n=24   Pups: n=126 (male pups only) | **E-liquid:** 50% PG / 50% VG **Nicotine:** Tested (0 mg/mL and 18 mg/mL) **Flavourings:** Tobacco flavour **Exposure method:** Whole-body **Regimen:** 2 × 15 min sessions/day, 6 weeks pre-pregnancy through lactation | **Pups:**   - Impaired short-term memory, reduced anxiety-like behaviour, and increased locomotor activity. - Downregulation of epigenetic regulators (DNMT1, DNMT3A, HDAC1, and MECP) | **Pups:**   - Impaired short-term memory, reduced anxiety-like behaviour, and increased locomotor activity. - Increased DNA methylation and downregulation of epigenetic regulators (DNMT1, DNMT3A, HDAC1, and MECP) |
| 23. **Noel (2020)^(23)^** | BALB/c mice | Dams: n=48  Pups: n=58 | **E-liquid:** 50% PG / 50% VG **Nicotine:** Tested (36 mg/mL) **Flavourings:** Cinnamon **Exposure method:** Whole-body  **Regimen**: 2 h/day | **Dams:**   - Reduced placental growth factor (PLGF) - Increased respiratory elastance, indicating stiffer lungs and increased work of breathing. - Dysregulation of respiratory inflammation-related genes.   **Pups:**   - Reduced birth weight. - Increased lung tissue-to-airspace ratio. - Dysregulation of 75 genes/proteins involved in lung development and inflammation, consistent with lung hypoplasia. | NR |
| 24. **Noel (2023) ^(24)^** | BALB/c mice | Dams: n=24  Pups: n=24 | **E-liquid:** 50% PG / 50% VG **Nicotine:** Tested (18 mg/mL) **Flavourings:** Vanilla **Exposure method:** Whole-body  **Regimen:** 2 h/day for 20 gestational days | **Pups:**   - **PND0**: Sex-specific dysregulation of lung gene expression. Males had 88 dysregulated genes (notably in CD28 signalling, immune activation); females had 65 (mostly related to NRF2-mediated oxidative stress). - **PND28**: Reduced tidal volume, increased neutrophilic inflammation and mucus production after allergen challenge. Upregulation of asthma-related genes. | NR |
| 25. **Orzabal (2019) ^(25)^** | Sprague-Dawley rats | Dams: n=15  Pups: n=24 | **E-liquid:** 80% PG / 20% VG **Nicotine:** Tested (0 mg/mL or 50 increased to 100 mg/mL) **Flavourings:** None **Exposure method:** Whole-body  **Regimen:** 3h/day, 5d/wk (GD5-19) or 2h/day, 5d/wk (GD5-21 + PND4-9) | **Dams:**   - Uterine artery blood flow reduced by 49.5% - No changes in weight, heart rate, or litter size   **Pups:**   - Umbilical artery blood flow reduced by 65.3% - Smaller overall size: fetal weight 46.6% lower and crown-rump length 23.8% shorter - Postnatal weight lower (PND 4–10); crown-rump length shorter at PND 10 | **Dams:**   - No changes in blood flow, weight, heart rate, or litter size   **Pups:**   - No differences in growth or crown-rump length compared to controls |
| 26. **Orzabal (2021) ^(26)^** | Sprague-Dawley rats | Dams: n=18  Pups: n=36 (1 male + 1 female per dam) | **E-liquid:** 80% PG / 20% VG **Nicotine:** Tested (0 mg/mL or 50 increased to 100 mg/mL) **Flavourings:** None **Exposure method:** Whole-body  **Regimen:** 3h/day, 5d/wk (GD5-20) | **Dams:**   - Dysregulated 13 of 22 amino acids in maternal plasma, including increased glutamine (crucial for nitrogen transport and immune function), branched-chain amino acids (BCAAs – essential for growth and metabolism), and ornithine (key in urea cycle and nitrogen metabolism).   **Pups:**   - Reduced fetal weight (36.7%) and crown-rump length (16.6%) compared to controls. - Dysregulation of 3 amino acids in fetal plasma (increased glutamate, phenylalanine, ornithine). - Dysregulated amino acids in fetal lungs (11 of 22 in males, 9 of 22 in females), including elevated BCAAs and ornithine. | **Dams:**   - No significant changes in maternal or fetal plasma amino acid profiles.   **Pups:**   - Mild dysregulation in fetal lungs, with increased threonine in both sexes (important for surfactant, mucus production, and tissue repair). |
| 27. **Orzabal (2022) ^(27)^** | Sprague-Dawley rats | Dams: n=15 Pups: n=18 | **E-liquid:** 80% PG / 20% VG **Nicotine:** Tested (0 mg/mL or 50 increased to 100 mg/mL) **Flavourings:** None **Exposure method:** Whole-body  **Regimen:** 3h/day, 5d/wk (GD5-20, excluding GD9,10,16,17) | **Dams**:   - No difference in maternal weight between groups at GD21.   **Pups**:   - Fetal growth restriction (25% lower weight, 12% shorter crown–rump length). - Marked gene dysregulation in fetal lungs (3,306 genes; 2,322 upregulated, 984 downregulated). - Structural lung changes (emphysema-like phenotype: increased mean linear intercept, reduced radial alveolar count). - Trends towards impaired lung mechanics (higher respiratory resistance, lower static compliance). | **Dams**:   - No difference in maternal weight at GD21.   **Pups**:   - Mild gene expression changes (48 genes dysregulated). - No significant changes in lung structure or function. |
| 28. **Ozekin (2023) ^(28)^** | C57BL/6 & Kcnj2KO mice | Pups: n=68 | **E-liquid:** NR (commercial cartridge) **Nicotine:** Tested (0 mg/mL or 24 mg/mL 2.4% freebase) **Flavourings:** NR **Exposure method:** Whole-body  **Regimen:** 4h/day (4 cartridges/day) from E0-E18 | **Pups:**   - Reduced airspace area indicating impaired lung development - 1,483 genes upregulated and 1,080 downregulated in fetal lungs (RNAseq), including pathways linked to mitochondrial dysfunction, protein degradation, metabolism, and DNA damage - Reduced femur and humerus length; smaller craniofacial features (premaxilla area/length, mandible size) - Skeletal abnormalities less severe in Kcnj2KO/KO mice | **Note: Mice with complete Kcnj2 gene knockout exhibited attenuated skeletal abnormalities, indicating that Kcnj2 may mediate the teratogenic effects of nicotine.**  **Pups:**   - No significant changes in lung morphology or gene expression - Mild skeletal differences not statistically significant |
| 29. **Reynolds (2025)^(29)^** | C57BL/6J mice | Dams: n=13 Pups: n=83 | **E-liquid:**  30% PG / 70% VG **Nicotine:** Tested (5 mg/mL) **Flavourings:** None  **Exposure method:**  Whole-body **Regimen:** 2.75h/day 7 days/week | **Dams:**   - **Arrhythmias**: Increased ventricular premature beats during labour, persisting at 3 weeks postpartum. - **Cardiac function**: Accelerated ventricular repolarization (shorter QT, QTc, JT, JT/QT). Increased ejection fraction (higher CO) indicating increased inotropy and sympathetic burden. - Increased 3-Methoxytyramine (3MT) suggesting a sympathetic dominance mechanism.   **Pups:**   - Lower birth weight (1.14 g vs 1.29 g, p=0.02). | **Dams:**   - NR   **Pups:**   - NR |
| 30. **Richlak (2025)^(30)^** | C57BL/6 mice | Dams: n=21 Pups: n=140 | **E-liquid:**  50% /50% PG/VG and 30% /70% PG/VG **Nicotine:** No  **Flavourings:** None  **Exposure method:** Whole-body  **Regimen:** 4h/day 5 days/week | **Pups:**   - NR | **Pups:**   - Significant effects with 30/70 PG/VG exposed group not seen with 50/50 PG/VG group. - Impaired craniofacial development: reduced mid-facial width, posterior facial width, facial length, facial height, nasal length, cranial width, cranial height all in 30/70 group. - Reduced PND 14 weights in 30/70 group. |
| 31. **Sifat (2020)^(31)^** | CD1 mice | Pups: n=121 | **E-liquid:** NR (Blu™ commercial cartridge) **Nicotine:** Tested (24 mg/mL) **Flavourings:** NR **Exposure method:** Smoking machine (SCSM) **Regimen:** 6 sessions/day (32 puffs/session) from E5-PD7 | **Pups:**   - Reduced body weight at PND8 - Increased susceptibility to hypoxic-ischemic brain injury (larger infarcts, delayed reflex recovery) - Impaired neuronal viability, glucose uptake, and ATP production - Decreased cerebral glucose transporter expression - Hyperactivity with impaired memory and motor coordination | NR |
| 32. **Walayat (2021)^(32)^** | Sprague-Dawley rats | Pups: n=93 | **E-liquid:** NR (BluPlus Cig commercial cartridge) **Nicotine:** Tested (24 mg/mL, 2.4%) **Flavourings:** NR **Exposure method:** Whole-body  **Regimen:** 12h/day (1 episode/hour) from E4-E20 | **Pups:**   - Reduced body and brain weight; increased brain-to-body ratio - Greater infarct size following hypoxic-ischemic injury (particularly in males) - Elevated reactive oxygen species (ROS) and global DNA methylation - Decreased NOX2 (oxidative defence), ATG5 & LC3β (autophagy), and Sirt1 (neuroprotection) expression - ATG5 knockdown further increased injury severity, suggesting impaired autophagy contributes to vulnerability | NR |
| 33. **Wang & Sundar (2020)^(33)^** | CD-1 mice. | Pups: n=48 | **E-liquid:** 50% PG / 50% VG. **Nicotine:** Tested (0 mg/mL and 16 mg/mL, 1.6%). **Flavourings:** None **Exposure method:** Whole-body  **Regimen:** 3 h/day, 5 days/week GD0.5–GD21. | **Pups**:   - Some sex selective changes. - Elevated PAI-1 (extracellular matrix [ECM] remodeling and fibrotic changes) - Decreased MMP9 (ECM deposition and fibrosis). - *Males*: Decreased E-cadherin (cell-adhesion protein, fibrosis), elevated PPARγ (pro-inflammatory cytokine suppressor, protective), ACTA2 (myogenic marker, fibrotic) CNN1 (myofibroblast transition). Increased collagen deposition. | **Pups**:   - Sex selective changes - *Females*: Increased fibronectin (fibrosis), LEF-1 (fibroblast proliferation and ECM production, fibrosis), HDAC-1 (fibrosis). Decreased ADRP (lipogenic marker, risk of oxidative stress). - *Males*: Increased collagen deposition. |
| 34. **Yu (2024) ^(34)^** | Sprague-Dawley rats | Pups: n=18 | **E-liquid:** 50% PG / 50% VG (Gold Leaf flavour) **Nicotine:** Tested (0 mg/mL or 24 mg/mL, 2.4%)  **Flavourings:** Gold Leaf **Exposure method:** Whole-body  **Regimen:** 12h/day (2 episodes/hour, 6 puffs/episode) from E6-PND21 | **Pups**:   - Reduced proliferation, migration, and wound healing activity of bone marrow stem cells (BMSCs) - Downregulation of lipogenic markers, upregulation of myogenic markers, reduced Wnt signalling (β-catenin, LEF-1). - Suggesting reprogramming of BMSCs toward a fibrotic phenotype and impaired tissue repair | **Pups**:   - No significant changes in BMSC proliferation, migration, or wound healing - Minimal alterations in molecular markers |

NR = Not reported; PG = Propylene glycol; VG = Vegetable glycerine; LBW = Low birth weight; PTB = Preterm birth; SGA = Small for gestational age

# Table S2: Human studies:

Details and Results of human studies

| **Author and Year** | **Design and Sample Size** | **Biochemical Validation** | **Vaping Characteristics including usage frequency** | **Outcomes** | |
| --- | --- | --- | --- | --- | --- |
|  |  |  |  | **Exclusive vaping (EV) compared to non-use** | **Exclusive vaping compared to combustible cigarette (CC) use and dual use** |
| 1. **Ammar (2023)^(35)^** | Cross-sectional surveillance study using US PRAMS data  *USA*  n=190,707  EV n=206  CC n=14752  Dual n=1404 | No | Self-reported exclusive vaping, use of combustible cigarettes or dual use:  During the 3 months before pregnancy  During the last 3 months of pregnancy.  **Usage frequency**: Usage quantified to <1 day/week, 1-6 days/week, >1 per day. | **Low birth weight (LBW)**: Increased risk with EV (aRR: 1.38, [95%CI: 1.09, 1.75]) reduced risk with cessation during pregnancy  **Preterm birth (PTB)**: Increased risk with EV (aRR: 1.29, ]95%CI: 1.00, 1.65]); reduced risk with cessation  **Small for gestational age (SGA)**: No association with EV (aRR: 1.04, [95%CI: 0.76, 1.44]) | **LBW**: Risk with EV lower than CC (aRR: 1.78, 95%CI: 1.66, 1.91) and dual use (aRR: 2.01, [95%CI: 1.63, 2.48])  **PTB**: risk with EV equivalent to CC (aRR: 1.29, [95%CI: 1.18, 1.40]) and higher than dual use (aRR: 1.19, [95%CI: 0.97, 1.48])  **SGA**: risk with vaping lower than CC (aRR: 2.02, 95%CI: 1.88, 2.17) and dual use (aRR: 2.27, [95%CI: 1.90, 2.72]) |
| 2. **Ashford (2021)^(36)^** | Prospective longitudinal cohort study.  *Kentucky, USA*  n=218  EV n=21  CC n=106  Dual n=91  Non-use/quitters n=25 | Yes – urinary and serum cotinine | Participants classified as exclusive vaping, CC, dual use, or quitters during pregnancy (quitters used as non-use comparator).  **Usage frequency**: NR | Composite quit vs no switch outcome so individual outcome data on quit vs EV no switch limited.  **LBW**: No association with EV (EV BW 2873g vs EV quit 2987g difference of 114g not significant)  **PTB**: NR  **SGA**: NR  **Other:**  **Respiratory distress**: NR  **NICU transfer**: NR | Extrapolated from switch vs no switch data, if significantly altered OR with switching.  **LBW**: Risk with EV equivalent to CC and dual use (difference 182.79g p=0.4462)  **PTB**: NR  **SGA**: NR  **Other:**  **Respiratory distress**: Risk with EV equivalent to CC and dual use (OR 0.75 [95%C I 0.26, 2.21])  **NICU transfer**: Risk with EV equivalent to CC and dual use (OR 1.08 [95%C I 0.47, 2.50]) |
| 3. **Azar (2024)^(37)^** | Retrospective cohort study. Statewide collaborative of 70 maternity hospitals in Michigan (OBI registry). Jan 2021–Jul 2022.  *USA*  n=29,103  EV n=493  CC n=2,216 | No | Self-reported exclusive e-cigarette use, exclusive cigarette use, or non-use during pregnancy. Dual users and those with alcohol/opioid use excluded.  **Usage frequency**: NR | **LBW**: Increased risk with EV (aOR 1.6 [95% CI 1.03-2.61])  **PTB**: NR  **SGA**: NR  **Other:**  **NICU admission**  No association with EV (aOR 0.6 [95% CI 0.37-1.01])  **Need for neonatal antibiotics**  Lower risk with EV vs control (aOR 0.4 [95% CI 0.21-0.78]) | **Direct comparison of CC vs EV**  **LBW**: Risk with EV lower than CC (aOR 1.4 [95% CI 0.83-2.47])  **PTB**: NR  **SGA**: NR  **Other:**  **NICU admission**  Risk with EV lower than CC (aOR 1.8 [95% CI 1.03-2.99])  **Need for neonatal antibiotics**  Risk with EV lower than CC (aOR 2.7 [95% CI 1.35-5.36]) |
| 4. **Bailey (2024)^(38)^** | Retrospective cohort study, single site  *USA*  n=191  EV n=20 | No  Urine drug screening used to exclude other substance use. | Regular self-reported exclusive vaping from early pregnancy through at least the third trimester; no combustible cigarette or other substance use.  **Usage frequency**: NR | **LBW:** Increased risk with EV, birth weight −12.8 (6.5) (p= 0.025), length −19.3 (6.9) (p= 0.003), Head circumference −13.3 (7.4) (p= 0.037)  **PTB:** NR **SGA:** Increased risk with EV  Third trimester estimated fetal weight −17.2 (8.9) (p= 0.028), femur length −28.5 (9.3) (p= 0.001), head circumference −11.1(8.6) (p= 0.048) | **LBW**: NR  **PTB**: NR  **SGA**: NR |
| 5. **Cardenas (2019)^(39)^** | Prospective cohort study.  *USA*  n=199  EV n=6  CC n=56  Dual n=17 | Yes  Salivary cotinine, CO, hair nicotine | Self-reported exclusive vaping, dual use (vaping and tobacco cigarettes), or exclusive cigarette smoking within the month prior to enrolment.  **Usage frequency**: vaping 1-10days/month, 1-9 times/month | **LBW:** NR.  **PTB:** NR **SGA:** EV group had 5.1-fold increased risk (aRR 5.1 [95% CI 1.2–22.2]) | **LBW**: NR  **PTB**: NR  **SGA**: risk with EV higher than CC (aRR 2.6 [95%CI 0.9–7.2]) and dual use (aRR 2.5 [95%CI 0.7–8.8]) |
| 6. **Cardenas (2020)^(40)^** | Population-based retrospective cohort study using 2016–2017 Arkansas PRAMS data  *USA*  n = 1,594 (weighted = 64,981 births)  EV n=18  CC n=372  Dual n=100 | Yes  Hair nicotine and plasma cotinine used in sensitivity analyses | Self-reported exclusive vaping, use of combustible cigarettes or dual use:  During the 3 months before pregnancy  During the last 3 months of pregnancy  **Usage frequency**: NR | **LBW:** NR  **PTB:** NR  **SGA:** EV risk not calculable. Continued exclusive vaping in dual users after smoking cessation linked to higher SGA risk (RR 3.2, [95% CI: 1.5–6.6]) | **LBW**: NR  **PTB**: NR  **SGA**: CC risk increased (aRR 1.6 [95%CI 1.0. 2.6]) dual use risk increased (aRR 1.8 [95%CI 1.0, 3.4]).  Dual group which switched to EV had higher risk of SGA than CC group who stopped smoking (RR: 2.9 [95% CI: 1.4, 6.3]) |
| 7. **Choi (2024)^(41)^** | Cross-sectional analysis of national PRAMS data (2016–2019)  *USA*  n = 58,950 (weighted)  EV 3 months before pregnancy n= 3,879  EV Last 3 months of pregnancy n= 3,834 | No | Self-reported exclusive vaping, use of combustible cigarettes or dual use:  During the 3 months before pregnancy  During the last 3 months of pregnancy  **Usage frequency**: Usage quantified to <1 day/week, 1-6 days/week, >1 per day. | **LBW:** NR **PTB:** NR  **SGA:** NR **Other:**  **Postpartum depression (PPD)**. EV group had higher odds of PPD in unadjusted analysis (OR 1.63, [95% CI: 1.42–1.87]), but this was not significant after adjustment (aOR 1.09, [95% CI: 0.98–1.29]). | **LBW**: NR  **PTB**: NR  **SGA**: NR  **Other:** NR |
| 8. **Cui (2022)^(42)^** | Retrospective cohort study using PRAMS data (2012–2018)  *USA*  n=239,292  EV n=357  CC n=24853  Dual n=688 | No | Self-reported exclusive vaping, use of combustible cigarettes or dual use:  During the 3 months before pregnancy  During the last 3 months of pregnancy  **Usage frequency**: Usage quantified to <1 day/week, 1-6 days/week, >1 per day. | **LBW**: EV group had increased risk (aOR 1.68, [95% CI: 1.15–2.47]) **PTB**: No association with EV (aOR 1.17 [95%CI 0.74-1.83]) **SGA**: EV group had increased risk (aOR 1.59, [95% CI: 1.02–2.68]) | **LBW**: risk with EV lower than CC (aOR 1.95 [95% CI 1.84-2.07]) equivalent to dual use (aOR 1.68 [95% CI 1.15-2.47])  **PTB**: : risk with EV lower than CC (1.30 [95% CI 1.21-1.40]) and dual use (aOR 1.43 [95%CI 1.01-2.08])  **SGA**: : risk with EV lower than CC (aOR 2.51 [95% CI 2.36-2.69]) and dual use (aOR 3.46 [95% CI 2.49-4.79]) |
| 9. **Froggatt (2020)^(43)^** | Case-control study.  *UK*  n=83 infants  EV n=10  CC n=29 | No | Self-reported vaping or combustible cigarette use at 32 weeks.  **Usage frequency**: NR | **LBW:** No association with EV (Head circumference [HC] and birthweight assessed).   **PTB:** NR **SGA:** NR **Other:**  EV exposed infants had neurobehavioural disruption  Great number of **Abnormal reflexes** (p=0.002)  Reduced **Self-regulation** (p = 0.057)  Reduced **Motor maturity** (p = 0.036) | **LBW**: EV had lower risk then CC group who had lower birthweight (p=0.021) and HC (p=0.008)  **PTB**: NR  **SGA**: NR  **Other**:  EV had better outcomes then CC group  CC **Abnormal reflexes** (p=0.001)  CC **Self-regulation** (p= 0·01)  CC **Motor maturity** (p=0.002) |
| 10. **Froggatt (2021)^(44)^** | Prospective observational cohort.  *UK*  n = 123 women  EV n=15  CC n= 13 | Yes  Exhaled Carbon monoxide for cigarette exposure only | Self-reported vaping, or light or heavy use of combustible cigarettes at 32 and 36 weeks.  **Usage frequency**: vaping nicotine doses 3-16mg, smokers 11-20 cigarettes/day | **LBW:** NR  **PTB:** NR  **SGA:** NR  **Other:**  **Fetal mouth movements** to assess neurobehavioural effects. No significant differences (p=0.289). | **LBW**: NR  **PTB**: NR  **SGA**: NR  **Other**:  **Fetal mouth movements** EV had better outcomes then CC group. Heavy smokers (11-20 cigarettes today had worse outcomes with higher levels of fetal mouth movements (p=0.038) |
| 11. **Galbo (2022)^(45)^** | Retrospective cohort study using PRAMS 2016–2017 data.  *USA*  N=71,940 women  EV n= 859 | No | Self-reported exclusive vaping, use of combustible cigarettes or dual use:  During the 3 months before pregnancy  During the last 3 months of pregnancy  **Usage frequency**: NR | **LBW**: Included in composite “unfavourable birth outcomes” but not reported separately.  **PTB**: Included in composite “unfavourable birth outcomes” but not reported separately.  **SGA**: NR  **Other**:  **Composite unfavourable birth outcomes** (PTB, LBW, prolonged PN stay) Increased 62% in EV group  (aOR 1.62, 95% CI: 1.16–2.26, p=0.005) | **LBW**: NR  **PTB**: NR  **SGA**: NR  **Other**: NR |
| 12. **Hawkins (2022)^(46)^** | Cross-sectional analysis of PRAMS data (2016–2017) from 32 U.S. states  *USA*  n = 57,046  EV n=301  CC n=5063  Dual n=529 | No | Self-reported exclusive vaping, use of combustible cigarettes or dual use:  During the 3 months before pregnancy  During the last 3 months of pregnancy  **Usage frequency**: NR | **LBW:** No significant association with EV group but 57.8g lower birthweight (95%CI −134.3, 18.6) **PTB:** No association with EV group (aRR 1.39 [95%CI 0.84, 2.30]) **SGA:** No association with EV group (aRR 0.78 [95%CI 0.48, 1.27]) | **LBW**: EV had lower risk then CC group who had lower birthweight (−210.3g [95%CI −235.6, −184.9]) and dual use group (−193.9g [95%CI−274.9, −112.8])  **PTB**: EV had lower risk then CC group (aRR 1.28 [95%CI 1.10, 1.47]) no difference to dual use group  **SGA**: EV had lower risk then CC group (aRR 2.30 [95%CI 2.01, 2.63]) and dual use group (aRR 1.93 [95%CI 1.31, 2.83]) |
| 13. **Kim (2020)^(47)^** | Cross-sectional analysis using PRAMS Phase 8 data (2016–2018)  *USA*  n=55,251  EV n=331  CC n=3484 | No | Self-reported exclusive vaping, use of combustible cigarettes or dual use:  During the 3 months before pregnancy  During the last 3 months of pregnancy  **Usage frequency**: NR | **LBW:** EV group had increased risk (OR 1.53, [95%CI 1.06–2.22])  **PTB:** EV group had increased risk (OR 1.86, [95%CI 1.11–3.12])  **SGA:** EV group had increased risk (OR 1.76, [95%CI 1.04–2.96]) | **LBW**: EV no significant difference in risk compared to CC group (aOR 0.71 [95%CI 0.37-1.37])  **PTB**: EV group no significant difference in risk compared to CC group (aOR 1.06 [95%CI 0.46-2.48])  **SGA**: no significant difference in risk compared to CC group (aOR 0.67 [95%CI 0.30-1.47]) |
| 14. **Le (2025)^(48)^** | Prospective preconception cohort study (PRESTO)  USA & Canada  n=6,136 females  EV n=127  CC n=282  Dual n=55 | No | Self-reported vaping and smoking status preconception and in early pregnancy. Defined groups: current vapers/never smokers (EV), current smokers/never vapers (CC), dual users (current vaping + smoking).  **Usage frequency**: current e-liquid volume (mL/day), categorized <3 vs ≥3 mL/day. | **LBW**: NR  **PTB**: NR  **SGA**: NR  **Other:**  **Miscarriage (Spontaneous Abortion - SAB)**  No association with EV group (SAB = 4 aHR 0.64 [95%CI 0.22 - 1.89]) | **LBW**: NR  **PTB**: NR  **SGA**: NR  **Other:**  **Miscarriage (Spontaneous Abortion – SAB)**  Risk with EV equivalent to CC (SAB = 30 aHR 0.79 [95%CI 0.55 - 1.15]) and dual use (SAB = 7 aHR 0.96 [95%CI 0.46 - 2.01]) |
| 15. **Lin (2023)^(49)^** | Retrospective cohort study using U.S. PATH survey data (2013–2018)  *USA*  n=1130  EV n=41  CC n=NR | No | Self-reported exclusive vaping or use of combustible cigarettes any use in pregnancy or quit before pregnancy  **Usage frequency**: NR, use of flavourings and additives reported | **LBW:** included in composite “high-risk birth” outcome  **PTB:** included in composite “high-risk birth” outcome no EV association (aOR 1.19 [95%CI 0.38–3.73]) **SGA:** NR.  **Other:**  **High-risk birth outcome** no EV association for quit before pregnancy (aOR 1.14 [95%CI 0.54-2.40]) or any use in pregnancy (aOR 1.19 [95%CI 0.38–3.73])  **Fetus death** no EV association for quit before pregnancy (aOR 0.39 [95%CI 0.07-2.26]) or any use in pregnancy (aOR 0.33 [95%CI 0.04-2.83])  **Mint/menthol flavour** use significantly associated with higher odds of fetal death (aOR 3.27 [95%CI 1.17–9.19]). | **LBW**: included in composite “high-risk birth” outcome  **PTB**: included in composite “high-risk birth” outcome  **SGA**: NR  **Other**:  **High-risk birth outcome** Lower risk in EV group compared to CC group in quit before pregnancy (aOR 2.69 [95%CI 0.97-7.42]) no difference in any use in pregnancy (aOR 1.52 [95%CI 0.62-3.68])  **Fetus death** no difference in EV and CC groups who either quit before pregnancy (aOR 0.96 [95%CI 0.18-4.91]) or any use in pregnancy (aOR 0.56 [95%CI 0.15-2.15]) |
| 16. **McBride (2021)^(50)^** | Population-based cross-sectional study using U.S. PRAMS data (2016–2018)  *USA*  n=42,827  EV n= 385 | No | Self-reported exclusive vaping, use of combustible cigarettes or dual use:  During the 3 months before pregnancy  During the last 3 months of pregnancy  **Usage frequency**: Usage quantified to <1 day/week, 1-6 days/week, >1 per day. | **LBW:** NR **PTB:** NR **SGA:** NR **Other:**  Reduced rates of **Breastfeeding >3 months** in EV group (OR 0.63 [95% CI: 0.44–0.89]) | **LBW**: NR  **PTB**: NR  **SGA**: NR  **Other**: NR |
| 17. **McDonnell (2020)^(51)^** | Prospective observational cohort study  *Ireland*  n= 425  EV n= 218  CC n=99  Dual n=195 | No | Self-reported use at the time of survey (12-14 weeks), exclusive vaping, use of combustible cigarettes or dual use.  **Usage frequency**: NR | **LBW**: No association with EV group (3470 ± 555 g p=0.97 compared to non-use)  **PTB**: NR **SGA**: No association with EV group (24 [11%] p=0.60 compared to non-use)  **Other**:  **Breastfeeding at discharge rates** lower in EV group (48.6% p=0.03) compared to non-user (61.1%)  **NICU admissions**: No association with EV group (6.9% compared to 4.6% in non-use p=0.42) | **LBW**: EV group had lower risk than CC group (3166 ± 502 g p=< 0.001) and dual use group (3140 ± 628 g p=< 0.001)  **PTB**: NR  **SGA**: EV group had lower risk than CC group (28% p=< 0.001) and dual use group (30.7% p=< 0.001)  **Other**:  **Breastfeeding at discharge rates** EV group had higher rates of breastfeeding than CC group (27.2% p=< 0.001) and dual use group (29.7% p=< 0.001)  **NICU admissions**: no difference in EV and CC group (6% p=0.78) and dual use group (7.6% p=0.75) |
| 18. **Nanninga (2023)^(52)^** | Cross-sectional study  *Netherlands*  n=1937  EV n=10  CC n=209 | No | Self-reported exclusive vaping, use of combustible cigarettes or dual use via postnatal online questionnaire (at any time in pregnancy)  **Usage frequency**: NR | **LBW:** NR   **PTB:** No association with EV group (0%)  **SGA:** EV group had increased risk (20% p=0.02)   **Other:**  **Miscarriage** rates higher in EV group (30% p=0.01)  **Composite adverse maternal outcome**: including HTN, GDM & PPH. No association with EV group (40% p=0.27) | **LBW**: NR  **PTB**: EV group had non-significant reduction in risk (4.3% p=0.75) compared to CC group  **SGA**: EV group had higher risk than CC group (7.2%)  **Other**:  **Miscarriage** EV had higher rates of miscarriage than CC group (5.7%)  **Composite adverse maternal outcome**: non-significant reduction in risk (19.6% p=0.27) compared to CC group |
| 19. **Nian (2025)^(53)^** | Descriptive surveillance analysis using PRAMS data (2016–2020)  *USA*  n=29,505  EV n=117  CC n=14,096  Dual n=278 | No | Self-reported exclusive vaping, use of combustible cigarettes or dual use:  During the 3 months before pregnancy  During the last 3 months of pregnancy  **Usage frequency**: Usage quantified to <1 day/week, 1-6 days/week, >1 per day. | EC initiators, subdivided into EC dual users and EC replacers who quit CCs and commenced EV. Quitters used as control group.  **LBW**: EV group had increased risk (7.5% [95%CI 3.1-14.9])  **PTB**: EV group had increased risk (4.8% [95%CI 2.1-9.2])  **SGA**: EV group had increased risk (5.7% [95%CI 2.2-12]) | EC initiators, subdivided into EC dual users and EC replacers who quit CCs and commenced EV. Quitters used as control group.  **LBW**: EV group had lower risk than CC group (13.3% [95%CI 12.6-14]) and dual use group (11.9% [95%CI 7.6-17.4])  **PTB**: EV group had lower risk than CC group (13% [95%CI 12.2-13.8]) and dual use group (13.1% [95%CI 7.9-19.8])  **SGA**: EV group had lower risk than CC group (19.3% [95%CI 18.2-20.4]) and dual use group (21.3% [95%CI 13.4-31.2]) |
| 20. **Opondo (2021)^(54)^** | Cross-sectional, population-based survey  *England*  n = 4,421  EV n=27 | No | Self-reported exclusive vaping, use of combustible cigarettes or dual use since becoming aware of the pregnancy.  **Usage frequency**: smoking frequency daily, <daily 1/week, less than weekly but at least months, less than monthly | **LBW**: No association with EV group (-31.7g [95%CI -125.8-62.5])  No significant association with exclusive vaping after adjusting for smoking patterns **PTB**: No association with EV group (aRR 1.29 [95%CI 0.54-2.04]) **SGA**: NR  **Other:**  **Lower breastfeeding initiation rates** No association (aRR 1.03 [95%CI 0.98 to 1.08]) | **LBW**: NR  **PTB**: NR  **SGA**: NR  **Other**: NR |
| 21. **Pesola (2024)^(55)^** | Secondary analysis of the PREP RCT  *UK*  n=1,140  EV/NRT n=166  CC n=322  Dual n=582 | Yes  Saliva cotinine and anabasine testing, and exhaled CO | Outcomes for EV and NRT groups combined (as no difference in their individual outcomes). All participants were CC users at baseline  **Usage frequency**: NR | **LBW**: No association (aRR 0.64 [95%CI 0.24–1.72]).  **PTB**: Reduced risk in EV/NRT group (aRR 0.29 [95%CI 0.12–0.70])  **SGA**: NR  **Other**:  **Maternal respiratory outcomes**  Cough NR  Phlegm NR  **NICU admission** No association (aRR 0.57 [95%CI 0.21–1.59]) | **LBW**: Lower risk in EV/NRT group (difference 0.15 kg [95%CI 0.05–0.25])  **PTB**: No significant reduction in risk CC risk 11.2%, dual risk 9.6%.  **SGA**: NR  **Other**:  **Maternal respiratory outcomes**  **Cough** Lower risk than non-EC group (aRR 0.59 [95%CI 0.37–0.93])  **Phlegm** Lower risk than non-EC group (aRR 0.53 [95%CI 0.31–0.92])  **NICU admission** No significant reduction in risk CC risk 6.8%, dual risk 9.6%. |
| 22. **Regan & Bombard (2021)^(56)^** | Retrospective cohort study using PRAMS data (2016–2018)  *USA*  n = 79,176  3 months before pregnancy  EV n=462  CC n=13015  Dual n=1695  Last 3 months of pregnancy  EV n=329  CC n=5100  Dual n=577 | No | Self-reported exclusive vaping, use of combustible cigarettes or dual use:  During the 3 months before pregnancy  During the last 3 months of pregnancy  **Usage frequency**: Usage quantified to <1 day/week, 1-6 days/week, >1 per day. | **LBW:** EV group had increased risk (aPR 1.33 [95%CI 1.06–1.66]) **PTB:** EV group had increased risk (aPR 1.69; [95%CI 1.20–2.39]).  **SGA:** No significant association (aPR 1.10; [95%CI 0.65–1.86]). | **LBW**: EV group had higher risk than dual users (aPR 1.05 [95%CI 0.80–1.38])  **PTB**: EV group had higher risk than dual users (aPR 0.82 [95%CI 0.59–1.14])  **SGA**: EV group had equivalent risk to dual users (aPR 1.15 [95%CI 0.89–1.48]) |
| 23. **Regan & Pereira (2021)^(57)^** | Retrospective cohort study using PRAMS data (2016–2018)  *USA*  N=16,022  Last 3 months of pregnancy  EV n=189  CC n=6310  Dual n=585 | No | Self-reported exclusive vaping, use of combustible cigarettes or dual use:  During the 3 months before pregnancy  During the last 3 months of pregnancy  **Usage frequency**: Usage quantified to <1 day/week, 1-6 days/week, >1 per day. | Quitters used as non-use group  **LBW:** EV group had greater risk when compared to quitters (quitter aPR 0.53, [95%CI 0.47–0.60]) **PTB:** EV group had greater risk when compared to quitters (quitter aPR 0.70, [95%CI 0.61–0.81]) **SGA:** EV group had greater risk when compared to quitters  (quitter aPR 0.46, [95%CI 0.41–0.53]) | CC users used as reference group, no statistical difference in EV risk  **LBW**: EV group had equivalent risk to dual users (aPR 1.12 [95%CI 0.85, 1.46]) and CC group (aPR 0.81, [95% CI 0.54–1.21])  **PTB**: EV group had equivalent risk to Dual users (aPR 0.88 [95%CI 0.64, 1.22]) and CC group (aPR 0.85, [95% CI 0.55–1.31])  **SGA**: EV group had equivalent risk to Dual users (aPR 1.20 [95%CI 0.94, 1.55]) and CC group (aPR 0.56, [95% CI 0.29–1.08]) |
| 24. **Shittu (2022)^(58)^** | Secondary analysis of a nationally representative cohort (PRAMS 2016–2018)  *USA*  n = 5,446,900  EV n= 61,173  CC n=773586  Dual n=149,152 | No | Self-reported exclusive vaping, use of combustible cigarettes or dual use:  During the 3 months before pregnancy  During the last 3 months of pregnancy  **Usage frequency**: Usage quantified to <1 day/week, 1-6 days/week, >1 per day. | **LBW**: NR **PTB**: NR **SGA**: Increased risk with EV group (aRR 1.52 [95%CI 1.45-1.60]).  Quitting EV reduced risk to rates similar to non-users (aRR 0.84 [95%CI 0.82-0.87]) | **LBW**: NR  **PTB**: NR  **SGA**: Lower risk with EV compared to CC use (aRR 2.15 [95%CI 2.14-2.17]) and dual use (aRR 2.09 [95%CI 2.00-2.19]) |
| 25. **Wang (2020)^(59)^** | Retrospective cohort study using PRAMS 2016 data  *USA*  n = 31,973  EV n= 267  CC n=2632  Dual n=265 | No | Self-reported exclusive vaping, use of combustible cigarettes or dual use:  During the 3 months before pregnancy  During the last 3 months of pregnancy  **Usage frequency**: Usage quantified to <1 day/week, 1-6 days/week, >1 per day. | **LBW**: NR **PTB**: No association with EV group (aOR 1.2 [955CI 0.5-2.7])  **SGA**: Increased risk in EV group (aOR 2.4 [95%CI 1.0–5.7]) | **LBW**: NR  **PTB**: Lower risk with EV than CC group (aOR 1.5 [95%CI 1.2-2.0]) no difference to dual use (aOR 1.3 [95%CI 0.8-2.3])  **SGA**: Higher risk with EV than CC group (aOR 2.3 [95%CI 1.8-2.9]) and dual use (aOR 2.3 [95%CI 1.3-4.1]) |
| 26. **Wang (2022)^(60)^** | Cohort study using US PRAMS data (2016–2018)  *USA*  n=99,201  EV n=968  CC n=15,662  Dual n= 2,629 | No | Self-reported exclusive vaping, use of combustible cigarettes or dual use:  During the 3 months before pregnancy  During the last 3 months of pregnancy  **Usage frequency**: Usage quantified to <1 day/week, 1-6 days/week, >1 per day. | **LBW:** NR **PTB:** No association with EV group (aOR 1.21 [95%CI 0.65–2.26]) **SGA:** No association with EV group (aOR 1.14 [95%CI 0.52–2.54]) | **LBW**: NR  **PTB**: Lower risk with EV than CC group (aOR 1.45 [95%CI 1.19-1.76]) no difference to dual use (aOR 1.44 [95%CI 0.78–2.65])  **SGA**: Lower risk with EV than CC group (aOR 2.78 [95%CI 2.34–3.29]) and dual use (aOR 3.08 [95%CI 1.33–7.10]) |
| 27. **Wen & Liu (2023)^(61)^** | Retrospective cohort study using US PRAMS data (2016–2021).  *USA*  n=10,428 EV n=152  CC n=719  Dual n=125 | No | Self-reported exclusive vaping, cigarette use, or dual use during late pregnancy.  **Usage frequency**: Usage quantified to <1 day/week, 1-6 days/week, >1 per day. | **LBW:** NR **PTB:** NR **SGA:** No association with EV group (AOR 1.68, [95%CI 0.89–3.18[) | **LBW:** NR **PTB:** NR **SGA:** EV group had equivalent risk to Dual users (AOR 1.00 [95%CI 0.38-2.62]) and CC group (AOR 1.50 [95%CI 0.74-3.02]) |
| 28. **Wen & Thomas (2023)^(62)^** | Secondary analysis of PRAMS cohort data (2016–2020).  *USA*  n = 176,882  EV n= 921  CC n= 13,697  Dual n= 1,308 | No | Self-reported exclusive vaping, use of combustible cigarettes or dual use:  During the 3 months before pregnancy  During the last 3 months of pregnancy  **Usage frequency**: Usage quantified to <1 day/week, 1-6 days/week, >1 per day. | **LBW:** NR **PTB:** NR **SGA:** NR **Other:**  **Low maternal gestational weight** **gain** No association with EV group (aOR 0.99, [95% CI: 0.78–1.27]). | **LBW**: NR  **PTB**: NR  **SGA**: NR  **Other**:  **Low maternal gestational weight gain** Lower risk with EV group than CC group (aOR 1.26, [95% CI: 1.18–1.35]) no difference with dual use (aOR 1.18, [95% CI: 0.96–1.44]). |
| 29. **Wen & Kwak (2024)^(63)^** | Retrospective cohort using PRAMS (2016–2021).  *USA*  n=194,762  EV n=2,921  CC n=20,255  Dual n=3,700 | No | Self-reported exclusive vapers, exclusive smokers, dual users. Analysed continuation vs cessation during pregnancy.  **Usage frequency**: Usage quantified to <1 day/week, 1-6 days/week, >1 per day. | **LBW:** NR **PTB:** NR **SGA:** NR **Other:**  **PPD**  Increased risk in EV group (13.2% vs 9.7% in non-users) no reduction in risk with EV cessation (12.0% aOR 0.96 [95% CI 0.63-1.45]) | **LBW:** NR **PTB:** NR **SGA:** NR **Other:**  **PPD**  EV group had equivalent risk to CC group (13.4%) cessation reduced CC risk (10.7%; aOR, 0.82, 95% CI 0.71-0.95)  EV group had lower risk than dual group (19.8%) cessation of both reduced dual group risk (11.3%; aOR, 0.59, 95% CI 0.39-0.89) cessation of only vaping (13.7%; aOR, 0.62, 95% CI 0.40-0.98) or only smoking (13.5%; aOR, 0.59, 95% CI 0.31-1.12) reduced risk |
| 30. **Wen & Moe (2024)^(64)^** | Retrospective cohort using PRAMS (2016–2021), linked to birth certificates.  *USA*  n=223,805.  EV n=4,090.  CC n=30,809.  Dual n=6,428; | No | Self-reported EC/CC use before and during pregnancy. Analysed continuation vs cessation during pregnancy. Groups: exclusive vapers, exclusive smokers, dual users.  **Usage frequency**: Usage quantified to <1 day/week, 1-6 days/week, >1 per day. | Comparison between quitters and ongoing use through pregnancy  **LBW:** NR **PTB:** No association with EV group vs quitters (11.0% vs 8.3%; aOR, 0.80 [95% CI, 0.55-1.15]). **SGA:** NR | **LBW:** NR **PTB:** NR  **SGA:** NR |

aOR = adjusted Odds ratio ; aRR = adjusted risk ratio ; CC = combustible cigarettes ; EV = Exclusive vaping; GDM = Gestational diabetes; HTN = Hypertension; NICU = neonatal in tensive care unit; NR = Not reported;

## References:

1.	Aboaziza E, Feaster K, Hare L, Chantler PD, Olfert IM. Maternal electronic cigarette use during pregnancy affects long-term arterial function in offspring. Journal of Applied Physiology. 2023;134(1):59-71.

2.	Alarabi A. Platelet Function and Gene Expression in E-cigarette In Utero Exposed Mice. The Journal of Pharmacology and Experimental Therapeutics. 2023;385:511.

3.	AlHarthi A, Alasmari F, AlSharari SD, Alrasheed NM, Alshammari MA, Alshammari TK. Investigating Behavioral and Neuronal Changes in Adolescent Mice Following Prenatal Exposure to Electronic Cigarette (E-Cigarette) Vapor Containing Nicotine. Brain Sciences. 2023;13(10):1417.

4.	Al-Sawalha N, Alzoubi K, Khabour O, Karaoghlanian N, Ismail Z, Shihadeh A, et al. Effect of electronic cigarette aerosol exposure during gestation and lactation on learning and memory of adult male offspring rats. Physiology & behavior. 2020;221:112911.

5.	Archie SR, Sifat AE, Zhang Y, Villalba H, Sharma S, Nozohouri S, et al. Maternal e-cigarette use can disrupt postnatal blood-brain barrier (BBB) integrity and deteriorates motor, learning and memory function: influence of sex and age. Fluids and Barriers of the CNS. 2023;20(1):17.

6.	Aslaner DM, Alghothani O, Saldana TA, Ezell KG, Yallourakis MD, MacKenzie DM, et al. E-cigarette vapor exposure in utero causes long-term pulmonary effects in offspring. American Journal of Physiology-Lung Cellular and Molecular Physiology. 2022;323(6):L676-L82.

7.	Awada C, Saporito AF, Zelikoff JT, Klein CB. E-cigarette exposure alters neuroinflammation gene and protein expression in a murine model: insights from perinatally exposed offspring and post-birth mothers. Genes. 2024;15(3):322.

8.	Burrage EN, Aboaziza E, Reppert SA, O’Reilly J, Hare LA, Chantler PD, et al. Cerebrovascular Impairment Due to Maternal Electronic Cigarette Usage During Pregnancy is not Dose Dependent. The FASEB Journal. 2020;34(S1):1-.

9.	Burrage E, Aboaziza E, Hare L, Reppert S, Moore J, Goldsmith WT, et al. Long-term cerebrovascular dysfunction in the offspring from maternal electronic cigarette use during pregnancy. American Journal of Physiology-Heart and Circulatory Physiology. 2021;321(2):H339-H52.

10.	Cahill KM, Johnson TK, Perveen Z, Schexnayder M, Xiao R, Heffernan LM, et al. In utero exposures to mint-flavored JUUL aerosol impair lung development and aggravate house dust mite-induced asthma in adult offspring mice. Toxicology. 2022;477:153272.

11.	Cahill KM, Gartia MR, Sahu S, Bergeron SR, Heffernan LM, Paulsen DB, et al. In utero exposure to electronic-cigarette aerosols decreases lung fibrillar collagen content, increases Newtonian resistance and induces sex-specific molecular signatures in neonatal mice. Toxicological research. 2022:1-20.

12.	Chen H, Li G, Chan YL, Chapman DG, Sukjamnong S, Nguyen T, et al. Maternal e-cigarette exposure in mice alters DNA methylation and lung cytokine expression in offspring. American journal of respiratory cell and molecular biology. 2018;58(3):366-77.

13.	Chen H, Li G, Chan YL, Nguyen T, van Reyk D, Saad S, et al. Modulation of neural regulators of energy homeostasis, and of inflammation, in the pups of mice exposed to e-cigarettes. Neuroscience letters. 2018;684:61-6.

14.	Church JS, Chace-Donahue F, Blum JL, Ratner JR, Zelikoff JT, Schwartzer JJ. Neuroinflammatory and behavioral outcomes measured in adult offspring of mice exposed prenatally to e-cigarette aerosols. Environmental Health Perspectives. 2020;128(4):047006.

15.	Kishinchand R, Boyce M, Vyas H, Sewell L, Mohi A, Brengartner L, et al. In Utero Exposure to Maternal Electronic Nicotine Delivery System use Demonstrate Alterations to Craniofacial Development. The Cleft Palate Craniofacial Journal. 2024;61(8):1389-97.

16.	Li G, Chan YL, Wang B, Saad S, George J, Oliver BG, et al. E‐cigarettes damage the liver and alter nutrient metabolism in pregnant mice and their offspring. Annals of the New York Academy of Sciences. 2020;1475(1):64-77.

17.	McAlinden KD, Chan YL, Kota A, Chen H, Oliver BG, Sharma P. Maternal E-cigarette vaping enhances development of allergic asthma in the offspring. D98 Insights into Environmental Exposures in Asthma, Copd, and Constrictive Bronchiolitis: American Thoracic Society; 2017. p. A7333-A.

18.	Mills A, Nassabeh S, Frazier J, Robinson M, Legg P, Shamma A, et al. Maternal Vaping during Pregnancy E-cigs Leads to Obesity in Offspring. Physiology. 2023;38(S1):5729326.

19.	Mills A, Corbin D, Dakhallah D, Chantler PD, Olfert IM. Nicotine Influence on Vascular and Neurocognitive Function with In-utero Electronic Cigarette Exposure. bioRxiv. 2025.

20.	Mills A, Dakhlallah D, Ranpara A, Goldsmith WT, Chantler PD, Huang Y-W, et al. Pregnancy and postpartum effects of electronic cigarettes on maternal health and vascular function in the fourth trimester. Cardiovascular toxicology. 2025;25(3):325-40.

21.	Nassabeh SA. Vaping with Nicotine during Pregnancy Affects Aortic Structure and Function in Rat Offspring: West Virginia University; 2023.

22.	Nguyen T, Li GE, Chen H, Cranfield CG, McGrath KC, Gorrie CA. Maternal e-cigarette exposure results in cognitive and epigenetic alterations in offspring in a mouse model. Chemical research in toxicology. 2018;31(7):601-11.

23.	Noël A, Hansen S, Zaman A, Perveen Z, Pinkston R, Hossain E, et al. In utero exposures to electronic-cigarette aerosols impair the Wnt signaling during mouse lung development. American Journal of Physiology-Lung Cellular and Molecular Physiology. 2020;318(4):L705-L22.

24.	Noël A, Yilmaz S, Farrow T, Schexnayder M, Eickelberg O, Jelesijevic T. Sex-specific alterations of the lung transcriptome at birth in mouse offspring prenatally exposed to vanilla-flavored E-cigarette aerosols and enhanced susceptibility to asthma. International Journal of Environmental Research and Public Health. 2023;20(4):3710.

25.	Orzabal MR, Lunde-Young ER, Ramirez JI, Howe SY, Naik VD, Lee J, et al. Chronic exposure to e-cig aerosols during early development causes vascular dysfunction and offspring growth deficits. Translational Research. 2019;207:70-82.

26.	Orzabal MR, Naik VD, Lee J, Wu G, Ramadoss J. Impact of gestational electronic cigarette vaping on amino acid signature profile in the pregnant mother and the fetus. Metabolism Open. 2021;11:100107.

27.	Orzabal MR, Naik VD, Lee J, Hillhouse AE, Brashear WA, Threadgill DW, et al. Impact of E-cig aerosol vaping on fetal and neonatal respiratory development and function. Translational Research. 2022;246:102-14.

28.	Ozekin YH, Saal ML, Pineda RH, Moehn K, Ordonez-Erives MA, Figueroa MFD, et al. Intrauterine exposure to nicotine through maternal vaping disrupts embryonic lung and skeletal development via the Kcnj2 potassium channel. Developmental biology. 2023;501:111-23.

29.	Reynolds BR, Raph S, Ramalingam A, Srivastava S, Lorkiewicz P, Watson LE, et al. Don’t Go Vaping My Heart: E-cigarette Exposure During Pregnancy Promotes Peripartum Ventricular Arrhythmias and Sympathetic Dominance. American Journal of Physiology-Heart and Circulatory Physiology. 2025.

30.	Richlak E, Shope L, Leonard E, Sewell L, Maykovich T, Mohi A, et al. In utero exposure to electronic cigarette carriers alters craniofacial morphology. PloS one. 2025;20(6):e0327190.

31.	Sifat AE, Nozohouri S, Villalba H, Al Shoyaib A, Vaidya B, Karamyan VT, et al. Prenatal electronic cigarette exposure decreases brain glucose utilization and worsens outcome in offspring hypoxic–ischemic brain injury. Journal of Neurochemistry. 2020;153(1):63-79.

32.	Walayat A, Li Y, Zhang Y, Fu Y, Liu B, Shao XM, et al. Fetal e-cigarette exposure programs a neonatal brain hypoxic-ischemic sensitive phenotype via altering DNA methylation patterns and autophagy signaling pathway. American Journal of Physiology-Regulatory, Integrative and Comparative Physiology. 2021;321(5):R791-R801.

33.	Wang Q, Sundar IK, Blum JL, Ratner JR, Lucas JH, Chuang T-D, et al. Prenatal exposure to electronic-cigarette aerosols leads to sex-dependent pulmonary extracellular-matrix remodeling and myogenesis in offspring mice. American journal of respiratory cell and molecular biology. 2020;63(6):794-805.

34.	Yu C, Liu J, Sakurai R, Wang Y, Afrose L, Gour A, et al. Perinatal nicotine vaping exposure induces pro-myofibroblastic phenotype in rat bone marrow-derived mesenchymal stem cells. Reproductive Toxicology. 2024;129:108673.

35.	Ammar L, Tindle HA, Miller AM, Adgent MA, Nian H, Ryckman KK, et al. Electronic cigarette use during pregnancy and the risk of adverse birth outcomes: A cross-sectional surveillance study of the US Pregnancy Risk Assessment Monitoring System (PRAMS) population. PloS one. 2023;18(10):e0287348.

36.	Ashford K, McCubbin A, Barnett J, Blair LM, Lei F, Bush H, et al. Longitudinal examination of prenatal tobacco switching behaviors and birth outcomes, including Electronic Nicotine Delivery System (ENDS) and dual use. Maternal and child health journal. 2021;25(8):1175-81.

37.	Azar M, Oatey ME, Moniz MH, Bailey BA. Intrapartum Electronic Cigarette Use and Birth Outcomes: Evidence from a Population-Based Study. International Journal of Environmental Research and Public Health. 2024;21(11):1449.

38.	Bailey BA, Azar M, Nadolski K, Dodge P. Fetal Growth Following Electronic Cigarette Use in Pregnancy. International Journal of Environmental Research and Public Health. 2024;21(9):1179.

39.	Cardenas VM, Cen R, Clemens MM, Moody HL, Ekanem US, Policherla A, et al. Use of Electronic Nicotine Delivery Systems (ENDS) by pregnant women I: Risk of small-for-gestational-age birth. Tobacco induced diseases. 2019;17:44.

40.	Cardenas VM, Ali MM, Fischbach LA, Nembhard WN. Dual use of cigarettes and electronic nicotine delivery systems during pregnancy and the risk of small for gestational age neonates. Annals of epidemiology. 2020;52:86-92. e2.

41.	Choi BM, Weinberger AH, Petersen N, Pang RD, DeVito EE, Bell ML, et al. Association of e-cigarette use and postpartum depression: pregnancy risk assessment monitoring system 2016–2019. Journal of Women's Health. 2024;33(1):45-51.

42.	Cui Y, Visser A, Tsaih S-W, Palatnik A. Adverse birth outcomes associated with use of nicotine products during pregnancy. American Journal of Obstetrics & Gynecology. 2022;226(1):S148.

43.	Froggatt S, Reissland N, Covey J. The effects of prenatal cigarette and e-cigarette exposure on infant neurobehaviour: A comparison to a control group. EClinicalMedicine. 2020; 28: 100602. 2020.

44.	Froggatt S, Reissland N, Covey J, Kumarendran K. Foetal mouth movements: Effects of nicotine. Acta paediatrica. 2021;110(11):3014-20.

45.	Galbo A, Izhakoff N, Courington C, Castro G, Lozano J, Ruiz-Pelaez J. The association between electronic cigarette use during pregnancy and unfavorable birth outcomes. Cureus. 2022;14(7).

46.	Hawkins SS, Wylie BJ, Hacker MR. Associations between electronic nicotine delivery systems and birth outcomes. The journal of maternal-fetal & neonatal medicine. 2022;35(25):6868-75.

47.	Kim S, Oancea SC. Electronic cigarettes may not be a “safer alternative” of conventional cigarettes during pregnancy: evidence from the nationally representative PRAMS data. BMC pregnancy and childbirth. 2020;20:1-9.

48.	Le AD, Zhang CA, Chen AL, Basran S, Seranio N, Scott M, et al. A Preconception Cohort Study of Nicotine Vaping and Incidence of Spontaneous Abortion. Nicotine and Tobacco Research. 2025:ntaf141.

49.	Lin S-y, Wang L, Zhou W, Kitsantas P, Wen X, Xue H. E-cigarette use during pregnancy and its association with adverse birth outcomes in the US. Preventive medicine. 2023;166:107375.

50.	McBride M, Haile ZT. Association between electronic nicotine delivery systems use and breastfeeding duration. Breastfeeding Medicine. 2021;16(11):886-93.

51.	McDonnell B, Dicker P, Regan C. Electronic cigarettes and obstetric outcomes: a prospective observational study. BJOG: An International Journal of Obstetrics & Gynaecology. 2020;127(6):750-6.

52.	Nanninga EK, Weiland S, Berger MY, Feijen-de Jong EI, Erwich JJH, Peters LL. Adverse maternal and infant outcomes of women who differ in smoking status: E-cigarette and tobacco cigarette users. International Journal of Environmental Research and Public Health. 2023;20(3):2632.

53.	Nian H, Odland R, Mindlin S, Ammar L, Tindle H, Miller AM, et al. Demographic characteristics, perinatal smoking patterns, and risk for neonatal health complications among pregnant smokers in the United States who begin using electronic cigarettes during pregnancy: a descriptive study using population-based surveillance data. Nicotine and Tobacco Research. 2024;26(11):1455-62.

54.	Opondo C, Harrison S, Alderdice F, Carson C, Quigley MA. Electronic cigarette use (vaping) and patterns of tobacco cigarette smoking in pregnancy–evidence from a population-based maternity survey in England. PloS one. 2021;16(6):e0252817.

55.	Pesola F, Smith KM, Phillips‐Waller A, Przulj D, Griffiths C, Walton R, et al. Safety of e‐cigarettes and nicotine patches as stop‐smoking aids in pregnancy: Secondary analysis of the Pregnancy Trial of E‐cigarettes and Patches (PREP) randomized controlled trial. Addiction. 2024;119(5):875-84.

56.	Regan AK, Bombard JM, O’Hegarty MM, Smith RA, Tong VT. Adverse birth outcomes associated with prepregnancy and prenatal electronic cigarette use. Obstetrics & Gynecology. 2021;138(1):85-94.

57.	Regan AK, Pereira G. Patterns of combustible and electronic cigarette use during pregnancy and associated pregnancy outcomes. Scientific reports. 2021;11(1):13508.

58.	Shittu AAT, Kumar BP, Okafor U, Berkelhamer SK, Goniewicz ML, Wen X. Changes in e-cigarette and cigarette use during pregnancy and their association with small-for-gestational-age birth. American journal of obstetrics and gynecology. 2022;226(5):730. e1-. e10.

59.	Wang X, Lee NL, Burstyn I. Smoking and use of electronic cigarettes (vaping) in relation to preterm birth and small-for-gestational-age in a 2016 US national sample. Preventive medicine. 2020;134:106041.

60.	Wang X, Lee NL, Burstyn I. Exposure-response analysis of the association of maternal smoking and use of electronic cigarettes (vaping) in relation to preterm birth and small-for-gestational-age in a national US sample, 2016–2018. Global Epidemiology. 2022;4:100079.

61.	Wen X, Liu L, Moe AA, Ormond IK, Shuren CC, Scott IYN, et al. Use of e-cigarettes and cigarettes during late pregnancy among adolescents. JAMA Network Open. 2023;6(12):e2347407-e.

62.	Wen X, Thomas MA, Liu L, Moe AA, Duong PH, Griffiths ME, et al. Association between maternal e‐cigarette use during pregnancy and low gestational weight gain. International Journal of Gynecology & Obstetrics. 2023;162(1):300-8.

63.	Wen X, Liu L, Kwak N, Chaudhuri P, Panzica H. CHANGE IN E-CIGARETTE AND CIGARETTE USE DURING PREGNANCY AND POSTPARTUM DEPRESSION. Chest. 2024;166(4):A6547.

64.	Wen X, Moe A, Patel D, Hastings G, Manney K. CHANGE IN MATERNAL E-CIGARETTE USE DURING PREGNANCY AND PRETERM BIRTH. Chest. 2024;166(4):A6546.
