# Supplementary material for: Vaping during pregnancy and maternal, fetal, and infant health outcomes: A scoping review of human and animal studies
Source: PLoS One. 2026 Sep 29;21(9):e0356305. doi: 10.1371/journal.pone.0356305 (PMC13623096; doi:10.1371/journal.pone.0356305)
Supplement: Supplementary file 4 [file pone.0356305.s004.docx]

# S4 Appendix. Structured study appraisal and detailed exposure classification

This table contains a basic quality stratification of included human studies and a detailed exposure classification.

# Table S1: Human studies:

| **Author and Year** | **Design and data source** | **Exposure timing** | **Exposure group definitions – EV, dual use and non-use** | **Bio-chemical Validation** | **Previous smoking or dual use addressed** | **Residual confounding from prior smoking** | **Confounder adjustment** | **Main interpretability limitation** | **Interpretability** |
| --- | --- | --- | --- | --- | --- | --- | --- | --- | --- |
| 1. **Ammar (2023)^(1)^** | Cross-sectional surveillance study using US PRAMS data  *USA*  n=190,707  EV n=206  CC n=14752  Dual n=1404 | PRAMS self-report for 3 months before pregnancy and last 3 months of pregnancy. Primary exposure groups based on last 3 months of pregnancy. Quitting analysis based on pre-pregnancy EC use and continuation/cessation during pregnancy. | **EV:** self-reported EC-only use in the last 3 months of pregnancy.  **Dual use:** self-reported EC and CC use in the last 3 months of pregnancy.  **Non-use:** no self-reported EC or CC use in the last 3 months of pregnancy.  **Smoking history:** CC use also captured for the 3 months before pregnancy | No | Partial. Dual use was identified, but smoking history and exposure status relied on retrospective self-report | Partially addressed through pre-pregnancy CC data and adjustment for CC quantity in quitting analysis. Residual confounding remains possible due to retrospective self-report, no biochemical validation and potential earlier smoking/switching among late-pregnancy EC-only users. | Adjusted for maternal sociodemographic, behavioral and obstetric factors including age, race/ethnicity, education, marital status, income, WIC, pregnancy intention, prenatal care, parity, prior preterm birth, BMI, multivitamin use, alcohol, residency and year. EC quitting analysis additionally adjusted for CC quantity before and during pregnancy. | PRAMS-based retrospective self-report, no biochemical validation, potential exposure misclassification and overlap with other PRAMS analyses. | Moderate to lower |
| 2. **Ashford (2021)^(2)^** | Prospective longitudinal cohort study.  *Kentucky, USA*  n=218  EV n=21  CC n=106  Dual n=91  Non-use/quitters n=25 | Prospective trimester-specific assessment. Participants recruited in first or second trimester and followed longitudinally, with tobacco product use assessed at each trimester study visit. | **EV:** self-reported ENDS-only use at trimester study visits.  **Dual use:** self-reported conventional cigarette and ENDS use.  **Non-use:** quitters used as comparator, defined as previous conventional-only, ENDS-only or dual users who later reported no product use. Not a never-user comparator, as one participant who remained non-smoking throughout pregnancy was excluded.  **Smoking history:** all participants were tobacco/nicotine users at enrolment, and first-trimester smoking behaviour was included in the analysis. | Yes – urinary and serum cotinine | Yes/partial. Switching behavior and dual use were specifically examined. | Prospective trimester-specific assessment. Participants recruited in first or second trimester and followed longitudinally, with tobacco product use assessed at each trimester study visit. | Potential confounders assessed included age, prior preterm birth, first-trimester smoking behavior, race, marital status, education, income and employment. Multivariable models controlled for age, prior preterm birth and first-trimester smoking behavior, with an interaction between switching behavior and first-trimester smoking behavior. | Stronger exposure validation, but small exclusive vaping group and quitters used as comparator rather than never-users. | Higher |
| 3. **Azar (2024)^(3)^** | Retrospective cohort study. Statewide collaborative of 70 maternity hospitals in Michigan (OBI registry). Jan 2021–Jul 2022.  *USA*  n=29,103  EV n=493  CC n=2,216 | Exposure recorded as EC or CC use at any point during pregnancy. The dataset did not provide granular timing, trimester-specific exposure, frequency, duration or cessation/switching patterns. | **EV:** individuals documented in the OBI registry as using e-cigarettes or other vaping products at any point during pregnancy, without documented combustible cigarette use.  **Dual use:** participants using both EC and CC were identified but excluded from the analysis.  **Non-use:** participants definitively identified as non-EC and non-CC users.  **Smoking history:** CC use during pregnancy was identified, but prior smoking history, switching and cessation before pregnancy were not captured in detail. | No | Partial. Dual users were excluded, but previous smoking history may not be fully captured. | Partially addressed by excluding dual users and comparing EC-only, CC-only and non-use groups, but residual confounding remains possible because previous smoking, cessation or switching before/during pregnancy were not measured in detail. EC-only users may include former smokers or intermittent smokers not captured in the registry. No biochemical validation was used, and frequency, duration and timing of EC use were unavailable. | Regression analyses controlled for background factors significantly associated with exposure group. Available covariates included maternal age, race/ethnicity, pre-pregnancy and delivery weight/BMI, gravidity, parity, diabetes, hypertension, marijuana use and Social Vulnerability Index. Participants with alcohol or opioid use were excluded. | Retrospective registry design, self-reported exposure and no biochemical validation | Moderate |
| 4. **Bailey (2024)^(4)^** | Retrospective cohort study, single site  *USA*  n=191  EV n=20 | Retrospective chart review. EV exposure was defined as use at any point during pregnancy or at delivery. All EV participants reported use from the beginning of pregnancy to at least the second-trimester ultrasound, with regular use at the time of the second-trimester scan. Amount, exact timing, type, frequency and duration of e-cigarette use were not consistently available. | **EV:** self-reported electronic cigarette use at any point during pregnancy or at delivery. All EV participants reported use from the beginning of pregnancy through at least the second-trimester ultrasound, and regular use at the time of the second-trimester scan.  **Dual use:** not included. Participants with self-reported or biochemically confirmed combustible tobacco, alcohol or other illicit drug use were excluded.  **Non-use:** control group had no positive indicator for electronic cigarette use and no self-reported or biochemically confirmed tobacco, alcohol or other illicit drug use.  **Smoking history:** combustible cigarette use during pregnancy was excluded, but prior smoking history before pregnancy was not clearly characterised. | No  Urine drug screening used to exclude other substance use. | Partial. Combustible cigarette and other substance use excluded, but exposure remains self-reported | Partially addressed by excluding participants with self-reported or biochemically confirmed combustible tobacco use and other substance use during pregnancy. However, residual confounding remains possible because e-cigarette exposure was based on self-report, biochemical testing did not validate vaping itself, substance use may be under-reported in medical records, and prior smoking or pre-pregnancy switching from cigarettes to vaping was not clearly captured. | Controlled analyses adjusted for background factors associated with group membership at p<0.10. These were race/ethnicity, marital status, education and medical insurance. Other collected variables included parity, pre-pregnancy BMI, pregnancy weight gain, gestational age at first prenatal visit, gestational diabetes, hypertension, gestational age at ultrasound and infant sex, but only significant background differences were entered into the final models | Small exclusive vaping group and no biochemical validation of vaping/nicotine exposure | Moderate to lower |
| 5. **Cardenas (2019)^(5)^** | Prospective cohort study.  *USA*  n=199  EV n=6  CC n=56  Dual n=17 | Current ENDS and cigarette use were defined as use within the previous month at baseline enrolment during pregnancy. Some participants enrolled before 20 weeks and others at or after 20 weeks. Cigarette use in the 3 months before pregnancy was collected only in a subset. Repeated exposure measurement across pregnancy was not available. | **EV:** current ENDS-only use, defined as ENDS use within the previous month without current cigarette smoking.  **Dual use:** current ENDS use with concurrent current cigarette smoking.  **Non-use:** non-current smokers and non-current ENDS users with no reported exposure to second-hand smoke, ENDS aerosols or other tobacco products; biomarker-positive undisclosed tobacco users were excluded from the referent group in sensitivity analyses.  **Smoking history:** ever smoking and current smoking were recorded; number of cigarettes smoked in the 3 months before pregnancy was available for a subset. | Yes  Salivary cotinine, CO, hair nicotine | Yes/partial. Dual use was identified and biomarkers were used | Partly addressed through self-reported smoking history, classification of dual users, and use of biomarkers to identify undisclosed tobacco use in the referent group. Residual confounding remains possible because cigarette quantity in the 3 months before pregnancy was only available for a subset, ENDS/smoking dynamics during pregnancy were not measured in detail, and potential confounders such as BMI, comorbidities, parental anthropometry and paternal factors were not adjusted for. | Models controlled for maternal age and race/ethnicity, identified as actual confounders. Maternal education and parity were considered but not included in final adjusted estimates. Sensitivity analyses corrected for exposure misclassification using salivary cotinine, exhaled CO and hair nicotine. | Strong biochemical validation, but very small exclusive vaping group | Higher |
| 6. **Cardenas (2020)^(6)^** | Population-based retrospective cohort study using 2016–2017 Arkansas PRAMS data  *USA*  n = 1,594 (weighted = 64,981 births)  EV n=18  CC n=372  Dual n=100 | Exposure was assessed retrospectively for the 3 months before pregnancy and the last 3 months of pregnancy using Arkansas PRAMS. Main exposure categories included any tobacco use during either period. Change analyses examined whether cigarette-only and dual users stopped, reduced, or continued smoking between the pre-pregnancy and late-pregnancy periods | **EV:** self-reported ENDS-only use during the 3 months before pregnancy and/or the last 3 months of pregnancy, without cigarette smoking.  **Dual use:** self-reported use of both cigarettes and ENDS during the 3 months before pregnancy and/or the last 3 months of pregnancy.  **Non-use:** self-reported non-use of any tobacco product.  **Smoking history:** cigarette smoking and ENDS use were captured for both the 3 months before pregnancy and the last 3 months of pregnancy, allowing classification of cessation, reduction and continued use. No women reported switching from cigarette-only use to ENDS-only use. | Yes  Hair nicotine and plasma cotinine used in sensitivity analyses | Yes/partial. Dual use and smoking cessation patterns were considered. | Partly addressed through separate classification of cigarette-only, ENDS-only, dual use and non-use, and through analysis of changes in cigarette smoking between pre-pregnancy and late pregnancy. However, residual confounding remains possible because exposure was self-reported postpartum, there was no individual biochemical validation within the PRAMS dataset, ENDS-only numbers were very small, second-hand smoke and other substance use were not assessed, and poly-use with other tobacco products may have biased results. Misclassification correction suggested stronger associations than self-report alone. | Adjusted for maternal age, race/ethnicity, maternal education and prenatal alcohol use, selected using a directed acyclic graph as the minimum sufficient adjustment set. Additional potential confounders considered included parity, marital status, household income, prenatal care adequacy, payment source, WIC, pre-pregnancy BMI, gestational weight gain, pre-pregnancy diabetes, pre-pregnancy hypertension and alcohol use. Sensitivity analyses corrected for exposure misclassification using estimates from hair nicotine and serum cotinine validation studies. | PRAMS-based self-report with very small exclusive vaping group, although biomarker sensitivity analyses strengthen interpretation. | Moderate |
| 7. **Choi (2024)^(7)^** | Cross-sectional analysis of national PRAMS data (2016–2019)  *USA*  n = 58,950 (weighted)  EV 3 months before pregnancy n= 3,879  EV Last 3 months of pregnancy n= 3,834 | PRAMS survey completed 2–6 months postpartum. E-cigarette exposure assessed retrospectively for three windows: past 2 years before survey completion, 3 months before pregnancy, and last 3 months of pregnancy. Main analysis used past 2-year exposure; sensitivity analyses used pre-pregnancy and late-pregnancy exposure windows. | **EV:** self-reported any e-cigarette use, assessed in three ways: any use in the past 2 years before survey completion, any use in the 3 months before pregnancy, and any use in the last 3 months of pregnancy.  **Dual use:** not defined as a separate exposure group, but combustible cigarette and/or hookah use was included as a covariate at corresponding time points.  **Non-use:** self-reported no e-cigarette use for the relevant exposure window. Sensitivity analysis also examined e-cigarette use among participants reporting no combustible cigarette or hookah use.  **Smoking history:** combustible cigarette and/or hookah use was captured for the past 2 years, 3 months before pregnancy, and last 3 months of pregnancy, depending on the model. | No | Partial. Dual use identified, but exposure based on retrospective self-report. | Partly addressed by adjusting for combustible cigarette and/or hookah use at matched time points and by conducting a sensitivity analysis excluding combustible cigarette/hookah users. Residual confounding remains possible because exposure was self-reported, no biochemical validation was used, other nicotine/tobacco products were not captured, e-cigarette frequency/device/nicotine content were not fully characterized, and previous smoking or switching patterns were not directly modelled as exposure categories. | Logistic regression adjusted for age, race, ethnicity, prenatal care in the last trimester, health insurance during pregnancy, physical abuse during pregnancy, annual income, survey response method and combustible cigarette and/or hookah use at the corresponding exposure time point. Sensitivity analyses examined pre-pregnancy use, use in the last 3 months of pregnancy, frequency of use, and e-cigarette use after excluding combustible cigarette/hookah users. | PRAMS-based retrospective self-report, no biochemical validation and outcome based on postpartum depression rather than birth outcome | Moderate to lower |
| 8. **Cui (2022)^(8)^** | Retrospective cohort study using PRAMS data (2012–2018)  *USA*  n=239,292  EV n=357  CC n=24853  Dual n=688 | E-cigarette and combustible cigarette use were defined as use in the last 3 months of pregnancy. Hookah use was defined as exposure within 2 years of pregnancy. No trimester-specific trajectory or pre-pregnancy smoking/vaping transition data were reported. | **EV:** exclusive e-cigarette use in the last 3 months of pregnancy.  **Dual use:** concomitant use of e-cigarettes with combustible cigarettes, hookah, or both; groups included c-cigarette and e-cigarette, e-cigarette and hookah, and all three forms.  **Non-use:** implied PRAMS comparator group with no reported use of the nicotine products assessed.  **Smoking history:** combustible cigarette use was captured as use in the last 3 months of pregnancy; previous smoking or switching before pregnancy was not described in the abstract. | No | Partial. Dual use identified, but previous smoking and exposure history rely on PRAMS self-report. | Partially addressed by separating exclusive e-cigarette use from combustible cigarette use and dual/poly-use categories. However, residual confounding remains possible because exposure was self-reported, no biochemical validation was reported, prior smoking history and switching from combustible cigarettes to e-cigarettes were not described, and the abstract does not specify whether socioeconomic factors, previous smoking, alcohol or other substance use were adjusted for. | Multivariable logistic regression was used. The abstract states that groups differed by maternal age, parity, race/ethnicity, BMI, obstetric risk factors and adequacy of prenatal care, but does not specify the exact covariates included in the adjusted model. | PRAMS-based retrospective self-report, no biochemical validation and potential overlap with other PRAMS analyses. | Moderate to lower |
| 9. **Froggatt (2020)^(9)^** | Case-control study.  *UK*  n=83 infants  EV n=10  CC n=29 | Exposure status was assessed at 32 weeks’ gestation. Infant outcomes were assessed at approximately 1 month postnatal age using the NBAS. No repeated pregnancy exposure assessment or detailed pre-pregnancy-to-pregnancy switching trajectory was available. | **EV:** self-reported maternal e-cigarette use at 32 weeks’ gestation, with nicotine concentration in the product reported as 3–16 mg. **Dual use:** not included as a separate group; infants were classified as exposed to either cigarettes, e-cigarettes or neither. **Non-use:** infants not exposed prenatally to cigarettes or e-cigarettes. **Smoking history:** years of maternal cigarette smoking before conception were recorded and correlated with outcomes, but previous smoking among EV users was not fully separated as an exposure group. | No | Limited. Dual use and previous smoking history unclear from extracted data. | Partly addressed by recording years of smoking prior to conception and additional household smokers, and by using CO breath testing to confirm cigarette smoking status. However, residual confounding remains possible because e-cigarette exposure was self-reported, cotinine was not measured, socioeconomic status was not assessed, prior smoking history among EV users may influence neurobehavioural outcomes, and dual use was not separately classified. | Potential covariates assessed included maternal age, infant sex, primiparity, additional household smokers, prenatal stress, depression and anxiety. Covariates significantly correlated with outcomes were included in ANCOVA where appropriate. Maternal depression was controlled for reflexes; maternal age and depression were controlled for motor maturity. | Small sample, no biochemical validation and limited exposure detail | Lower |
| 10. **Froggatt (2021)^(10)^** | Prospective observational cohort.  *UK*  n = 123 women  EV n=15  CC n= 13 | Exposure was assessed in late pregnancy at the time of 4D ultrasound scans performed at 32 and 36 weeks’ gestation. E-cigarette nicotine concentration and cigarette consumption were obtained by maternal questionnaire, and CO was measured at the scan | **EV:** self-reported maternal e-cigarette use, with reported nicotine concentration 3–16 mg.  **Dual use:** none of the mothers reported dual use of e-cigarettes and cigarettes.  **Non-use:** pregnant women not exposed to cigarettes or e-cigarettes.  **Smoking comparator groups:** light cigarette smoking was defined as <10 cigarettes/day and heavy cigarette smoking as 11–20 cigarettes/day. Smoking history before pregnancy was not clearly separated within the EV group. | Yes  Exhaled Carbon monoxide for cigarette exposure only | Partial. Smoking exposure categorised, but biochemical validation of vaping was limited. | Partly addressed by separating current cigarette smokers from e-cigarette users, recording maternal CO using a Bedfont Smokerlyzer at the scan, and reporting that no mothers reported dual use. However, residual confounding remains possible because EV exposure was self-reported, nicotine dose was based on reported e-liquid concentration rather than actual use, cotinine was not measured, prior smoking history among EV users was not clearly accounted for, and socioeconomic factors, caffeine intake and fasting were not assessed. | Potential confounders measured included maternal stress, anxiety, depression, antenatal attachment, time of day of scan and maternal CO. Time of day was associated with mouth movement frequency at 32 weeks and depression was associated at 36 weeks. Because data did not meet ANCOVA assumptions, correlated covariates were handled using regression residuals followed by Kruskal–Wallis testing. | Specialist prospective outcome data, but small groups and incomplete biochemical validation for vaping. | Moderate |
| 11. **Galbo (2022)^(11)^** | Retrospective cohort study using PRAMS 2016–2017 data.  *USA*  N=71,940 women  EV n= 859 | Exposure was assessed as self-reported e-cigarette or electronic nicotine product use during the last 3 months of pregnancy. | **EV:** self-reported use of e-cigarettes or other electronic nicotine products during the last 3 months of pregnancy, based on PRAMS Phase 8 Q25. The EV group included 859 participants, representing 1.2% of the final sample.  **Dual use:** not separately defined or analysed. The authors state that traditional tobacco use was a potential confounder but could not be measured because it was not addressed in the core questionnaire used for this analysis.  **Non-use:** women who did not report e-cigarette use during pregnancy. The comparison group therefore appears to be “no e-cigarette use” rather than confirmed non-use of all nicotine/tobacco products.  **Smoking comparator groups:** no separate combustible cigarette, dual-use, or former-smoking comparator groups were included. | No | Partial. Dual use identified, but previous smoking and exposure history rely on retrospective self-report. | Poorly addressed. Residual confounding from prior and current combustible cigarette use is a major limitation because traditional tobacco use, alcohol use and drug use were not measured or adjusted for in the core PRAMS questionnaire used. Former smoking status was not reported, dual use was not separated, and the comparison group may include smokers or former smokers. Exposure was also self-reported, with no biochemical validation. | Adjusted for age, race, Hispanic ethnicity, insurance, maternal education, Kotelchuck index/prenatal care, physical abuse during pregnancy, and pregnancy complications including gestational diabetes, high blood pressure and clinical depression. | PRAMS-based retrospective self-report, no biochemical validation and composite outcome limits specificity. | Moderate to lower |
| 12. **Hawkins (2022)^(12)^** | Cross-sectional analysis of PRAMS data (2016–2017) from 32 U.S. states  *USA*  n = 57,046  EV n=301  CC n=5063  Dual n=529 | Exposure was assessed during the last 3 months of pregnancy. Respondents reported whether they used ENDS and whether they smoked cigarettes during this period, and these responses were used to construct the four-category exposure variable. | **EV:** ENDS-only use was defined as any self-reported use of e-cigarettes or other electronic nicotine products during the last 3 months of pregnancy, with no reported cigarette smoking during the same period.  **Dual use:** defined as any ENDS use plus any cigarette smoking during the last 3 months of pregnancy.  **Non-use:** defined as no reported ENDS use and no reported cigarette smoking during the last 3 months of pregnancy.  **Smoking comparator groups:** cigarette-only use was defined as any cigarette smoking during the last 3 months of pregnancy, with no ENDS use. | No | Partial. Dual use identified, but exposure based on retrospective self-report. | Partly addressed. This study improves exposure classification by creating four mutually exclusive groups: non-use, ENDS-only, dual use and cigarette-only use. This reduces confounding from concurrent smoking. However, residual confounding from prior smoking remains possible because former smoking history before the last 3 months of pregnancy was not incorporated into the exposure categories. Exposure was self-reported, with no biochemical validation, and nicotine exposure could be misclassified due to variation in ENDS device type, e-liquid nicotine content and frequency of use. | Adjusted for race/ethnicity, education, maternal age, household income, payment method, WIC use during pregnancy, marital status, timing of first prenatal care visit, language preference, plurality and parity. | PRAMS-based retrospective self-report, no biochemical validation and potential dataset overlap | Moderate to lower |
| 13. **Kim (2020)^(13)^** | Cross-sectional analysis using PRAMS Phase 8 data (2016–2018)  *USA*  n=55,251  EV n=331  CC n=3484 | Exposure was assessed during the last 3 months of pregnancy. Conventional cigarette smoking was available for all trimesters, but EC use was only collected for the third trimester. | **EV:** exclusive electronic cigarette use was defined as self-reported EC use during the third trimester, with no reported conventional cigarette smoking during the same period. EC use included e-cigarettes and other electronic nicotine products, and was dichotomised as any use versus no use.  **Dual use:** defined as both EC use and conventional cigarette smoking during the third trimester. However, dual users were excluded from the final analytical comparisons because the authors considered this group heterogeneous and difficult to interpret.  **Non-use:** complete abstainers were defined as women who reported neither EC use nor conventional cigarette smoking during the third trimester.  **Smoking comparator groups:** exclusive conventional cigarette smokers were defined as women who reported smoking cigarettes but no EC use during the third trimester. | No | Partial. Dual use identified, but exposure based on retrospective self-report. | Partly addressed. This study is stronger than many PRAMS analyses because it separates exclusive EC use from exclusive cigarette smoking, excludes dual users, and matches on first- and second-trimester cigarette smoking. This helps reduce confounding from earlier pregnancy smoking. However, residual confounding remains possible because EC use in the first and second trimester was not collected, pre-pregnancy smoking history was not fully captured, and exposure was self-reported without biochemical validation. Nicotine dose, device type, e-liquid composition and frequency/intensity of use were also not adequately characterised. | Used exact propensity matching rather than conventional covariate adjustment. EC users were matched to complete abstainers and to conventional cigarette smokers using seven covariates: race/ethnicity, maternal age, educational attainment, first-trimester cigarette smoking, second-trimester cigarette smoking, yearly income and prenatal care appropriateness. After matching, survey-weighted logistic regression analyses were conducted without further covariate adjustment. | PRAMS-based retrospective self-report, no biochemical validation and possible overlap with other PRAMS studies. | Moderate to lower |
| 14. **Le (2025)^(14)^** | Prospective preconception cohort study (PRESTO)  USA & Canada  n=6,136 females  EV n=127  CC n=282  Dual n=55 | Exposure was assessed during the preconception period at baseline, before pregnancy. The study did not assess vaping or smoking changes after baseline, during conception, or during early pregnancy. | **EV:** preconception vaping was assessed at baseline among participants trying to conceive. Participants were classified as current vapers if they reported currently vaping >0 mL e-liquid/day, and former vapers if they had ever vaped but currently reported 0 mL/day. Ever vaping and current vaping amount were also analysed.  **Dual use:** not defined as a separate exposure group in the main analysis. Smoking and vaping were examined together in stratified and combined categories, including never/former/current vaping by never/ever smoking status, but dual users were not treated as a distinct primary comparison group.  **Non-use:** never vapers were used as the reference group. In some stratified analyses, never-vaping never-smokers were used as the lowest-exposure reference category.  **Smoking comparator groups:** combustible cigarette exposure was classified as never, former or current smoking. Current smoking was adjusted for in the main female models, and smoking history was explored as a potential modifier of the vaping association. | No | Yes/partial. Smoking and vaping histories used to define never smoker/never vaper categories. | Partly addressed. The study explicitly measured smoking history and current smoking, adjusted for current smoking, and stratified analyses by never/ever smoking status. This is stronger than studies that do not measure smoking. However, residual confounding remains possible because vaping and smoking were self-reported only at baseline, post-baseline changes before conception or in early pregnancy were not captured, and current/former smoking may not fully reflect lifetime tobacco exposure, intensity or timing relative to conception. | Adjusted Cox models included female age, education, race/ethnicity, current smoking status, BMI, alcohol use and history of spontaneous abortion. Couple-based models additionally included partner characteristics, including male smoking status and relevant reproductive/medical factors. | Prospective preconception design strengthens timing, but no biochemical validation. | Moderate |
| 15. **Lin (2023)^(15)^** | Retrospective cohort study using U.S. PATH survey data (2013–2018)  *USA*  n=1130  EV n=41  CC n=NR | Exposure was assessed before and during pregnancy using PATH survey data. Women were classified according to whether they never used e-cigarettes, quit before pregnancy, quit during pregnancy, or continued vaping during pregnancy. | **EV:** e-cigarette use was classified according to behavioural change before and during pregnancy: non-user, quit before pregnancy, quit during pregnancy, and continuous user. For the analysis of pregnancy outcomes, the authors collapsed this into non-user, quit before pregnancy, and any use during pregnancy, where “any use” included those who quit during pregnancy and those who continued vaping.  **Dual use:** not clearly separated as a distinct exposure group. Cigarette use was included as a separate covariate using parallel categories, including not using cigarettes, quit before pregnancy, and any use during pregnancy. The models also controlled for interactions between cigarette and e-cigarette use, but dual users were not presented as a standalone comparison group.  **Non-use:** women who did not currently use e-cigarettes and had not used them in the previous 12 months were classified as non-users.  **Smoking comparator groups:** cigarette smoking was categorised separately as not using, quit before pregnancy, or any use during pregnancy, but exclusive smoking was not used as a primary comparator group. | No | Partial. Quit before pregnancy and any use in pregnancy were separated, but exposure was self-reported. | Partly addressed. Cigarette use before and during pregnancy was measured and included in adjusted models, which helps account for concurrent or related smoking behaviour. However, residual confounding remains likely because dual use was not separated into a clear exposure group, smoking and vaping were self-reported, exposure categories were broad, and the study did not fully characterise smoking intensity, vaping frequency, nicotine concentration, device type or dose. Small numbers of pregnant e-cigarette users also limit interpretability. | Adjusted models included cigarette use, age, education, race, satisfaction with social activities and relationships, perception of e-cigarette harmfulness relative to cigarettes, whether e-cigarettes were allowed at home, advice to quit tobacco/e-cigarettes in pregnancy, physical and mental health before pregnancy, alcohol use, marijuana use, and interactions between cigarette and e-cigarette use and between e-cigarette use and race. | Small EV group, self-reported exposure and composite outcome. | Moderate to lower |
| 16. **McBride (2021)^(16)^** | Population-based cross-sectional study using U.S. PRAMS data (2016–2018)  *USA*  n=42,827  EV n= 385 | Exposure was assessed retrospectively for the last 3 months of pregnancy using PRAMS self-report. Although the response options captured frequency of ENDS use, the main exposure variable was dichotomised as any prenatal ENDS use versus no prenatal ENDS use. | **EV:** prenatal ENDS use was defined as any self-reported use of e-cigarettes or other electronic nicotine products during the last 3 months of pregnancy. Response options captured frequency, including no use, ≤1 day/week, 2–6 days/week, once daily, and more than once daily, but exposure was recoded as any use versus no use for the main analysis.   **Dual use:** not defined as a separate exposure group. Cigarette smoking during pregnancy was included as a covariate, but ENDS users were not separated into exclusive ENDS users and dual users.   **Non-use:** women who reported no ENDS use during the last 3 months of pregnancy. This was therefore no reported ENDS use rather than confirmed abstinence from all nicotine/tobacco products.   **Smoking comparator groups:** no separate combustible cigarette or dual-use comparator group was included. Prenatal cigarette smoking was measured as yes/no and adjusted for in the model | No | Partial. Dual use identified, but exposure based on retrospective self-report. | Partly addressed. Adjustment for prenatal cigarette smoking helps reduce confounding from combustible tobacco use, but residual confounding remains likely because cigarette smoking was treated only as yes/no and dual use was not separately classified. The study did not account for prior smoking history, smoking intensity, timing of cigarette use beyond “during pregnancy,” ENDS frequency in the main model, nicotine concentration, device type, or biochemical validation. | Adjusted for maternal age, race/ethnicity, marital status, education, parity, prenatal insurance, WIC receipt during pregnancy, quality of prenatal care using the Kotelchuck index, BMI, gestational weight gain, mode of delivery, gestational age, infant sex and prenatal cigarette smoking. | PRAMS-based retrospective self-report, no biochemical validation and breastfeeding outcome may be influenced by social and behavioral confounding. | Moderate to lower |
| 17. **McDonnell (2020)^(17)^** | Prospective observational cohort study  *Ireland*  n= 425  EV n= 218  CC n=99  Dual n=195 | Exposure was assessed at the booking history interview, generally between 10 and 14 weeks’ gestation. The study captured EC use and smoking status at this single early-pregnancy time point, not longitudinal exposure across pregnancy or status at delivery. | **EV:** exclusive electronic cigarette users were women who self-reported current EC use at the booking history interview and did not report concurrent combustible cigarette smoking. The final exclusive EC live singleton birth group included 218 women.   **Dual use:** defined as concurrent use of electronic cigarettes and combustible cigarettes at booking. Dual users were analysed separately from exclusive EC users.   **Non-use:** non-smokers were defined as women who had never smoked. The non-smoker comparison group therefore appears to be never-smokers rather than only current non-smokers.   **Smoking comparator groups:** smokers were defined as women currently smoking at least one cigarette per day. A separate dual-use group was also included. | No | Yes/partial. Dual use was identified at the time of survey. | Partly addressed. This study is stronger than many observational studies because it separated exclusive EC users from dual users and compared them with both current smokers and never-smokers. However, residual confounding remains possible because exposure was self-reported, no biochemical validation was used, previous smoking status among exclusive EC users was unclear, and some “never smoker” EC users may have represented undisclosed former smokers. Smoking and vaping status were also recorded only once, so later relapse, cessation or switching could not be captured. | Multiple logistic regression was used to account for potential confounding from observed differences in maternal age, parity, ethnicity and socioeconomic status. Birth centiles were also customised using GROW, which adjusted for infant sex, gestational age, ethnicity and maternal characteristics including parity and BMI. | Prospective design and large EV group, but no biochemical validation and exposure may change later in pregnancy. | Moderate |
| 18. **Nanninga (2023)^(18)^** | Cross-sectional study  *Netherlands*  n=1937  EV n=10  CC n=209 | Exposure was assessed retrospectively using an online questionnaire completed up to one year after the pregnancy. Smoking status referred to use during pregnancy, rather than a specific trimester or prospectively recorded time point. | **EV:** e-cigarette users were women who reported e-cigarette use during pregnancy. The EV group was very small, with 10 women, representing 0.5% of the included sample. Nicotine dose was recorded among e-cigarette users and categorised as none, low, medium or high.   **Dual use:** defined as use of both tobacco cigarettes and e-cigarettes during pregnancy. Dual users were identified as a separate group, with 12 women, representing 0.6% of the sample.   **Non-use:** non-smokers were women who did not report tobacco cigarette or e-cigarette use during pregnancy. The non-smoker group included 1706 women.   **Smoking comparator groups:** tobacco cigarette users were analysed as a separate group, with 209 women. The study therefore compared four smoking-status groups: non-smokers, tobacco cigarette users, e-cigarette users and dual users. | No | Partial. Dual use identified, but exposure based on postnatal self-report. | Partly addressed. The study separated e-cigarette users, tobacco cigarette users and dual users, and also collected previous smoking behaviour and second-hand smoke exposure. However, residual confounding remains substantial because the EV group was extremely small, analyses were unadjusted, and exposure was based on postnatal self-report. Previous smoking may have confounded comparisons, including among non-smokers, as 13.1% of non-smokers had smoked in the year before pregnancy. There was no biochemical validation. | No multivariable adjustment was performed. Demographic, lifestyle, pregnancy, maternal and infant characteristics were reported descriptively by smoking status, and group differences were assessed using chi-square or Fisher/Freeman-Halton exact tests. The authors state that the low power of the study precluded multivariate analyses. | Very small EV group, postnatal recall and no biochemical validation. | Lower |
| 19. **Nian (2025)^(19)^** | Descriptive surveillance analysis using PRAMS data (2016–2020)  *USA*  n=29,505  EV n=117  CC n=14,096  Dual n=278 | Exposure was assessed for the 3 months before pregnancy and the last 3 months of pregnancy using PRAMS self-report. Postpartum CC use was also assessed 2–6 months after delivery. The study therefore captures pre-pregnancy smoking, late-pregnancy EC/CC pattern and postnatal smoking pattern, but not detailed early- or mid-pregnancy EC use. | **EV:** the study focused on pregnant people who were exclusive combustible cigarette smokers in the 3 months before pregnancy and then initiated e-cigarette use during pregnancy. EC initiators were subdivided into **dual users**, who used both ECs and CCs in the last 3 months of pregnancy, and **EC replacers**, who discontinued CCs and used ECs exclusively in the last 3 months of pregnancy.   **Dual use:** explicitly defined as use of both ECs and CCs during the last 3 months of pregnancy. Most EC initiators were dual users rather than full replacers.   **Non-use:** this was not a general non-user/non-smoker reference group. The main comparison groups were **quitters**, who had used CCs only before pregnancy but reported neither EC nor CC use in the last 3 months of pregnancy, and **CC-exclusive users**, who continued combustible cigarette use without EC use.   **Smoking comparator groups:** CC-exclusive users were those who reported exclusive CC use in late pregnancy. Quitters were used as a lower-exposure comparison group, while CC-exclusive users represented continued smoking. | No | Yes/partial. Switching, dual use and EC replacement patterns were examined. | Better addressed than many PRAMS studies, but still only partially. The design specifically examined women who were exclusive CC smokers before pregnancy and then either continued CCs, quit, or initiated ECs, which makes switching, replacement and dual-use patterns clearer. However, exposure remained retrospectively self-reported, with no biochemical validation. EC initiation was assumed to represent a smoking cessation or reduction strategy, but intention to quit, use of other cessation supports, nicotine dose, EC quantity and early-pregnancy CC use were not fully captured. Residual confounding by motivation to quit and baseline dependence is likely. | Survey-weighted multivariable logistic regression adjusted for maternal age, race/ethnicity, education level, household income, marital status, prenatal WIC participation, pregnancy intention, flu vaccine receipt, Kotelchuck index, first-trimester prenatal care initiation, parity, history of preterm birth, prepregnancy BMI, pre-existing and/or gestational hypertension, pre-existing and/or gestational diabetes, self-reported depression before and/or during pregnancy, and year of delivery. The study also descriptively compared CC quantity before pregnancy, during late pregnancy and postpartum. | PRAMS-based retrospective self-report, no biochemical validation and descriptive design. | Moderate to lower |
| 20. **Opondo (2021)^(20)^** | Cross-sectional, population-based survey  *England*  n = 4,421  EV n=27 | Vaping exposure was assessed as any use after becoming aware of pregnancy. Smoking frequency was assessed across multiple time-points: 3 months before pregnancy, each trimester, and 3 and 6 months after birth. | **EV:** vaping was assessed by asking whether women used an electronic cigarette or vaping device after they found out they were pregnant. The study did not define a primary “exclusive vaping” group in the same way as some PRAMS studies; instead, vaping status was analysed alongside detailed smoking-pattern classes.   **Dual use:** dual use was not presented as a simple standalone exposure group, but overlap between vaping and smoking was explicitly examined through smoking-pattern classes. Vaping prevalence was reported across never-smokers, ex-smokers, pregnancy-inspired quitters, temporary quitters and persistent smokers.   **Non-use:** women who did not report vaping after becoming aware of pregnancy were treated as non-vapers. For smoking, never-smokers were identified separately through latent class analysis of smoking frequency before, during and after pregnancy.   **Smoking comparator groups:** smoking patterns were derived using latent class analysis and grouped as never-smokers, ex-smokers, pregnancy-inspired quitters, temporary quitters and persistent smokers. Smoking frequency was assessed from 3 months before pregnancy, across each trimester, and at 3 and 6 months postpartum. | No | Partial. Analyses adjusted for smoking patterns, but vaping exposure was self-reported. | Partly addressed. This study handled smoking more carefully than many survey studies because it modelled longitudinal smoking patterns before, during and after pregnancy, rather than simply adjusting for current smoking yes/no. However, residual confounding remains possible because vaping was self-reported as any use after pregnancy awareness, with no detail on frequency, dose, device type or nicotine concentration. Vaping and smoking data were retrospectively self-reported, no biochemical validation was used, and dual use was inferred through overlap with smoking-pattern classes rather than defined as a primary exposure category. | Adjusted for maternal sociodemographic characteristics associated with vaping, including age, country of birth, education and Index of Multiple Deprivation. Models also adjusted for smoking-pattern class derived from latent class analysis. For birth outcome and breastfeeding models, the authors used staged adjustment: unadjusted, adjusted for sociodemographic characteristics, adjusted for smoking pattern, and then adjusted for both sociodemographic characteristics and smoking pattern. Survey weights were used to account for non-response, based on age, country of birth, IMD, parity, marital status at birth registration and region of residence. | Small EV group, no biochemical validation and survey-based exposure assessment. | Moderate |
| 21. **Pesola (2024)^(21)^** | Secondary analysis of the PREP RCT  *UK*  n=1,140  EV/NRT n=166  CC n=322  Dual n=582 | Exposure was assessed after trial enrolment during pregnancy. Participants were recruited at 12–24 weeks’ gestation and followed to 35 weeks’ gestation/end of pregnancy, with product use and smoking status assessed during follow-up. The analysis therefore reflects nicotine-product use after the first trimester among women who were smokers at baseline. | **EV:** this was a secondary analysis of the PREP randomised controlled trial of pregnant smokers allocated to e-cigarettes or nicotine patches. For this analysis, exposure was based on actual regular use of nicotine products during pregnancy rather than randomised allocation. Regular EC use was defined by self-reported use for at least 5 consecutive days during the first 4 weeks, regular use for at least 1 week, occasional use for at least 3 weeks, or current use at end of pregnancy.   **Dual use:** dual use was explicitly examined. Participants were classified by end-of-pregnancy smoking status, including abstainers using nicotine products, smokers using nicotine products, and reducers. Smokers using EC or NRT at end of pregnancy were treated as dual users.   **Non-use:** there was no true pregnancy non-user/non-smoker comparison group because all participants were daily smokers at baseline. Comparisons were made between abstainers using nicotine products, abstainers not using nicotine products, smokers using nicotine products and smokers not using nicotine products.   **Smoking comparator groups:** smokers not using nicotine products regularly were compared with smokers using nicotine products regularly. Abstainers using nicotine products were also compared with abstainers not using nicotine products and with smokers. | Yes  Saliva cotinine and anabasine testing, and exhaled CO | Yes. All participants were smokers at baseline and switching/dual use was examined. | Relatively well addressed, but still partial. A key strength is that all participants were smokers at baseline, so comparisons are less confounded by never-smoker versus smoker differences. The study also included biochemical measures, including baseline salivary cotinine and end-of-pregnancy salivary cotinine in abstainers, reducers and dual users, plus trial measurement of smoking behaviour. However, product use was not randomised in this secondary analysis, EC and NRT were combined for some safety outcomes, abstinence was largely self-reported because of incomplete saliva return, and unmeasured differences in quitting motivation or pregnancy complications may still confound comparisons. | Pregnancy outcome analyses were adjusted for baseline cotinine level, occupation and Fagerström Test of Cigarette Dependence score, because these baseline characteristics differed between abstainers, nicotine-product users and smokers. Respiratory symptom analyses were adjusted for smoking status at end of pregnancy. Missing adjustment covariates were handled using multiple imputation. | Strong biochemical validation, but EV was combined with NRT and all participants were baseline smokers. | Higher |
| 22. **Regan & Bombard (2021)^(22)^** | Retrospective cohort study using PRAMS data (2016–2018)  *USA*  n = 79,176  3 months before pregnancy  EV n=462  CC n=13015  Dual n=1695  Last 3 months of pregnancy  EV n=329  CC n=5100  Dual n=577 | Exposure was assessed for two time windows: the 3 months before pregnancy and the last 3 months of pregnancy. The study could not directly assess e-cigarette use during early or mid-pregnancy. | **EV:** e-cigarette use was classified by timing as use in the 3 months before pregnancy only, or use during the last 3 months of pregnancy. Prenatal e-cigarette users were further classified by combustible cigarette smoking status. E-cigarette-only users were those who used e-cigarettes but did not smoke combustible cigarettes during the last 3 months of pregnancy. Frequency of e-cigarette use was also categorised as daily use, less than daily use, or no use.   **Dual use:** defined as use of both e-cigarettes and combustible cigarettes during the last 3 months of pregnancy. Dual users were analysed separately from e-cigarette-only users in stratified analyses.   **Non-use:** non-use was defined as no reported e-cigarette use in either the 3 months before pregnancy or the last 3 months of pregnancy. This does not necessarily mean no combustible cigarette use unless analyses were stratified by smoking status.   **Smoking comparator groups:** combustible cigarette smoking during pregnancy was measured and used both as an adjustment variable and as a stratification variable. Analyses compared e-cigarette-only users with non-users among those who did not smoke combustible cigarettes, and dual users with e-cigarette non-users among those who smoked combustible cigarettes. | No | Partial. Dual use and timing before/during pregnancy were identified. | Partly addressed. The study is stronger than simpler PRAMS analyses because it distinguishes prepregnancy e-cigarette use from late-pregnancy use, separates e-cigarette-only users from dual users, stratifies by combustible cigarette smoking, and considers frequency of e-cigarette use. However, residual confounding remains possible because exposure was retrospectively self-reported, no biochemical validation was used, and PRAMS only captured e-cigarette and combustible cigarette use for the 3 months before pregnancy and last 3 months of pregnancy. Earlier pregnancy exposure, nicotine content, device type, intensity of vaping, prior smoking history and possible non-disclosure were not fully captured. | Adjusted models controlled for maternal age, race/ethnicity, education, WIC use during pregnancy, adequacy of prenatal care, multivitamin use before pregnancy, and combustible cigarette smoking during pregnancy. Additional stratified analyses were performed by combustible cigarette smoking status during pregnancy, and frequency analyses examined daily versus less-than-daily e-cigarette use. The adjustment set was selected using a directed acyclic graph. | PRAMS-based retrospective self-report, no biochemical validation and possible dataset overlap. | Moderate to lower |
| 23. **Regan & Pereira (2021)^(23)^** | Retrospective cohort study using PRAMS data (2016–2018)  *USA*  N=16,022  Last 3 months of pregnancy  EV n=189  CC n=6310  Dual n=585 | Exposure was assessed for the 3 months before pregnancy and the last 3 months of pregnancy using PRAMS self-report. The study could not assess e-cigarette use earlier in pregnancy, although all included women had a history of combustible cigarette smoking before pregnancy. | **EV:** the study was restricted to women who had smoked combustible cigarettes before pregnancy. E-cigarette-only users were women who quit combustible cigarette smoking but reported e-cigarette use during the last 3 months of pregnancy. This group therefore represents switching from prior combustible cigarette use to self-reported e-cigarette-only use in late pregnancy, rather than lifelong exclusive vaping.   **Dual use:** defined as continued combustible cigarette smoking plus e-cigarette use during the last 3 months of pregnancy. Dual users were analysed as a separate group.   **Non-use:** the main lower-exposure comparator group was former smokers, defined as women who quit combustible cigarette smoking before/during pregnancy and did not use e-cigarettes. This is not a never-smoking/non-nicotine comparator group.   **Smoking comparator groups:** current smokers were women who continued combustible cigarette smoking during pregnancy and did not use e-cigarettes. The four mutually exclusive groups were former smokers, e-cigarette-only users, dual users and current smokers. | No | Yes/partial. Quitters, smokers and dual users were compared. | Partly addressed. The study specifically focused on women with prior combustible cigarette smoking and separated former smokers, e-cigarette-only users, dual users and current smokers, which makes switching and continued smoking patterns clearer than in analyses with a broad non-user comparator. However, residual confounding remains likely because exposure was self-reported, no biochemical validation was used, and e-cigarette exposure was only captured for the last 3 months of pregnancy. Prior smoking intensity, cessation motivation, reason for e-cigarette use, nicotine dose, device type and undisclosed smoking could still confound comparisons. | Adjusted models for birth outcomes controlled for maternal age, race/ethnicity, parity, adequacy of prenatal care, multivitamin use and presence of an obstetric risk factor. Models examining combustible cigarette smoking during pregnancy adjusted for maternal age, race/ethnicity, education, adequacy of prenatal care, multivitamin use, rurality of residence, WIC access and pregnancy intention. Adjustment variables were selected a priori using a directed acyclic graph. | PRAMS-based retrospective self-report, no biochemical validation and quitters used as comparator. | Moderate to lower |
| 24. **Shittu (2022)^(24)^** | Secondary analysis of a nationally representative cohort (PRAMS 2016–2018)  *USA*  n = 5,446,900  EV n= 61,173  CC n=773586  Dual n=149,152 | Exposure was assessed for the 3 months before pregnancy and the last 3 months of pregnancy. The study therefore captured changes between these two windows but could not determine the exact timing of quitting, switching or relapse during early or mid-pregnancy. | **EV:** exclusive e-cigarette users were women who reported e-cigarette use but no combustible cigarette use in the 3 months before pregnancy. Within this group, the study then examined changes during pregnancy, including continuing e-cigarette use, quitting e-cigarettes, switching from e-cigarettes to cigarettes, or initiating cigarette use alongside e-cigarettes.   **Dual use:** dual users were women who reported concurrent e-cigarette and combustible cigarette use. Among prepregnancy dual users, pregnancy changes were further classified as continuing both products, quitting e-cigarettes only, quitting cigarettes only, or quitting both.   **Non-use:** non-users were women who reported no e-cigarette or combustible cigarette use before or during pregnancy. Continuous non-users were used as the healthy reference group.   **Smoking comparator groups:** exclusive combustible cigarette smokers were women who reported cigarette use but no e-cigarette use before pregnancy. Pregnancy changes included continuing cigarette smoking, quitting cigarettes, switching completely to e-cigarettes, or initiating dual use. | No | Yes/partial. Changes in e-cigarette and cigarette use during pregnancy were analysed. | Partly addressed. This study is stronger than many PRAMS analyses because it explicitly models dynamic changes in e-cigarette and cigarette use from before pregnancy to late pregnancy, including quitting, continuing, switching and dual use. This helps distinguish exclusive vaping, exclusive smoking, dual use and non-use more clearly. However, residual confounding remains possible because exposure was retrospectively self-reported, no biochemical validation was used, and PRAMS did not capture detailed use in the first two trimesters. Frequency, nicotine concentration, device type, flavours, cannabis co-use, other tobacco/nicotine products, alcohol, caffeine, nutrition and environmental stress were also not fully accounted for. | Multivariable log-binomial regression models adjusted for significant covariates, including maternal age, race/ethnicity, education, type of health insurance, prepregnancy BMI, number of prenatal care visits, number of previous live births, gestational hypertension, gestational diabetes and infant sex. Income was not included because of missing data and was replaced by health insurance type as a proxy for poverty. | Large sample, but PRAMS-based self-report, no biochemical validation and potential overlap with other PRAMS analyses. | Moderate |
| 25. **Wang (2020)^(25)^** | Retrospective cohort study using PRAMS 2016 data  *USA*  n = 31,973  EV n= 267  CC n=2632  Dual n=265 | Exposure was assessed for the 3 months before pregnancy and the last 3 months of pregnancy using PRAMS self-report. The study could describe transitions between these two windows but could not characterise exposure during early or mid-pregnancy in detail. | **EV:** sole vapers were women who reported e-cigarette use but no combustible cigarette smoking. Exposure groups were created separately for the 3 months before pregnancy and the last 3 months of pregnancy. Among late-pregnancy users, sole vapers were those reporting e-cigarette use only in the last 3 months of pregnancy.   **Dual use:** dual users were women who reported both combustible cigarette smoking and e-cigarette use. The study also described transitions from prepregnancy dual use to late-pregnancy non-use, smoking only, vaping only or continued dual use.   **Non-use:** non-users were women who reported neither combustible cigarette smoking nor e-cigarette use. Late-pregnancy non-users were used as the reference group in outcome models.   **Smoking comparator groups:** sole smokers were women who reported combustible cigarette smoking without e-cigarette use. The study compared non-users, sole smokers, sole vapers and dual users, and additionally described changes in use from the 3 months before pregnancy to the last 3 months of pregnancy. | No | Partial. Dual use identified, but previous smoking and exposure history rely on retrospective self-report. | Partly addressed. The study separated sole vaping, sole smoking, dual use and non-use, and additionally adjusted for prepregnancy smoking/vaping status, which helps account for prior exposure history. However, residual confounding remains likely because exposure was retrospectively self-reported postpartum, no biochemical validation was used, and the study could not verify undisclosed smoking among self-reported sole vapers. It also lacked detail on nicotine content, device type, vaping intensity, cannabis or other substance use, and the heterogeneity of dual use makes interpretation difficult. | Multivariable models adjusted for maternal age, education, race/ethnicity, marital status, previous preterm birth, parity, Kotelchuck index of prenatal care, prepregnancy BMI, alcohol use before pregnancy and gestational weight gain. The fully adjusted model additionally adjusted for prepregnancy smoking/vaping status, categorised as non-user, sole smoker, sole vaper or dual user. | PRAMS-based retrospective self-report, no biochemical validation and potential overlap with later PRAMS studies. | Moderate to lower |
| 26. **Wang (2022)^(26)^** | Cohort study using US PRAMS data (2016–2018)  *USA*  n=99,201  EV n=968  CC n=15,662  Dual n= 2,629 | Exposure was assessed for the 3 months before pregnancy and the last 3 months of pregnancy. The analysis focused on dose/frequency during late pregnancy, with prepregnancy smoking/vaping included as an adjustment variable. It could not characterise exposure during early or mid-pregnancy in detail. | **EV:** sole vapers were women who reported e-cigarette use but no combustible cigarette smoking. The study further divided sole vapers into occasional vapers, defined as vaping less than once per day, and frequent vapers, defined as vaping at least once per day. Exposure groups were created for both the 3 months before pregnancy and the last 3 months of pregnancy.   **Dual use:** dual users were women who reported both combustible cigarette smoking and e-cigarette use. Unlike Wang 2020, this study subdivided dual users by smoking intensity and vaping frequency into light smoker/occasional vaper, light smoker/frequent vaper, heavy smoker/occasional vaper and heavy smoker/frequent vaper. Heavy smoking was defined as ≥6 cigarettes/day and frequent vaping as ≥once/day.   **Non-use:** non-users were women who reported neither combustible cigarette smoking nor e-cigarette use. Late-pregnancy non-users were used as the reference group.   **Smoking comparator groups:** sole smokers were divided into light smokers and heavy smokers according to average cigarettes smoked per day in the last 3 months of pregnancy. | No | Partial. Dual use identified and exposure-response analysed. | Partly addressed. The study improves on broader PRAMS analyses by examining exposure-response, separating smoking intensity from vaping frequency, and recognising that dual users are heterogeneous. It also adjusted for prepregnancy smoking/vaping status. However, residual confounding remains possible because exposure was self-reported postpartum, there was no biochemical validation, and the study could not distinguish nicotine-containing from nicotine-free vaping, device type, cannabis/THC use, or other unmeasured social and medical confounders. | Adjusted models controlled for birth year, maternal age, education, race/ethnicity, marital status, parity, previous preterm birth, Kotelchuck index of prenatal care, prepregnancy BMI and prepregnancy smoking/vaping status. Sensitivity analyses were also conducted among younger mothers and non-Hispanic White mothers. | PRAMS-based self-report, no biochemical validation and potential overlap with other PRAMS studies. | Moderate |
| 27. **Wen & Liu (2023)^(27)^** | Retrospective cohort study using US PRAMS data (2016–2021).  *USA*  n=10,428 EV n=152  CC n=719  Dual n=125 | Exposure was assessed for the last 3 months of pregnancy only. PRAMS did not provide e-cigarette or cigarette use data for the first or second trimester in this adolescent-focused analysis, so continuous use throughout pregnancy could not be distinguished from late-pregnancy-only use. | **EV:** exclusive e-cigarette users were pregnant adolescents aged 10–19 years who reported e-cigarette use but no combustible cigarette smoking during the last 3 months of pregnancy. E-cigarette frequency was collected using PRAMS categories, but was dichotomised as use versus non-use for the main analyses because of limited sample size.   **Dual use:** dual users were adolescents who reported both e-cigarette use and combustible cigarette smoking during the last 3 months of pregnancy. Dual use was analysed as a separate exposure group.   **Non-use:** non-users were adolescents who reported no e-cigarette use and no combustible cigarette smoking during the last 3 months of pregnancy.   **Smoking comparator groups:** exclusive cigarette users were adolescents who reported combustible cigarette smoking but no e-cigarette use during the last 3 months of pregnancy. The four mutually exclusive groups were non-users, exclusive e-cigarette users, exclusive cigarette users and dual users. | No | Partial. Dual use identified, but exposure based on self-report. | Partly addressed. The study separated exclusive e-cigarette use, exclusive cigarette use, dual use and non-use, which reduces confounding from concurrent smoking. However, residual confounding remains likely because exposure was self-reported postpartum, no biochemical validation was used, and data were limited to late pregnancy. The study could not assess first- or second-trimester exposure, cumulative exposure, second-hand smoke, device type, nicotine concentration, cannabis use, other substances or diet quality. | Adjusted logistic regression models controlled for maternal age, race, ethnicity, marital status, health insurance, prepregnancy BMI, prepregnancy diabetes, prepregnancy hypertension and child’s birth year. Sampling weights were used to reduce bias from non-random sampling, non-coverage and non-response. | PRAMS-based self-report, no biochemical validation and restricted adolescent population may limit generalisability. | Moderate to lower |
| 28. **Wen & Thomas (2023)^(28)^** | Secondary analysis of PRAMS cohort data (2016–2020).  *USA*  n = 176,882  EV n= 921  CC n= 13,697  Dual n= 1,308 | Exposure was assessed for the last 3 months of pregnancy only. Although PRAMS includes questions on use before pregnancy, this analysis focused on late-pregnancy e-cigarette and cigarette use in relation to low gestational weight gain. Early- and mid-pregnancy exposure could not be assessed. | **EV:** exclusive e-cigarette users were women who reported e-cigarette use but no combustible cigarette smoking during the last 3 months of pregnancy. Original PRAMS frequency categories were collected, but exposure was dichotomised as any use versus non-use for the main analysis.   **Dual use:** dual users were women who reported both e-cigarette use and combustible cigarette smoking during the last 3 months of pregnancy. Dual use was analysed as a separate exposure group.   **Non-use:** non-users were women who reported neither e-cigarette use nor combustible cigarette smoking during the last 3 months of pregnancy.   **Smoking comparator groups:** exclusive cigarette users were women who reported combustible cigarette smoking but no e-cigarette use during the last 3 months of pregnancy. The four mutually exclusive groups were non-users, exclusive e-cigarette users, exclusive cigarette users and dual users. | No | Partial. Dual use identified, but exposure based on retrospective self-report. | Adjusted logistic regression models controlled for maternal race/ethnicity, age, marital status, education, health insurance, prepregnancy BMI, prepregnancy diabetes and prepregnancy hypertension. Sampling weights were used to account for non-random sampling, non-coverage and non-response. | Adjusted logistic regression models controlled for maternal race/ethnicity, age, marital status, education, health insurance, prepregnancy BMI, prepregnancy diabetes and prepregnancy hypertension. Sampling weights were used to account for non-random sampling, non-coverage and non-response. | PRAMS-based self-report, no biochemical validation and outcome focused on gestational weight gain. | Moderate to lower |
| 29. **Wen & Kwak (2024)^(29)^** | Retrospective cohort using PRAMS (2016–2021).  *USA*  n=194,762  EV n=2,921  CC n=20,255  Dual n=3,700 | Exposure was assessed before pregnancy and during late pregnancy using PRAMS self-report. The analysis focused on changes in EC and/or CC use during pregnancy, including continuation versus discontinuation. | **EV:** exclusive e-cigarette users were mothers who reported e-cigarette use before pregnancy without combustible cigarette use. Among prepregnancy exclusive EC users, the study compared those who continued EC use during pregnancy with those who discontinued EC use.   **Dual use:** dual users were mothers who reported both e-cigarette and combustible cigarette use before pregnancy. Among prepregnancy dual users, the study compared continuation of dual use with discontinuation of both products, discontinuation of EC only, or discontinuation of CC only.   **Non-use:** non-users were mothers who reported no e-cigarette or combustible cigarette use. The prevalence of postpartum depression among non-users was reported as the baseline comparison, although the main analyses focused on changes within prepregnancy EC, CC and dual-use groups.   **Smoking comparator groups:** exclusive combustible cigarette users were mothers who reported CC use before pregnancy without EC use. Among prepregnancy exclusive CC users, the study compared continuation versus discontinuation of CC use during pregnancy.. | No | Yes/partial. Continuation and cessation of vaping, smoking and dual use were examined. | Partly addressed. The study examined continuation and cessation patterns among prepregnancy exclusive EC users, exclusive CC users and dual users, which helps distinguish ongoing exposure from discontinuation. However, residual confounding remains likely because exposure was based on retrospective PRAMS self-report, no biochemical validation was used, and postpartum depression is strongly influenced by baseline mental health, social adversity, substance use, relationship factors and healthcare access. Although prepregnancy depression was adjusted for, the abstract does not provide enough detail to assess control for all relevant confounders. | Multivariable logistic regression models adjusted for pre-existing or prepregnancy depression, sociodemographic characteristics, pregnancy characteristics and substance use characteristics. The abstract does not provide the full covariate list, so the adjustment set cannot be fully appraised from the available information. | PRAMS-based self-report, no biochemical validation and postpartum depression outcome may be affected by residual confounding. | Moderate |
| 30. **Wen & Moe (2024)^(30)^** | Retrospective cohort using PRAMS (2016–2021), linked to birth certificates.  *USA*  n=223,805.  EV n=4,090.  CC n=30,809.  Dual n=6,428; | Exposure was assessed before pregnancy and during late pregnancy using PRAMS self-report. The analysis focused on change in use during pregnancy, including continuation versus discontinuation of e-cigarettes, cigarettes or both. | **EV:** exclusive e-cigarette users were participants who reported e-cigarette use before pregnancy without combustible cigarette use. Among prepregnancy exclusive EC users, the study compared those who continued vaping during pregnancy with those who discontinued EC use.   **Dual use:** dual users were participants who reported both e-cigarette and combustible cigarette use before pregnancy. Among prepregnancy dual users, the study compared continued dual use with continued smoking only, continued vaping only, and complete cessation of both products.   **Non-use:** non-users were participants who reported no e-cigarette or combustible cigarette use. The prevalence of preterm birth among non-users was reported as the lower-exposure comparison, although the main analyses focused on continuation versus cessation within prepregnancy EC, CC and dual-use groups.   **Smoking comparator groups:** exclusive cigarette users were participants who reported combustible cigarette use before pregnancy without e-cigarette use. Among prepregnancy exclusive CC users, the study compared continued smoking with smoking cessation during pregnancy. | No | Yes/partial. Continuation and cessation during pregnancy were examined. | Partly addressed. The study examined continuation and cessation patterns among prepregnancy exclusive EC users, exclusive CC users and dual users, which helps account for prior smoking/vaping status and distinguishes continued exposure from cessation. However, residual confounding remains possible because exposure was based on retrospective PRAMS self-report, there was no biochemical validation, and cessation/continuation may be influenced by unmeasured factors such as nicotine dependence, health behaviours, pregnancy complications, social adversity and motivation to quit. | Multivariable logistic regression models adjusted for sociodemographic characteristics, pregnancy-related characteristics and other substance use. Gestational age and preterm birth status were obtained from linked birth certificate data, which strengthens outcome ascertainment. The abstract does not provide the full covariate list, so the adjustment strategy cannot be fully appraised from the available information. | PRAMS-based self-report, no biochemical validation and potential overlap with other PRAMS studies, although birth certificate linkage strengthens outcome ascertainment. | Moderate |

## Abbreviations: ANCOVA = analysis of covariance; BMI = body mass index; CC = combustible cigarette; CO = carbon monoxide; EC = electronic cigarette; ENDS = electronic nicotine delivery system; EV = exclusive vaping; GWG = gestational weight gain; IMD = Index of Multiple Deprivation; NBAS = Neonatal Behavioural Assessment Scale; NR = not reported; NRT = nicotine replacement therapy; PATH = Population Assessment of Tobacco and Health; PRAMS = Pregnancy Risk Assessment Monitoring System; PREP = Pregnancy Trial of E-cigarettes and Patches; PRESTO = Pregnancy Study Online; RCT = randomised controlled trial; THC = tetrahydrocannabinol; WIC = Special Supplemental Nutrition Program for Women, Infants, and Children.

## References:

1.	Ammar L, Tindle HA, Miller AM, Adgent MA, Nian H, Ryckman KK, et al. Electronic cigarette use during pregnancy and the risk of adverse birth outcomes: A cross-sectional surveillance study of the US Pregnancy Risk Assessment Monitoring System (PRAMS) population. PloS one. 2023;18(10):e0287348.

2.	Ashford K, McCubbin A, Barnett J, Blair LM, Lei F, Bush H, et al. Longitudinal examination of prenatal tobacco switching behaviors and birth outcomes, including Electronic Nicotine Delivery System (ENDS) and dual use. Maternal and child health journal. 2021;25(8):1175-81.

3.	Azar M, Oatey ME, Moniz MH, Bailey BA. Intrapartum Electronic Cigarette Use and Birth Outcomes: Evidence from a Population-Based Study. International Journal of Environmental Research and Public Health. 2024;21(11):1449.

4.	Bailey BA, Azar M, Nadolski K, Dodge P. Fetal Growth Following Electronic Cigarette Use in Pregnancy. International Journal of Environmental Research and Public Health. 2024;21(9):1179.

5.	Cardenas VM, Cen R, Clemens MM, Moody HL, Ekanem US, Policherla A, et al. Use of Electronic Nicotine Delivery Systems (ENDS) by pregnant women I: Risk of small-for-gestational-age birth. Tobacco induced diseases. 2019;17:44.

6.	Cardenas VM, Ali MM, Fischbach LA, Nembhard WN. Dual use of cigarettes and electronic nicotine delivery systems during pregnancy and the risk of small for gestational age neonates. Annals of epidemiology. 2020;52:86-92. e2.

7.	Choi BM, Weinberger AH, Petersen N, Pang RD, DeVito EE, Bell ML, et al. Association of e-cigarette use and postpartum depression: pregnancy risk assessment monitoring system 2016–2019. Journal of Women's Health. 2024;33(1):45-51.

8.	Cui Y, Visser A, Tsaih S-W, Palatnik A. Adverse birth outcomes associated with use of nicotine products during pregnancy. American Journal of Obstetrics & Gynecology. 2022;226(1):S148.

9.	Froggatt S, Reissland N, Covey J. The effects of prenatal cigarette and e-cigarette exposure on infant neurobehaviour: A comparison to a control group. EClinicalMedicine. 2020; 28: 100602. 2020.

10.	Froggatt S, Reissland N, Covey J, Kumarendran K. Foetal mouth movements: Effects of nicotine. Acta paediatrica. 2021;110(11):3014-20.

11.	Galbo A, Izhakoff N, Courington C, Castro G, Lozano J, Ruiz-Pelaez J. The association between electronic cigarette use during pregnancy and unfavorable birth outcomes. Cureus. 2022;14(7).

12.	Hawkins SS, Wylie BJ, Hacker MR. Associations between electronic nicotine delivery systems and birth outcomes. The journal of maternal-fetal & neonatal medicine. 2022;35(25):6868-75.

13.	Kim S, Oancea SC. Electronic cigarettes may not be a “safer alternative” of conventional cigarettes during pregnancy: evidence from the nationally representative PRAMS data. BMC pregnancy and childbirth. 2020;20:1-9.

14.	Le AD, Zhang CA, Chen AL, Basran S, Seranio N, Scott M, et al. A Preconception Cohort Study of Nicotine Vaping and Incidence of Spontaneous Abortion. Nicotine and Tobacco Research. 2025:ntaf141.

15.	Lin S-y, Wang L, Zhou W, Kitsantas P, Wen X, Xue H. E-cigarette use during pregnancy and its association with adverse birth outcomes in the US. Preventive medicine. 2023;166:107375.

16.	McBride M, Haile ZT. Association between electronic nicotine delivery systems use and breastfeeding duration. Breastfeeding Medicine. 2021;16(11):886-93.

17.	McDonnell B, Dicker P, Regan C. Electronic cigarettes and obstetric outcomes: a prospective observational study. BJOG: An International Journal of Obstetrics & Gynaecology. 2020;127(6):750-6.

18.	Nanninga EK, Weiland S, Berger MY, Feijen-de Jong EI, Erwich JJH, Peters LL. Adverse maternal and infant outcomes of women who differ in smoking status: E-cigarette and tobacco cigarette users. International Journal of Environmental Research and Public Health. 2023;20(3):2632.

19.	Nian H, Odland R, Mindlin S, Ammar L, Tindle H, Miller AM, et al. Demographic characteristics, perinatal smoking patterns, and risk for neonatal health complications among pregnant smokers in the United States who begin using electronic cigarettes during pregnancy: a descriptive study using population-based surveillance data. Nicotine and Tobacco Research. 2024;26(11):1455-62.

20.	Opondo C, Harrison S, Alderdice F, Carson C, Quigley MA. Electronic cigarette use (vaping) and patterns of tobacco cigarette smoking in pregnancy–evidence from a population-based maternity survey in England. PloS one. 2021;16(6):e0252817.

21.	Pesola F, Smith KM, Phillips‐Waller A, Przulj D, Griffiths C, Walton R, et al. Safety of e‐cigarettes and nicotine patches as stop‐smoking aids in pregnancy: Secondary analysis of the Pregnancy Trial of E‐cigarettes and Patches (PREP) randomized controlled trial. Addiction. 2024;119(5):875-84.

22.	Regan AK, Bombard JM, O’Hegarty MM, Smith RA, Tong VT. Adverse birth outcomes associated with prepregnancy and prenatal electronic cigarette use. Obstetrics & Gynecology. 2021;138(1):85-94.

23.	Regan AK, Pereira G. Patterns of combustible and electronic cigarette use during pregnancy and associated pregnancy outcomes. Scientific reports. 2021;11(1):13508.

24.	Shittu AAT, Kumar BP, Okafor U, Berkelhamer SK, Goniewicz ML, Wen X. Changes in e-cigarette and cigarette use during pregnancy and their association with small-for-gestational-age birth. American journal of obstetrics and gynecology. 2022;226(5):730. e1-. e10.

25.	Wang X, Lee NL, Burstyn I. Smoking and use of electronic cigarettes (vaping) in relation to preterm birth and small-for-gestational-age in a 2016 US national sample. Preventive medicine. 2020;134:106041.

26.	Wang X, Lee NL, Burstyn I. Exposure-response analysis of the association of maternal smoking and use of electronic cigarettes (vaping) in relation to preterm birth and small-for-gestational-age in a national US sample, 2016–2018. Global Epidemiology. 2022;4:100079.

27.	Wen X, Liu L, Moe AA, Ormond IK, Shuren CC, Scott IYN, et al. Use of e-cigarettes and cigarettes during late pregnancy among adolescents. JAMA Network Open. 2023;6(12):e2347407-e.

28.	Wen X, Thomas MA, Liu L, Moe AA, Duong PH, Griffiths ME, et al. Association between maternal e‐cigarette use during pregnancy and low gestational weight gain. International Journal of Gynecology & Obstetrics. 2023;162(1):300-8.

29.	Wen X, Liu L, Kwak N, Chaudhuri P, Panzica H. CHANGE IN E-CIGARETTE AND CIGARETTE USE DURING PREGNANCY AND POSTPARTUM DEPRESSION. Chest. 2024;166(4):A6547.

30.	Wen X, Moe A, Patel D, Hastings G, Manney K. CHANGE IN MATERNAL E-CIGARETTE USE DURING PREGNANCY AND PRETERM BIRTH. Chest. 2024;166(4):A6546.
