# Supplementary material for: Vaping during pregnancy and maternal, fetal, and infant health outcomes: A scoping review of human and animal studies
Source: PLoS One. 2026 Sep 29;21(9):e0356305. doi: 10.1371/journal.pone.0356305 (PMC13623096; doi:10.1371/journal.pone.0356305)
Supplement: Supplementary file 5 [file pone.0356305.s005.docx]

Maternal & Fetal Health Research Centre

School of Medical Sciences

The University of Manchester

c/o Research Floor

St Mary’s Hospital

Oxford Road,

Manchester	M13 9WL

24^th^ July 2026

Dear Eman Allam,

Below we detail all changes made in the revision and responses to all reviewer comments. We hope that these amendments make the manuscript suitable for publication in PLOS ONE.

Journal requirements:

When submitting your revision, we need you to address these additional requirements.

1. Please ensure that your manuscript meets PLOS ONE's style requirements, including those for file naming. The PLOS ONE style templates can be found at

[https://journals.plos.org/plosone/s/file?id=wjVg/PLOSOne_formatting_sample_main_body.pdf [journals.plos.org]](https://eur01.safelinks.protection.outlook.com/?url=https%3A%2F%2Furldefense.com%2Fv3%2F__https%3A%2F%2Fjournals.plos.org%2Fplosone%2Fs%2Ffile%3Fid%3DwjVg*PLOSOne_formatting_sample_main_body.pdf__%3BLw!!PDiH4ENfjr2_Jw!Fhl0fQshntf5LhLpcvpuS8mIS-ol2N24Mk3cAU0x04YFvHi8hkyzVcQHrr2BS8XjEcQtQwbXtPBwon1aZIlQLvu6NtSfSfnjAzQ%24&data=05%7C02%7Cstella.fielder%40mft.nhs.uk%7C4c128489a0bd4fe3723608debf1eafe8%7Cddc77078e8034eeb80cadd03ba7459c4%7C0%7C0%7C639158335702379713%7CUnknown%7CTWFpbGZsb3d8eyJFbXB0eU1hcGkiOnRydWUsIlYiOiIwLjAuMDAwMCIsIlAiOiJXaW4zMiIsIkFOIjoiTWFpbCIsIldUIjoyfQ%3D%3D%7C0%7C%7C%7C&sdata=LqVZZV8sqXp6yhOhotWHcJLSlHpxkGWvj4zgXx%2FGB1s%3D&reserved=0) and

[https://journals.plos.org/plosone/s/file?id=ba62/PLOSOne_formatting_sample_title_authors_affiliations.pdf [journals.plos.org]](https://eur01.safelinks.protection.outlook.com/?url=https%3A%2F%2Furldefense.com%2Fv3%2F__https%3A%2F%2Fjournals.plos.org%2Fplosone%2Fs%2Ffile%3Fid%3Dba62*PLOSOne_formatting_sample_title_authors_affiliations.pdf__%3BLw!!PDiH4ENfjr2_Jw!Fhl0fQshntf5LhLpcvpuS8mIS-ol2N24Mk3cAU0x04YFvHi8hkyzVcQHrr2BS8XjEcQtQwbXtPBwon1aZIlQLvu6NtSfbws0h08%24&data=05%7C02%7Cstella.fielder%40mft.nhs.uk%7C4c128489a0bd4fe3723608debf1eafe8%7Cddc77078e8034eeb80cadd03ba7459c4%7C0%7C0%7C639158335702396452%7CUnknown%7CTWFpbGZsb3d8eyJFbXB0eU1hcGkiOnRydWUsIlYiOiIwLjAuMDAwMCIsIlAiOiJXaW4zMiIsIkFOIjoiTWFpbCIsIldUIjoyfQ%3D%3D%7C0%7C%7C%7C&sdata=EdLXUeLB3zdaFpZk%2BCTAmkQpq0tn6CMYu5eQ4J0AZJE%3D&reserved=0)

We have revised the manuscript to align with PLOS ONE formatting requirements. In particular, we have changed the “Methods” heading to “Materials and methods”, standardised figure and table captions, updated in-text citations to PLOS style, and ensured that Supporting Information files are cited as S1 Appendix, S2 Appendix, S3 Appendix and S4 Appendix. We have prepared both a clean manuscript and a tracked-changes version for submission, as requested.

**Location in revised manuscript: throughout the revised manuscript (pp. 3–46).**

2. When completing the data availability statement of the submission form, you indicated that you will make your data available on acceptance. We strongly recommend all authors decide on a data sharing plan before acceptance, as the process can be lengthy and hold up publication timelines. Please note that, though access restrictions are acceptable now, your entire data will need to be made freely accessible if your manuscript is accepted for publication. This policy applies to all data except where public deposition would breach compliance with the protocol approved by your research ethics board. If you are unable to adhere to our open data policy, please kindly revise your statement to explain your reasoning and we will seek the editor's input on an exemption. Please be assured that, once you have provided your new statement, the assessment of your exemption will not hold up the peer review process.

We have revised the data availability statement to clarify that all data underlying the findings of this review are available within the manuscript and Supporting Information files. These include the full search strategies, PRISMA-ScR checklist, extracted study characteristics and outcomes, and structured study appraisal and exposure classification. As this is a scoping review, no individual participant data were generated or analysed by the authors.

The revised statement reads:

“All data underlying the findings of this review are available within the manuscript and Supporting Information files. These include the full search strategies, PRISMA-ScR checklist, extracted study characteristics and outcomes, and structured study appraisal and exposure classification. No individual participant data were generated or analysed by the authors for this review.”

We have also added a code availability statement clarifying that no custom code was used beyond spreadsheet formulas to aggregate counts and produce tables.

**Location in revised manuscript: p. 2.**

3. Please include captions for your Supporting Information files at the end of your manuscript, and update any in-text citations to match accordingly. Please see our Supporting Information guidelines for more information: [http://journals.plos.org/plosone/s/supporting-information [journals.plos.org]](https://eur01.safelinks.protection.outlook.com/?url=https%3A%2F%2Furldefense.com%2Fv3%2F__http%3A%2F%2Fjournals.plos.org%2Fplosone%2Fs%2Fsupporting-information__%3B!!PDiH4ENfjr2_Jw!Fhl0fQshntf5LhLpcvpuS8mIS-ol2N24Mk3cAU0x04YFvHi8hkyzVcQHrr2BS8XjEcQtQwbXtPBwon1aZIlQLvu6NtSf8GeN7Ac%24&data=05%7C02%7Cstella.fielder%40mft.nhs.uk%7C4c128489a0bd4fe3723608debf1eafe8%7Cddc77078e8034eeb80cadd03ba7459c4%7C0%7C0%7C639158335702415787%7CUnknown%7CTWFpbGZsb3d8eyJFbXB0eU1hcGkiOnRydWUsIlYiOiIwLjAuMDAwMCIsIlAiOiJXaW4zMiIsIkFOIjoiTWFpbCIsIldUIjoyfQ%3D%3D%7C0%7C%7C%7C&sdata=INQ717pMzTOLJTd4veQV%2Bs4ggk2im3IiZYIgD2mUdgM%3D&reserved=0).

We have added a Supporting Information section at the end of the manuscript with captions for S1–S4 Appendices. We have also updated the in-text citations to match PLOS ONE Supporting Information style.

**Location in revised manuscript: Supporting Information captions on p. 35**

**Reviewers' comments:**

**Comments to the Author**

**Reviewer #1: General Assessment**

This manuscript presents a timely and important scoping review addressing the growing public health concern of vaping during pregnancy. The inclusion of both human and animal studies is a notable strength, allowing exploration of translational gaps between mechanistic and clinical evidence. The study is generally well-structured and follows JBI and PRISMA-ScR guidance, with a clearly stated objective and comprehensive search strategy

However, while the manuscript successfully maps the literature, there are important methodological, conceptual, and reporting issues that need to be addressed to strengthen rigor, interpretability, and contribution to the field.

We thank the reviewer for this helpful assessment and for recognising the importance of the topic, the value of combining human and animal evidence. We have made multiple revisions throughout the manuscript in line with their recommendations as detailed below.

**Location in revised manuscript: revisions throughout pp. 3–37.**

Major Comments

**1. Lack of Critical Appraisal Limits Interpretability**

Although scoping reviews do not require formal risk-of-bias assessment, the manuscript goes beyond mapping and makes interpretive statements (e.g., “insufficient to draw firm conclusions”).

Without at least a basic quality stratification, readers cannot judge:

Strength of evidence

Reliability of associations (especially PRAMS vs prospective cohorts)

Recommendation:

Include a structured appraisal (even simplified) such as:
Study design hierarchy
Exposure validity (biochemical vs self-report)
Confounding control
Alternatively, explicitly limit interpretive language and remain descriptive.

We agree that, although formal risk-of-bias assessment is not required for scoping reviews, additional structured appraisal would help readers interpret the evidence. We have therefore added a structured interpretability appraisal of the included human studies, presented as S4 Appendix.

This appraisal includes study design and data source, exposure timing, definitions of exclusive vaping, dual use and non-use, biochemical validation, whether previous smoking or dual use was addressed, residual confounding from previous or concurrent combustible cigarette use, confounder adjustment, the main interpretability limitation and an overall descriptive interpretability rating.

We have also added explanatory text in the Materials and methods section to clarify the rationale for this approach. The revised manuscript states that the appraisal was not intended to produce a formal risk-of-bias score, but to map the methodological features most relevant to interpreting associations between vaping and pregnancy outcomes.

We have also added a signposting sentence in the Results section and expanded the “Methodological characteristics of included human studies” section to summarise the findings of this appraisal. Finally, we revised interpretive language in the Abstract, Discussion and Conclusion to ensure that conclusions remain cautious and proportionate to the evidence base.

**Location in revised manuscript: Materials and methods p. 10; Results p. 12; Methodological characteristics pp. 29–31; Abstract pp. 3–4; Discussion pp. 31–34; Conclusion pp. 35–37; and S4 Appendix.**

**2. Over-reliance on PRAMS Data Not Adequately Problematised**

The manuscript identifies that many findings come from PRAMS datasets but does not fully interrogate this bias.

Key issue:

PRAMS = retrospective self-report + recall bias + misclassification
Multiple studies using the same dataset may give false impression of evidence volume

Recommendation:
Add a dedicated subsection on:
Data source clustering
Risk of duplication bias
Quantify proportion of findings derived from PRAMS vs independent datasets.

We have added a dedicated paragraph in the Discussion addressing data-source clustering. The revised Discussion explains that repeated analyses of overlapping PRAMS years should not be interpreted as fully independent evidence because the same underlying limitations may be reproduced across multiple publications. These limitations include reliance on postpartum recall, self-reported smoking and vaping status, limited detail on vaping patterns, incomplete capture of earlier pregnancy smoking and absence of biochemical validation.

We also now state that this clustering may give an inflated impression of evidence volume, particularly for low birth weight, preterm birth, small for gestational age, postpartum depression and gestational weight gain outcomes. This revision directly addresses the concern that repeated analyses of the same data source may affect interpretation of the apparent consistency or volume of evidence.

**Location in revised manuscript: Discussion pp. 31–32.**

**3. Exposure Classification is a Critical Weakness but Not Fully Analysed**

The manuscript repeatedly notes heterogeneity in:
Exclusive vaping (EV)
Dual use
Smoking history
However, this is not systematically synthesised.

Problem:
Misclassification (e.g., “exclusive vapers” who are ex-smokers) can inflate risk estimates

Recommendation:
Create a table or framework summarising:
How EV, dual use, and non-use were defined across studies
Explicitly discuss:
Residual confounding from prior smoking
Timing of exposure (pre-pregnancy vs during pregnancy)

Thank you. We have addressed this through the addition of the table in S4 Appendix and through expanded synthesis in the main manuscript.

S4 Appendix provides a structured study-level framework summarising how exclusive vaping, dual use and non-use were defined across included human studies. It also records exposure timing, whether biochemical validation was used, whether previous smoking or dual use was addressed, residual confounding from previous or concurrent combustible cigarette use, confounder adjustment and overall interpretability.

In the Results section, we have expanded the “Methodological characteristics of included human studies” subsection to summarise these issues. The revised manuscript now explains that in several PRAMS analyses, exclusive vaping referred to self-reported e-cigarette use without concurrent combustible cigarette smoking during the last three months of pregnancy, rather than lifelong exclusive vaping or biochemically confirmed abstinence from tobacco. We also discuss how some studies included former smokers, quitters or participants with unclear previous smoking history within comparator groups, and that non-use was inconsistently defined across studies.

We have also expanded the Discussion to emphasise that exposure misclassification and residual confounding from prior smoking limit interpretation of apparent associations, particularly where studies classified participants using late-pregnancy exposure only.

**Location in revised manuscript: Methodological characteristics pp. 29–30; Discussion pp. 32–34; and S4 Appendix.**

**4. Combining Human and Animal Evidence Needs Stronger Conceptual Framing**

The inclusion of animal studies is valuable but currently feels parallel rather than integrated.

Issue:

Limited synthesis of:
How mechanistic findings relate to human outcomes
What can/cannot be translated

Recommendation:

Add a dedicated “Translational Interpretation” section:
Which pathways (e.g., oxidative stress, endothelial dysfunction) are plausible in humans
Which findings are likely artefacts of high-dose models

We agree and have substantially revised the Discussion to improve animal-human translational framing. We now explicitly discuss mechanistic pathways identified in animal studies, including oxidative stress, inflammation, endothelial dysfunction, altered placental perfusion, metabolic dysregulation, epigenetic modification and neurodevelopmental disruption. We then link these pathways to clinically relevant human outcomes, including fetal growth restriction, hypertensive disorders, placental insufficiency, respiratory morbidity and altered neurodevelopment.

We have also added clearer caution around translation. The revised Discussion states that animal findings should be interpreted as mechanistic and hypothesis-generating rather than confirmatory evidence of clinical risk in humans. We specifically identify limitations including physiological differences, species-specific placental structure, differences in nicotine metabolism, nicotine-naïve dams and exposure paradigms involving continuous or high-concentration aerosol exposure that may not reflect typical human use.

**Location in revised manuscript: Evidence map pp. 12–13; Discussion pp. 31–32.**

**5. Conclusions Slightly Overreach the Evidence**

The statement:

“current literature is insufficient to draw firm conclusions regarding risk”

is reasonable, but elsewhere the manuscript implies potential harm based on animal data.

Issue:

Risk of implicit causal inference from heterogeneous and low-quality evidence

Recommendation:

Clearly separate:
Evidence gaps
Hypothesised risks
Use more cautious phrasing:
“suggestive but inconclusive”
“biologically plausible but not confirmed in humans”

We have revised the Abstract, Discussion and Conclusion to ensure that conclusions are cautious and do not imply causal inference from heterogeneous evidence. The revised manuscript now states that animal studies identify biologically plausible mechanisms of harm, but that these findings remain hypothesis-generating and have not been consistently confirmed in human pregnancy studies. We have also clarified that direct translation is limited by physiological differences, species-specific placental structure, differences in nicotine metabolism and exposure paradigms that may not reflect typical human use.

We have revised comparative statements to avoid overstatement. For example, the Discussion now states that although several studies reported lower levels of association for exclusive vaping compared with combustible cigarette use across low birth weight and preterm birth outcomes, confidence intervals frequently overlapped and exposure misclassification limits firm conclusions. We also state that absence of evidence should not be interpreted as evidence of safety.

**Location in revised manuscript: Abstract pp. 3–4; Discussion pp. 31–33; Conclusion pp. 34.**

**6. Maternal Outcomes Are Underdeveloped**

The review highlights limited maternal data but does not deeply analyse it.

Given your expertise in maternal health, this is a missed opportunity.

Recommendation:

Expand discussion on:
Maternal physiology (vascular, metabolic, autonomic)
Clinical implications for antenatal care
Consider linking to:
Hypertensive disorders
Placental insufficiency pathways

We have expanded the Maternal Health Outcomes section and added a dedicated paragraph discussing maternal outcomes as a major gap in the human literature. The revised manuscript now states that most human studies focused on fetal or neonatal outcomes, while maternal physiological endpoints were rarely assessed. We also link this gap to the animal evidence, noting that animal studies suggest vascular, metabolic and placental mechanisms that could plausibly relate to maternal complications, including endothelial dysfunction, altered arterial stiffness, hypertensive disorders, impaired placental perfusion and gestational metabolic disturbance.

We have also added a recommendation that fut

ure human studies incorporate maternal vascular, autonomic, metabolic and placental endpoints alongside fetal and neonatal outcomes, to assess whether the mechanistic pathways identified in animal studies translate to human pregnancy.

**Location in revised manuscript: Maternal Health Outcomes pp. 26–27; Implications p. 33.**

**Minor Comments
1. Clarity and Language**
Some sentences are overly long and dense.
Minor repetition (e.g., “heterogeneity” appears frequently without added nuance).

Suggestion:

Improve readability by shortening complex sentences.

We have reviewed the manuscript for readability and revised several sections to improve clarity and reduce repetition. In particular, we have revised the Introduction, Discussion and Conclusion to improve flow, reduce unnecessary repetition and ensure that terms such as “heterogeneity” and “variability” are used more precisely. We have also corrected grammatical issues and simplified several longer sentences.

**Location in revised manuscript: throughout the manuscript, particularly the Introduction pp. 5–6 and Discussion/Conclusion pp. 31–34.**

**2. Typographical/Editorial Issues**
Duplicate phrase noted:
“and the and the effects of flavouring constituents”
Ensure consistency:
EV vs EC vs vaping terminology

We have revised the terminology to ensure the consistent use of EV throughout the manuscript to described exclusive vapers, removing the use of EC as we agree this was potentially confusing for readers .

**3. Data Availability Statement**
“Data will be made available upon acceptance”
→ This may not fully align with PLOS ONE open data policy.

Recommendation:

Specify repository + DOI (or intended repository).

The data availability statement has been revised to reflect that all data are now included in the submission as supplementary files.

**Location in revised manuscript: p. 2.**

**4. Figures and Tables**
Tables are comprehensive but dense.

Suggestion:

Consider:
Summary figure (e.g., evidence map)
Visual comparison of human vs animal findings

We agree that the tables are detailed, reflecting the breadth and heterogeneity of the evidence base. We have retained the tables because the purpose of the scoping review is to map the extent, range and nature of the available evidence across both human and animal studies. However, to improve interpretability, we have revised the narrative synthesis to provide clearer summary statements by outcome domain and have added S4 Appendix to present structured methodological appraisal separately. This reduces the amount of methodological detail that must be interpreted from the main tables alone.

We have also created an additional figure (figure 2 P13) which is an evidence map comparing human and animal studies of vaping during pregnancy.

**Location in revised manuscript: evidence-map text and Fig 2 on pp. 12–13; S4 Appendix.**

**5. Ethical Statement**
Marked as “N/A” — appropriate for a review, but ensure:
This is clearly justified in Methods.

We have added an Ethical approval statement to the manuscript, clarifying that ethical approval was not required because this review synthesised data from previously published studies and did not involve collection of individual participant data.

**Location in revised manuscript: p. 35.**

**Reviewer #2:**

This is an important public health topic that warrants study. The review makes a valuable contrubution and is suitable for inclusion in plos one.

The topic is controversial as there is considerable misunderstanding and misinformation about vaping so studies need to be careful about tone and balance. This review is presented very clearly and in my view should not be easily misreported. The approach to the scoping review is sound and results are interpreted clearly and accurately. Conclusions are appropriate and highlight the limitations of the included studies.

I have a few suggestions:

**Pg 5**

Introduction: nicotine-naïve models - needs a few words of explanation of what this means, not all readers will be familar with animal studies.

Thank you. We have added an explanation in the Introduction. The manuscript now explains that nicotine-naïve models are animals with no prior nicotine exposure before experimental gestational exposure.

**Location in revised manuscript: p. 5.**

**Pg 5**

Introduction: Re Reference 20. This study also included heavy metal analysis, that showed these were much lower or not detectable in exclusive vapers compared to smokers. Consider changing this sentence to reflect this: A separate rigorous pregnancy study found exclusive vapers had substantially lower selected VOCs and **heavy metals** than smokers with levels comparable to never-users (20).

We have revised the sentence as suggested. The Introduction now states that a rigorous pregnancy study found exclusive vapers had substantially lower selected VOCs and heavy metals than smokers, with levels comparable to never-users.

**Location in revised manuscript: pp. 5–6.**

**Pg 27**

Human studies - many of these report adjusted odds ratios, but there is no information included about what sort of factors were included in studies adjustments e.g. did these typically include measures of deprivation or socio-economic status, mothers age etc. You do mention inconsistent adjustment for previous smoking, but the level of treatment of other confounders by the included studies could be explored. This may also be relevant to incorporate into the discussion section.

We agree and have expanded the “Methodological characteristics of included human studies” section. We now summarise commonly adjusted variables across studies, including maternal age, race or ethnicity, education, BMI, parity, prenatal care, insurance status, WIC participation, income or area-level deprivation, prior preterm birth and pregnancy complications.

We also explicitly state that adjustment for prior smoking intensity, switching behaviour, dual use, partner or household smoking, alcohol, cannabis and other substance use was inconsistent. This issue is also incorporated into S4 Appendix, which provides a structured study-level appraisal of confounder adjustment and residual confounding.

**Location in revised manuscript: Methodological characteristics pp. 29–31; S4 Appendix.**

**Pg 30:**

You correctly suggest that future studies need to clearly establish smoking and vaping status before conception. I suggest this might also include the smoking status of partners and other household members as second-hand smoking could also be a confounder. Consideration and reporting by future studies of other standard confounding factors: SES, maternal education, age, ethnicity, other risk behaviours etc would also be helpful to include.

We have revised the future research recommendations to address this point. The manuscript now states that smoking and vaping status before conception should be clearly established, with never-users distinguished from current non-users or quitters to avoid residual confounding.

We have also added that future studies should collect partner and household smoking exposure, including second-hand smoke, alongside standard confounders such as socioeconomic status, maternal education, age, ethnicity, BMI, parity, alcohol and cannabis use, other substance use and relevant obstetric history.

**Location in revised manuscript: p. 34.**

Kind Regards,

Dr Stella Fielder

and

Professor Alexander Heazell
