# Supplementary material for: Vaping during pregnancy and maternal, fetal, and infant health outcomes: A scoping review of human and animal studies
Source: PLoS One. 2026 Sep 29;21(9):e0356305. doi: 10.1371/journal.pone.0356305 (PMC13623096; doi:10.1371/journal.pone.0356305)
Supplement: Supplementary file 6 [file pone.0356305.s006.docx]

Maternal & Fetal Health Research Centre

School of Medical Sciences

The University of Manchester

c/o Research Floor

St Mary’s Hospital

Oxford Road,

Manchester	M13 9WL

18^th^ August 2026

Dear Eman Allam,

Below we detail all changes made in the second revision and responses to reviewer comments which required additional modification to the manuscript; the authors responses are shown in blue font. We hope that these amendments address the reviewers’ comments and make the manuscript suitable for publication in PLOS ONE.

**Reviewer #1: Assessment of the previous major comments**

**1. Critical appraisal/interpretability of the human evidence — Addressed, with a small methodological clarification desirable.**

The original concern was that the review made interpretive conclusions without allowing readers to judge differences in study reliability. The authors have now introduced a structured interpretability appraisal covering study design, exposure timing, definitions of exclusive vaping/dual use/non-use, biochemical validation, previous smoking, residual confounding, confounder adjustment and overall interpretability. They appropriately clarify that this is not intended to constitute a formal risk-of-bias score.

The Results also now explicitly direct readers to S4 Appendix and describe what the appraisal contains.

I would therefore regard the original concern as substantially addressed. One minor issue remains: the manuscript states that studies were classified as higher, moderate, moderate-to-lower, or lower interpretability, but the methodology presented in the manuscript does not clearly explain the decision rules used to assign these categories. A brief explanation of how these overall ratings were determined would improve reproducibility and avoid the appearance of subjective grading.

We thank the reviewer for this helpful clarification. We agree that the basis for assigning the overall interpretability categories should be described more explicitly to improve transparency and reproducibility. We have therefore expanded the Materials and methods section to describe the decision-making process used for the structured interpretability appraisal. The revised text now specifies that ratings considered study design and data source, prospective versus retrospective exposure ascertainment, timing and frequency of exposure assessment, exposure-group definitions, biochemical validation, previous smoking and switching behaviour, sample size, confounder adjustment and potential residual confounding.

We have also clarified how these domains informed the overall categories. Higher interpretability reflected stronger methodological features across these domains, while ratings were reduced where several limitations were present, including retrospective self-reported exposure, absence of biochemical validation, small vaping groups, incomplete characterisation of previous smoking or dual use, limited confounder adjustment or substantial residual confounding. We further clarify that categories were assigned holistically rather than using a weighted numerical score and were not intended to constitute a formal risk-of-bias or quality assessment.

**Location in revised manuscript: Materials and methods p. 10 and S4 Appendix.**

**Reviewer #3:**

This study addresses an important and clinically relevant question regarding the role of vaping as a potential smoking-cessation and harm-reduction strategy. Given the growing use of electronic cigarettes and ongoing uncertainty regarding their comparative effectiveness, safety, and clinical applicability, the evidence synthesized in this scoping review is of considerable value to clinicians, researchers, and policymakers.

A few minor issues should nevertheless be considered before publication. First, the limited evidence directly comparing vaping with nicotine replacement therapy (NRT), an established smoking-cessation intervention in several health systems, should be highlighted more explicitly as a key evidence gap because it limits the clinical interpretation of vaping as a harm-reduction option. Second, the rationale for screening the first 100 Google Scholar results should be briefly justified in relation to JBI guidance, with the possibility of missed grey literature, such as conference abstracts and regulatory reports, acknowledged as a limitation. Third, terminology describing the S4 Appendix appraisal should remain clearly distinguished from formal risk-of-bias or quality assessment to avoid misinterpretation. Fourth, the exploratory finding regarding menthol vaping and fetal death (OR 3.27) may warrant a brief cautionary statement in the Abstract, given its magnitude and the potential for selective interpretation. Finally, the clean and tracked-changes versions should be checked to ensure complete consistency in formatting, headings, citation style, and S1–S4 Appendix numbering.

We thank the reviewer for their positive assessment of the manuscript and for these helpful suggestions. We have addressed each of the minor comments as detailed below.

1. *Evidence comparing vaping with NRT*:

We thank the reviewer for this helpful suggestion. We have revised the Discussion to highlight more explicitly the limited evidence directly comparing vaping with NRT as a key evidence gap, and to clarify that this limits conclusions regarding the relative safety and clinical role of vaping compared with established smoking-cessation strategies in pregnancy.

**Location in revised manuscript: Discussion p. 32.**

2. *Rationale for screening the first 100 Google Scholar results*:

We have clarified in the Methods section that screening the first 100 Google Scholar results was undertaken as a pragmatic supplementary search informed by JBI guidance recommending review of the first 5–10 pages of supplementary Google search results. We have also added to the limitations that restricting screening to the first 100 results may have resulted in relevant grey literature, including conference abstracts or regulatory reports, being missed.

**Location in revised manuscript: Materials and methods p. 8, Limitations p. 34.**

3. *Terminology describing the S4 Appendix appraisal*:

We thank the reviewer for this helpful comment. We have ensured that the terminology used throughout the manuscript and S4 Appendix consistently describes this as a structured interpretability appraisal, and we have explicitly clarified in the Methods that it was not intended to constitute a formal risk-of-bias or quality assessment.

**Location in revised manuscript: Materials and methods p. 10 and S4 Appendix.**

4. *The exploratory finding regarding menthol vaping and fetal death*:

We thank the reviewer for this comment. The exploratory association between mint/menthol-flavoured vaping and fetal death is not reported in the Abstract, which presents the overall findings of the scoping review rather than individual exploratory associations. We have therefore not introduced this finding into the Abstract, as doing so could give disproportionate prominence to a small subgroup analysis. In the Discussion, where the finding is reported, we have retained explicit cautionary language noting the small subgroup size, limited adjustment for confounding, wide confidence intervals, and the need for cautious interpretation.

**Location in revised manuscript: Discussion p. 32.**

5. *Clean and tracked-changes versions: consistency in formatting, headings, citation style, and S1–S4 Appendix numbering:*

We thank the reviewer for this comment. We have checked the clean and tracked-changes versions to ensure consistency in formatting, headings, citation style, and S1–S4 Appendix numbering and corrected minor inconsistencies where identified.

Kind Regards,

Dr Stella Fielder and Professor Alexander Heazell
