# Supplementary material for: Vaping during pregnancy and maternal, fetal, and infant health outcomes: A scoping review of human and animal studies
Source: PLoS One. 2026 Sep 29;21(9):e0356305. doi: 10.1371/journal.pone.0356305 (PMC13623096; doi:10.1371/journal.pone.0356305)
Supplement: Peer review history

# Peer review: Decision Letter 0

**DOI:** 10.1371/journal.pone.0356305.r001

**Authors:** Eman Allam

24 May 2026

Dear Dr. Fielder,

Thank you for submitting your manuscript to PLOS ONE. After careful consideration, we feel that it has merit but does not fully meet PLOS ONE’s publication criteria as it currently stands. Therefore, we invite you to submit a revised version of the manuscript that addresses the points raised during the review process.

==============================

Please submit your revised manuscript by Jul 09 2026 11:59PM. If you will need more time than this to complete your revisions, please reply to this message or contact the journal office at plosone@plos.org. When you're ready to submit your revision, log on to https://www.editorialmanager.com/pone/ and select the 'Submissions Needing Revision' folder to locate your manuscript file.

- A letter that responds to each point raised by the academic editor and reviewer(s). You should upload this letter as a separate file labeled 'Response to Reviewers'.
- A marked-up copy of your manuscript that highlights changes made to the original version. You should upload this as a separate file labeled 'Revised Manuscript with Track Changes'.
- An unmarked version of your revised paper without tracked changes. You should upload this as a separate file labeled 'Manuscript'.

If you would like to make changes to your financial disclosure, please include your updated statement in your cover letter. Guidelines for resubmitting your figure files are available below the reviewer comments at the end of this letter.

If applicable, we recommend that you deposit your laboratory protocols in protocols.io to enhance the reproducibility of your results. Protocols.io assigns your protocol its own identifier (DOI) so that it can be cited independently in the future. For instructions see: https://journals.plos.org/plosone/s/submission-guidelines#loc-laboratory-protocols. Additionally, PLOS ONE offers an option for publishing peer-reviewed Lab Protocol articles, which describe protocols hosted on protocols.io. Read more information on sharing protocols at https://plos.org/protocols?utm_medium=editorial-email&utm_source=authorletters&utm_campaign=protocols.

As the corresponding author, your ORCID iD is verified in the submission system and will appear in the published article. PLOS supports the use of ORCID, and we encourage all coauthors to register for an ORCID iD and use it as well. Please encourage your coauthors to verify their ORCID iD within the submission system before final acceptance, as unverified ORCID iDs will not appear in the published article. *Only*  the individual author can complete the verification step; PLOS staff *cannot*  verify ORCID iDs on behalf of authors.

We look forward to receiving your revised manuscript.

Kind regards,

Eman Allam, PhD

Academic Editor

PLOS One

Journal requirements:

When submitting your revision, we need you to address these additional requirements.

1. Please ensure that your manuscript meets PLOS ONE's style requirements, including those for file naming. The PLOS ONE style templates can be found at

https://journals.plos.org/plosone/s/file?id=wjVg/PLOSOne_formatting_sample_main_body.pdf and

https://journals.plos.org/plosone/s/file?id=ba62/PLOSOne_formatting_sample_title_authors_affiliations.pdf

2. When completing the data availability statement of the submission form, you indicated that you will make your data available on acceptance. We strongly recommend all authors decide on a data sharing plan before acceptance, as the process can be lengthy and hold up publication timelines. Please note that, though access restrictions are acceptable now, your entire data will need to be made freely accessible if your manuscript is accepted for publication. This policy applies to all data except where public deposition would breach compliance with the protocol approved by your research ethics board. If you are unable to adhere to our open data policy, please kindly revise your statement to explain your reasoning and we will seek the editor's input on an exemption. Please be assured that, once you have provided your new statement, the assessment of your exemption will not hold up the peer review process.

3. Please include captions for your Supporting Information files at the end of your manuscript, and update any in-text citations to match accordingly. Please see our Supporting Information guidelines for more information: http://journals.plos.org/plosone/s/supporting-information.

If the reviewer comments include a recommendation to cite specific previously published works, please review and evaluate these publications to determine whether they are relevant and should be cited. There is no requirement to cite these works unless the editor has indicated otherwise.

[Note: HTML markup is below. Please do not edit.]

Reviewers' comments:

Reviewer's Responses to Questions

**Comments to the Author**

1. Is the manuscript technically sound, and do the data support the conclusions?

Reviewer #1: Partly

Reviewer #2: Yes

**********

2. Has the statistical analysis been performed appropriately and rigorously?

Reviewer #1: No

Reviewer #2: Yes

**********

3. Have the authors made all data underlying the findings in their manuscript fully available?

The PLOS Data policy

Reviewer #1: Yes

Reviewer #2: Yes

**********

4. Is the manuscript presented in an intelligible fashion and written in standard English?

Reviewer #1: Yes

Reviewer #2: Yes

**********

Reviewer #1: General Assessment

This manuscript presents a timely and important scoping review addressing the growing public health concern of vaping during pregnancy. The inclusion of both human and animal studies is a notable strength, allowing exploration of translational gaps between mechanistic and clinical evidence. The study is generally well-structured and follows JBI and PRISMA-ScR guidance, with a clearly stated objective and comprehensive search strategy.

However, while the manuscript successfully maps the literature, there are important methodological, conceptual, and reporting issues that need to be addressed to strengthen rigor, interpretability, and contribution to the field.

Major Comments

1. Lack of Critical Appraisal Limits Interpretability

Although scoping reviews do not require formal risk-of-bias assessment, the manuscript goes beyond mapping and makes interpretive statements (e.g., “insufficient to draw firm conclusions”).

Without at least a basic quality stratification, readers cannot judge:

Strength of evidence

Reliability of associations (especially PRAMS vs prospective cohorts)

Recommendation:

Include a structured appraisal (even simplified) such as:

Study design hierarchy

Exposure validity (biochemical vs self-report)

Confounding control

Alternatively, explicitly limit interpretive language and remain descriptive.

2. Over-reliance on PRAMS Data Not Adequately Problematised

The manuscript identifies that many findings come from PRAMS datasets but does not fully interrogate this bias.

Key issue:

PRAMS = retrospective self-report + recall bias + misclassification

Multiple studies using the same dataset may give false impression of evidence volume

Recommendation:

Add a dedicated subsection on:

Data source clustering

Risk of duplication bias

Quantify proportion of findings derived from PRAMS vs independent datasets.

3. Exposure Classification is a Critical Weakness but Not Fully Analysed

The manuscript repeatedly notes heterogeneity in:

Exclusive vaping (EV)

Dual use

Smoking history

However, this is not systematically synthesised.

Problem:

Misclassification (e.g., “exclusive vapers” who are ex-smokers) can inflate risk estimates

Recommendation:

Create a table or framework summarising:

How EV, dual use, and non-use were defined across studies

Explicitly discuss:

Residual confounding from prior smoking

Timing of exposure (pre-pregnancy vs during pregnancy)

4. Combining Human and Animal Evidence Needs Stronger Conceptual Framing

The inclusion of animal studies is valuable but currently feels parallel rather than integrated.

Issue:

Limited synthesis of:

How mechanistic findings relate to human outcomes

What can/cannot be translated

Recommendation:

Add a dedicated “Translational Interpretation” section:

Which pathways (e.g., oxidative stress, endothelial dysfunction) are plausible in humans

Which findings are likely artefacts of high-dose models

5. Conclusions Slightly Overreach the Evidence

The statement:

“current literature is insufficient to draw firm conclusions regarding risk”

is reasonable, but elsewhere the manuscript implies potential harm based on animal data.

Issue:

Risk of implicit causal inference from heterogeneous and low-quality evidence

Recommendation:

Clearly separate:

Evidence gaps

Hypothesised risks

Use more cautious phrasing:

“suggestive but inconclusive”

“biologically plausible but not confirmed in humans”

6. Maternal Outcomes Are Underdeveloped

The review highlights limited maternal data but does not deeply analyse it.

Given your expertise in maternal health, this is a missed opportunity.

Recommendation:

Expand discussion on:

Maternal physiology (vascular, metabolic, autonomic)

Clinical implications for antenatal care

Consider linking to:

Hypertensive disorders

Placental insufficiency pathways

Minor Comments

1. Clarity and Language

Some sentences are overly long and dense.

Minor repetition (e.g., “heterogeneity” appears frequently without added nuance).

Suggestion:

Improve readability by shortening complex sentences.

2. Typographical/Editorial Issues

Duplicate phrase noted:

“and the and the effects of flavouring constituents”

Ensure consistency:

EV vs EC vs vaping terminology

3. Data Availability Statement

“Data will be made available upon acceptance”

→ This may not fully align with PLOS ONE open data policy.

Recommendation:

Specify repository + DOI (or intended repository).

4. Figures and Tables

Tables are comprehensive but dense.

Suggestion:

Consider:

Summary figure (e.g., evidence map)

Visual comparison of human vs animal findings

5. Ethical Statement

Marked as “N/A” — appropriate for a review, but ensure:

This is clearly justified in Methods.

Strengths of the Manuscript

Comprehensive search across multiple databases

Inclusion of both human and animal evidence

Clear identification of:

Exposure misclassification

Lack of biochemical validation

Strong alignment with PRISMA-ScR methodology

Clinically relevant topic with policy implications

Recommendation

Major Revision

The manuscript has strong potential but requires:

Deeper methodological critique

Improved synthesis (not just mapping)

Clearer handling of bias and heterogeneity

Stronger translational interpretation

Reviewer #2: This is an important public health topic that warrants study. The review makes a valuable contrubution and is suitable for inclusion in plos one.

The topic is controversial as there is considerable misunderstanding and misinformation about vaping so studies need to be careful about tone and balance. This review is presented very clearly and in my view should not be easily misreported. The approach to the scoping review is sound and results are interpreted clearly and accurately. Conclusions are appropriate and highlight the limitations of the included studies.

I have a few suggestions:

Pg 5 Introduction: nicotine-naïve models - needs a few words of explanation of what this means, not all readers will be familar with animal studies.

Pg 5 Introduction: Re Reference 20. This study also included heavy metal analysis, that showed these were much lower or not detectable in exclusive vapers compared to smokers. Consider changing this sentence to reflect this:

A separate rigorous pregnancy study found exclusive vapers had substantially lower selected VOCs and **heavy metals** than smokers with levels comparable to never-users (20).

Pg 27 Human studies - many of these report adjusted odds ratios, but there is no information included about what sort of factors were included in studies adjustments e.g did these typically include measures of deprivation or socio-economic status, mothers age etc. You do mention inconsistent adjustment for previous smoking, but the level of treatment of other confounders by the included studies could be explored. This may also be relevant to incorporate into the discussion section.

Pg 30: You correctly suggest that future studies need to clearly establish smoking and vaping status before conception. I suggest this might also include the smoking status of partners and other household members as second-hand smoking could also be a confounder. Consideration and reporting by future studies of other standard confounding factors: SES, maternal education, age, ethnicity, other risk behaviours etc would also be helpful to include.

**********

what does this mean?). If published, this will include your full peer review and any attached files.

If you choose “no”, your identity will remain anonymous but your review may still be made public.

**Do you want your identity to be public for this peer review?**  For information about this choice, including consent withdrawal, please see our Privacy Policy

Reviewer #1: **Yes:**  JOHN KWAME AGBENYEAVU

Reviewer #2: **Yes:**  Dr Ivan L Gee

**********

[NOTE: If reviewer comments were submitted as an attachment file, they will be attached to this email and accessible via the submission site. Please log into your account, locate the manuscript record, and check for the action link "View Attachments". If this link does not appear, there are no attachment files.]

To ensure your figures meet our technical requirements, please review our figure guidelines: https://journals.plos.org/plosone/s/figures

You may also use PLOS’s free figure tool, NAAS, to help you prepare publication quality figures: https://journals.plos.org/plosone/s/figures#loc-tools-for-figure-preparation.

NAAS will assess whether your figures meet our technical requirements by comparing each figure against our figure specifications.

# Author comment: Author response to Decision Letter 1

**DOI:** 10.1371/journal.pone.0356305.r002

3 Aug 2026

Below we detail all changes made in the revision and responses to all reviewer comments. We hope that these amendments make the manuscript suitable for publication in PLOS ONE.

Journal requirements:

When submitting your revision, we need you to address these additional requirements.

1. Please ensure that your manuscript meets PLOS ONE's style requirements, including those for file naming. The PLOS ONE style templates can be found at

https://journals.plos.org/plosone/s/file?id=wjVg/PLOSOne_formatting_sample_main_body.pdf [journals.plos.org] and

https://journals.plos.org/plosone/s/file?id=ba62/PLOSOne_formatting_sample_title_authors_affiliations.pdf [journals.plos.org]

We have revised the manuscript to align with PLOS ONE formatting requirements. In particular, we have changed the “Methods” heading to “Materials and methods”, standardised figure and table captions, updated in-text citations to PLOS style, and ensured that Supporting Information files are cited as S1 Appendix, S2 Appendix, S3 Appendix and S4 Appendix. We have prepared both a clean manuscript and a tracked-changes version for submission, as requested.

Location in revised manuscript: throughout the revised manuscript (pp. 3–46).

2. When completing the data availability statement of the submission form, you indicated that you will make your data available on acceptance. We strongly recommend all authors decide on a data sharing plan before acceptance, as the process can be lengthy and hold up publication timelines. Please note that, though access restrictions are acceptable now, your entire data will need to be made freely accessible if your manuscript is accepted for publication. This policy applies to all data except where public deposition would breach compliance with the protocol approved by your research ethics board. If you are unable to adhere to our open data policy, please kindly revise your statement to explain your reasoning and we will seek the editor's input on an exemption. Please be assured that, once you have provided your new statement, the assessment of your exemption will not hold up the peer review process.

We have revised the data availability statement to clarify that all data underlying the findings of this review are available within the manuscript and Supporting Information files. These include the full search strategies, PRISMA-ScR checklist, extracted study characteristics and outcomes, and structured study appraisal and exposure classification. As this is a scoping review, no individual participant data were generated or analysed by the authors.

The revised statement reads:

“All data underlying the findings of this review are available within the manuscript and Supporting Information files. These include the full search strategies, PRISMA-ScR checklist, extracted study characteristics and outcomes, and structured study appraisal and exposure classification. No individual participant data were generated or analysed by the authors for this review.”

We have also added a code availability statement clarifying that no custom code was used beyond spreadsheet formulas to aggregate counts and produce tables.

Location in revised manuscript: p. 2.

3. Please include captions for your Supporting Information files at the end of your manuscript, and update any in-text citations to match accordingly. Please see our Supporting Information guidelines for more information: http://journals.plos.org/plosone/s/supporting-information [journals.plos.org].

We have added a Supporting Information section at the end of the manuscript with captions for S1–S4 Appendices. We have also updated the in-text citations to match PLOS ONE Supporting Information style.

Location in revised manuscript: Supporting Information captions on p. 35

Reviewers' comments:

Comments to the Author

Reviewer #1: General Assessment

This manuscript presents a timely and important scoping review addressing the growing public health concern of vaping during pregnancy. The inclusion of both human and animal studies is a notable strength, allowing exploration of translational gaps between mechanistic and clinical evidence. The study is generally well-structured and follows JBI and PRISMA-ScR guidance, with a clearly stated objective and comprehensive search strategy

However, while the manuscript successfully maps the literature, there are important methodological, conceptual, and reporting issues that need to be addressed to strengthen rigor, interpretability, and contribution to the field.

We thank the reviewer for this helpful assessment and for recognising the importance of the topic, the value of combining human and animal evidence. We have made multiple revisions throughout the manuscript in line with their recommendations as detailed below.

Location in revised manuscript: revisions throughout pp. 3–37.

Major Comments

1. Lack of Critical Appraisal Limits Interpretability

Although scoping reviews do not require formal risk-of-bias assessment, the manuscript goes beyond mapping and makes interpretive statements (e.g., “insufficient to draw firm conclusions”).

Without at least a basic quality stratification, readers cannot judge:

Strength of evidence

Reliability of associations (especially PRAMS vs prospective cohorts)

Recommendation:

Include a structured appraisal (even simplified) such as:

Study design hierarchy

Exposure validity (biochemical vs self-report)

Confounding control

Alternatively, explicitly limit interpretive language and remain descriptive.

We agree that, although formal risk-of-bias assessment is not required for scoping reviews, additional structured appraisal would help readers interpret the evidence. We have therefore added a structured interpretability appraisal of the included human studies, presented as S4 Appendix.

This appraisal includes study design and data source, exposure timing, definitions of exclusive vaping, dual use and non-use, biochemical validation, whether previous smoking or dual use was addressed, residual confounding from previous or concurrent combustible cigarette use, confounder adjustment, the main interpretability limitation and an overall descriptive interpretability rating.

We have also added explanatory text in the Materials and methods section to clarify the rationale for this approach. The revised manuscript states that the appraisal was not intended to produce a formal risk-of-bias score, but to map the methodological features most relevant to interpreting associations between vaping and pregnancy outcomes.

We have also added a signposting sentence in the Results section and expanded the “Methodological characteristics of included human studies” section to summarise the findings of this appraisal. Finally, we revised interpretive language in the Abstract, Discussion and Conclusion to ensure that conclusions remain cautious and proportionate to the evidence base.

Location in revised manuscript: Materials and methods p. 10; Results p. 12; Methodological characteristics pp. 29–31; Abstract pp. 3–4; Discussion pp. 31–34; Conclusion pp. 35–37; and S4 Appendix.

2. Over-reliance on PRAMS Data Not Adequately Problematised

The manuscript identifies that many findings come from PRAMS datasets but does not fully interrogate this bias.

Key issue:

PRAMS = retrospective self-report + recall bias + misclassification

Multiple studies using the same dataset may give false impression of evidence volume

Recommendation:

Add a dedicated subsection on:

Data source clustering

Risk of duplication bias

Quantify proportion of findings derived from PRAMS vs independent datasets.

We have added a dedicated paragraph in the Discussion addressing data-source clustering. The revised Discussion explains that repeated analyses of overlapping PRAMS years should not be interpreted as fully independent evidence because the same underlying limitations may be reproduced across multiple publications. These limitations include reliance on postpartum recall, self-reported smoking and vaping status, limited detail on vaping patterns, incomplete capture of earlier pregnancy smoking and absence of biochemical validation.

We also now state that this clustering may give an inflated impression of evidence volume, particularly for low birth weight, preterm birth, small for gestational age, postpartum depression and gestational weight gain outcomes. This revision directly addresses the concern that repeated analyses of the same data source may affect interpretation of the apparent consistency or volume of evidence.

Location in revised manuscript: Discussion pp. 31–32.

3. Exposure Classification is a Critical Weakness but Not Fully Analysed

The manuscript repeatedly notes heterogeneity in:

Exclusive vaping (EV)

Dual use

Smoking history

However, this is not systematically synthesised.

Problem:

Misclassification (e.g., “exclusive vapers” who are ex-smokers) can inflate risk estimates

Recommendation:

Create a table or framework summarising:

How EV, dual use, and non-use were defined across studies

Explicitly discuss:

Residual confounding from prior smoking

Timing of exposure (pre-pregnancy vs during pregnancy)

Thank you. We have addressed this through the addition of the table in S4 Appendix and through expanded synthesis in the main manuscript.

S4 Appendix provides a structured study-level framework summarising how exclusive vaping, dual use and non-use were defined across included human studies. It also records exposure timing, whether biochemical validation was used, whether previous smoking or dual use was addressed, residual confounding from previous or concurrent combustible cigarette use, confounder adjustment and overall interpretability.

In the Results section, we have expanded the “Methodological characteristics of included human studies” subsection to summarise these issues. The revised manuscript now explains that in several PRAMS analyses, exclusive vaping referred to self-reported e-cigarette use without concurrent combustible cigarette smoking during the last three months of pregnancy, rather than lifelong exclusive vaping or biochemically confirmed abstinence from tobacco. We also discuss how some studies included former smokers, quitters or participants with unclear previous smoking history within comparator groups, and that non-use was inconsistently defined across studies.

We have also expanded the Discussion to emphasise that exposure misclassification and residual confounding from prior smoking limit interpretation of apparent associations, particularly where studies classified participants using late-pregnancy exposure only.

Location in revised manuscript: Methodological characteristics pp. 29–30; Discussion pp. 32–34; and S4 Appendix.

4. Combining Human and Animal Evidence Needs Stronger Conceptual Framing

The inclusion of animal studies is valuable but currently feels parallel rather than integrated.

Issue:

Limited synthesis of:

How mechanistic findings relate to human outcomes

What can/cannot be translated

Recommendation:

Add a dedicated “Translational Interpretation” section:

Which pathways (e.g., oxidative stress, endothelial dysfunction) are plausible in humans

Which findings are likely artefacts of high-dose models

We agree and have substantially revised the Discussion to improve animal-human translational framing. We now explicitly discuss mechanistic pathways identified in animal studies, including oxidative stress, inflammation, endothelial dysfunction, altered placental perfusion, metabolic dysregulation, epigenetic modification and neurodevelopmental disruption. We then link these pathways to clinically relevant human outcomes, including fetal growth restriction, hypertensive disorders, placental insufficiency, respiratory morbidity and altered neurodevelopment.

We have also added clearer caution around translation. The revised Discussion states that animal findings should be interpreted as mechanistic and hypothesis-generating rather than confirmatory evidence of clinical risk in humans. We specifically identify limitations including physiological differences, species-specific placental structure, differences in nicotine metabolism, nicotine-naïve dams and exposure paradigms involving continuous or high-concentration aerosol exposure that may not reflect typical human use.

Location in revised manuscript: Evidence map pp. 12–13; Discussion pp. 31–32.

5. Conclusions Slightly Overreach the Evidence

The statement:

“current literature is insufficient to draw firm conclusions regarding risk”

is reasonable, but elsewhere the manuscript implies potential harm based on animal data.

Issue:

Risk of implicit causal inference from heterogeneous and low-quality evidence

Recommendation:

Clearly separate:

Evidence gaps

Hypothesised risks

Use more cautious phrasing:

“suggestive but inconclusive”

“biologically plausible but not confirmed in humans”

We have revised the Abstract, Discussion and Conclusion to ensure that conclusions are cautious and do not imply causal inference from heterogeneous evidence. The revised manuscript now states that animal studies identify biologically plausible mechanisms of harm, but that these findings remain hypothesis-generating and have not been consistently confirmed in human pregnancy studies. We have also clarified that direct translation is limited by physiological differences, species-specific placental structure, differences in nicotine metabolism and exposure paradigms that may not reflect typical human use.

We have revised comparative statements to avoid overstatement. For example, the Discussion now states that although several studies reported lower levels of association for exclusive vaping compared with combustible cigarette use across low birth weight and preterm birth outcomes, confidence intervals frequently overlapped and exposure misclassification limits firm conclusions. We also state that absence of evidence should not be interpreted as evidence of safety.

Location in revised manuscript: Abstract pp. 3–4; Discussion pp. 31–33; Conclusion pp. 34.

6. Maternal Outcomes Are Underdeveloped

The review highlights limited maternal data but does not deeply analyse it.

Given your expertise in maternal health, this is a missed opportunity.

Recommendation:

Expand discussion on:

Maternal physiology (vascular, metabolic, autonomic)

Clinical implications for antenatal care

Consider linking to:

Hypertensive disorders

Placental insufficiency pathways

We have expanded the Maternal Health Outcomes section and added a dedicated paragraph discussing maternal outcomes as a major gap in the human literature. The revised manuscript now states that most human studies focused on fetal or neonatal outcomes, while maternal physiological endpoints were rarely assessed. We also link this gap to the animal evidence, noting that animal studies suggest vascular, metabolic and placental mechanisms that could plausibly relate to maternal complications, including endothelial dysfunction, altered arterial stiffness, hypertensive disorders, impaired placental perfusion and gestational metabolic disturbance.

We have also added a recommendation that fut

ure human studies incorporate maternal vascular, autonomic, metabolic and placental endpoints alongside fetal and neonatal outcomes, to assess whether the mechanistic pathways identified in animal studies translate to human pregnancy.

Location in revised manuscript: Maternal Health Outcomes pp. 26–27; Implications p. 33.

Minor Comments

1. Clarity and Language

Some sentences are overly long and dense.

Minor repetition (e.g., “heterogeneity” appears frequently without added nuance).

Suggestion:

Improve readability by shortening complex sentences.

We have reviewed the manuscript for readability and revised several sections to improve clarity and reduce repetition. In particular, we have revised the Introduction, Discussion and Conclusion to improve flow, reduce unnecessary repetition and ensure that terms such as “heterogeneity” and “variability” are used more precisely. We have also corrected grammatical issues and simplified several longer sentences.

Location in revised manuscript: throughout the manuscript, particularly the Introduction pp. 5–6 and Discussion/Conclusion pp. 31–34.

2. Typographical/Editorial Issues

Duplicate phrase noted:

“and the and the effects of flavouring constituents”

Ensure consistency:

EV vs EC vs vaping terminology

We have revised the terminology to ensure the consistent use of EV throughout the manuscript to described exclusive vapers, removing the use of EC as we agree this was potentially confusing for readers .

3. Data Availability Statement

“Data will be made available upon acceptance”

→ This may not fully align with PLOS ONE open data policy.

Recommendation:

Specify repository + DOI (or intended repository).

The data availability statement has been revised to reflect that all data are now included in the submission as supplementary files.

Location in revised manuscript: p. 2.

4. Figures and Tables

Tables are comprehensive but dense.

Suggestion:

Consider:

Summary figure (e.g., evidence map)

Visual comparison of human vs animal findings

We agree that the tables are detailed, reflecting the breadth and heterogeneity of the evidence base. We have retained the tables because the purpose of the scoping review is to map the extent, range and nature of the available evidence across both human and animal studies. However, to improve interpretability, we have revised the narrative synthesis to provide clearer summary statements by outcome domain and have added S4 Appendix to present structured methodological appraisal separately. This reduces the amount of methodological detail that must be interpreted from the main tables alone.

We have also created an additional figure (figure 2 P13) which is an evidence map comparing human and animal studies of vaping during pregnancy.

Location in revised manuscript: evidence-map text and Fig 2 on pp. 12–13; S4 Appendix.

5. Ethical Statement

Marked as “N/A” — appropriate for a review, but ensure:

This is clearly justified in Methods.

We have added an Ethical approval statement to the manuscript, clarifying that ethical approval was not required because this review synthesised data from previously published studies and did not involve collection of individual participant data.

Location in revised manuscript: p. 35.

Reviewer #2:

This is an important public health topic that warrants study. The review makes a valuable contrubution and is suitable for inclusion in plos one.

The topic is controversial as there is considerable misunderstanding and misinformation about vaping so studies need to be careful about tone and balance. This review is presented very clearly and in my view should not be easily misreported. The approach to the scoping review is sound and results are interpreted clearly and accurately. Conclusions are appropriate and highlight the limitations of the included studies.

I have a few suggestions:

Pg 5

Introduction: nicotine-naïve models - needs a few words of explanation of what this means, not all readers will be familar with animal studies.

Thank you. We have added an explanation in the Introduction. The manuscript now explains that nicotine-naïve models are animals with no prior nicotine exposure before experimental gestational exposure.

Location in revised manuscript: p. 5.

Pg 5

Introduction: Re Reference 20. This study also included heavy metal analysis, that showed these were much lower or not detectable in exclusive vapers compared to smokers. Consider changing this sentence to reflect this: A separate rigorous pregnancy study found exclusive vapers had substantially lower selected VOCs and **heavy metals** than smokers with levels comparable to never-users (20).

We have revised the sentence as suggested. The Introduction now states that a rigorous pregnancy study found exclusive vapers had substantially lower selected VOCs and heavy metals than smokers, with levels comparable to never-users.

Location in revised manuscript: pp. 5–6.

Pg 27

Human studies - many of these report adjusted odds ratios, but there is no information included about what sort of factors were included in studies adjustments e.g. did these typically include measures of deprivation or socio-economic status, mothers age etc. You do mention inconsistent adjustment for previous smoking, but the level of treatment of other confounders by the included studies could be explored. This may also be relevant to incorporate into the discussion section.

We agree and have expanded the “Methodological characteristics of included human studies” section. We now summarise commonly adjusted variables across studies, including maternal age, race or ethnicity, education, BMI, parity, prenatal care, insurance status, WIC participation, income or area-level deprivation, prior preterm birth and pregnancy complications.

We also explicitly state that adjustment for prior smoking intensity, switching behaviour, dual use, partner or household smoking, alcohol, cannabis and other substance use was inconsistent. This issue is also incorporated into S4 Appendix, which provides a structured study-level appraisal of confounder adjustment and residual confounding.

Location in revised manuscript: Methodological characteristics pp. 29–31; S4 Appendix.

Pg 30:

You correctly suggest that future studies need to clearly establish smoking and vaping status before conception. I suggest this might also include the smoking status of partners and other household members as second-hand smoking could also be a confounder. Consideration and reporting by future studies of other standard confounding factors: SES, maternal education, age, ethnicity, other risk behaviours etc would also be helpful to include.

We have revised the future research recommendations to address this point. The manuscript now states that smoking and vaping status before conception should be clearly established, with never-users distinguished from current non-users or quitters to avoid residual confounding.

We have also added that future studies should collect partner and household smoking exposure, including second-hand smoke, alongside standard confounders such as socioeconomic status, maternal education, age, ethnicity, BMI, parity, alcohol and cannabis use, other substance use and relevant obstetric history.

Location in revised manuscript: p. 34.

Attachment Submitted filename: PLOS one response to reviewer comments.docx

# Peer review: Decision Letter 1

**DOI:** 10.1371/journal.pone.0356305.r003

**Authors:** Eman Allam

17 Aug 2026

Dear Dr. Fielder,

Thank you for submitting your manuscript to PLOS ONE. After careful consideration, we feel that it has merit but does not fully meet PLOS ONE’s publication criteria as it currently stands. Therefore, we invite you to submit a revised version of the manuscript that addresses the points raised during the review process.

Please submit your revised manuscript by Oct 01 2026 11:59PM. If you will need more time than this to complete your revisions, please reply to this message or contact the journal office at plosone@plos.org. When you're ready to submit your revision, log on to https://www.editorialmanager.com/pone/ and select the 'Submissions Needing Revision' folder to locate your manuscript file.

- A letter that responds to each point raised by the academic editor and reviewer(s). You should upload this letter as a separate file labeled 'Response to Reviewers'.
- A marked-up copy of your manuscript that highlights changes made to the original version. You should upload this as a separate file labeled 'Revised Manuscript with Track Changes'.
- An unmarked version of your revised paper without tracked changes. You should upload this as a separate file labeled 'Manuscript'.

If you would like to make changes to your financial disclosure, please include your updated statement in your cover letter. Guidelines for resubmitting your figure files are available below the reviewer comments at the end of this letter.

If applicable, we recommend that you deposit your laboratory protocols in protocols.io to enhance the reproducibility of your results. Protocols.io assigns your protocol its own identifier (DOI) so that it can be cited independently in the future. For instructions see: https://journals.plos.org/plosone/s/submission-guidelines#loc-laboratory-protocols. Additionally, PLOS ONE offers an option for publishing peer-reviewed Lab Protocol articles, which describe protocols hosted on protocols.io. Read more information on sharing protocols at https://plos.org/protocols?utm_medium=editorial-email&utm_source=authorletters&utm_campaign=protocols.

As the corresponding author, your ORCID iD is verified in the submission system and will appear in the published article. PLOS supports the use of ORCID, and we encourage all coauthors to register for an ORCID iD and use it as well. Please encourage your coauthors to verify their ORCID iD within the submission system before final acceptance, as unverified ORCID iDs will not appear in the published article. *Only*  the individual author can complete the verification step; PLOS staff *cannot*  verify ORCID iDs on behalf of authors.

We look forward to receiving your revised manuscript.

Kind regards,

Eman Allam, PhD

Academic Editor

PLOS One

Journal Requirements:

1. If the reviewer comments include a recommendation to cite specific previously published works, please review and evaluate these publications to determine whether they are relevant and should be cited. There is no requirement to cite these works unless the editor has indicated otherwise.

2. Please review your reference list to ensure that it is complete and correct. If you have cited papers that have been retracted, please include the rationale for doing so in the manuscript text, or remove these references and replace them with relevant current references. Any changes to the reference list should be mentioned in the rebuttal letter that accompanies your revised manuscript. If you need to cite a retracted article, indicate the article’s retracted status in the References list and also include a citation and full reference for the retraction notice.

[Note: HTML markup is below. Please do not edit.]

Reviewers' comments:

Reviewer's Responses to Questions

**Comments to the Author**

Reviewer #1: All comments have been addressed

Reviewer #3: (No Response)

**********

2. Is the manuscript technically sound, and do the data support the conclusions?

Reviewer #1: Yes

Reviewer #3: Yes

**********

3. Has the statistical analysis been performed appropriately and rigorously?

Reviewer #1: Yes

Reviewer #3: Yes

**********

4. Have the authors made all data underlying the findings in their manuscript fully available?

The PLOS Data policy

Reviewer #1: Yes

Reviewer #3: Yes

**********

5. Is the manuscript presented in an intelligible fashion and written in standard English?

Reviewer #1: Yes

Reviewer #3: Yes

**********

Reviewer #1: Assessment of the previous major comments

1. Critical appraisal/interpretability of the human evidence — Addressed, with a small methodological clarification desirable.

The original concern was that the review made interpretive conclusions without allowing readers to judge differences in study reliability. The authors have now introduced a structured interpretability appraisal covering study design, exposure timing, definitions of exclusive vaping/dual use/non-use, biochemical validation, previous smoking, residual confounding, confounder adjustment and overall interpretability. They appropriately clarify that this is not intended to constitute a formal risk-of-bias score.

The Results also now explicitly direct readers to S4 Appendix and describe what the appraisal contains.

I would therefore regard the original concern as substantially addressed. One minor issue remains: the manuscript states that studies were classified as higher, moderate, moderate-to-lower, or lower interpretability, but the methodology presented in the manuscript does not clearly explain the decision rules used to assign these categories. A brief explanation of how these overall ratings were determined would improve reproducibility and avoid the appearance of subjective grading.

2. Over-reliance on PRAMS data — Fully addressed.

This is one of the strongest improvements. The revised Discussion now quantifies the problem: 18 of the 30 human studies (60%) were secondary analyses of PRAMS data. The authors appropriately explain that analyses involving overlapping PRAMS years should not be considered completely independent evidence and describe how recall bias, self-reported exposure, incomplete smoking histories and absence of biochemical verification may be reproduced across publications.

This directly addresses the earlier concern that multiple publications based on the same source could exaggerate the apparent volume or consistency of evidence.

3. Exposure classification, previous smoking and residual confounding — Fully addressed.

The revision gives substantially greater attention to exposure misclassification. The manuscript now distinguishes between exclusive vaping, dual use and non-use and explains that some PRAMS classifications of “exclusive vaping” actually referred to absence of concurrent combustible-cigarette smoking during the final three months of pregnancy, rather than lifelong exclusive vaping or biochemically verified tobacco abstinence.

It also discusses variation in adjustment for maternal age, ethnicity, education, BMI, parity, prenatal care, socioeconomic indicators and obstetric factors, while noting inconsistent control for previous smoking intensity, switching behaviour, household smoking, alcohol, cannabis and other substance use.

This substantially improves interpretation of the reported adjusted associations.

4. Integration of human and animal evidence — Addressed.

The authors have strengthened the translational interpretation considerably. The revised Discussion links animal findings involving oxidative stress, inflammation, endothelial dysfunction, altered placental perfusion, metabolic dysregulation, epigenetic changes and neurodevelopmental effects with clinically relevant human outcomes.

Importantly, the authors now explicitly state that animal evidence should be viewed as mechanistic and hypothesis-generating rather than confirmation of clinical risk in humans. They also acknowledge species differences, placental differences, nicotine metabolism and the use of continuous or high-concentration experimental exposures that may not represent human vaping behaviour.

They also added an evidence map showing the different emphases of the human and animal literature.

The authors did not create a separately titled “Translational Interpretation” section as originally suggested, but the substance of the recommendation has been addressed adequately within the Discussion.

5. Overstatement of conclusions — Fully addressed.

The revised Conclusion is considerably more appropriately qualified. It now states that evidence regarding the independent harms of vaping in pregnancy remains inconclusive, identifies the animal evidence as biologically plausible but hypothesis-generating, and emphasises that heterogeneous study designs, exposure assessment and residual confounding prevent conclusions regarding relative safety.

This is much better balanced than the earlier version and avoids implying that animal findings establish causal human effects.

6. Maternal outcomes — Substantially addressed.

The manuscript now highlights the striking lack of maternal physiological outcomes in the human evidence base and more clearly connects this gap with vascular, metabolic and placental mechanisms arising from animal studies. The authors also recommend future assessment of maternal physiology alongside fetal and neonatal outcomes.

This satisfactorily addresses the original concern.

Reviewer #3: This study addresses an important and clinically relevant question regarding the role of vaping as a potential smoking-cessation and harm-reduction strategy. Given the growing use of electronic cigarettes and ongoing uncertainty regarding their comparative effectiveness, safety, and clinical applicability, the evidence synthesized in this scoping review is of considerable value to clinicians, researchers, and policymakers.

A few minor issues should nevertheless be considered before publication. First, the limited evidence directly comparing vaping with nicotine replacement therapy (NRT), an established smoking-cessation intervention in several health systems, should be highlighted more explicitly as a key evidence gap because it limits the clinical interpretation of vaping as a harm-reduction option. Second, the rationale for screening the first 100 Google Scholar results should be briefly justified in relation to JBI guidance, with the possibility of missed grey literature, such as conference abstracts and regulatory reports, acknowledged as a limitation. Third, terminology describing the S4 Appendix appraisal should remain clearly distinguished from formal risk-of-bias or quality assessment to avoid misinterpretation. Fourth, the exploratory finding regarding menthol vaping and fetal death (OR 3.27) may warrant a brief cautionary statement in the Abstract, given its magnitude and the potential for selective interpretation. Finally, the clean and tracked-changes versions should be checked to ensure complete consistency in formatting, headings, citation style, and S1–S4 Appendix numbering.

**********

what does this mean?). If published, this will include your full peer review and any attached files.

If you choose “no”, your identity will remain anonymous but your review may still be made public.

**Do you want your identity to be public for this peer review?**  For information about this choice, including consent withdrawal, please see our Privacy Policy

Reviewer #1: **Yes:**  JOHN KWAME AGBENYEAVU

Reviewer #3: No

**********

[NOTE: If reviewer comments were submitted as an attachment file, they will be attached to this email and accessible via the submission site. Please log into your account, locate the manuscript record, and check for the action link "View Attachments". If this link does not appear, there are no attachment files.]

To ensure your figures meet our technical requirements, please review our figure guidelines: https://journals.plos.org/plosone/s/figures

You may also use PLOS’s free figure tool, NAAS, to help you prepare publication quality figures: https://journals.plos.org/plosone/s/figures#loc-tools-for-figure-preparation.

NAAS will assess whether your figures meet our technical requirements by comparing each figure against our figure specifications.

# Author comment: Author response to Decision Letter 2

**DOI:** 10.1371/journal.pone.0356305.r004

24 Aug 2026

Reviewer #1: Assessment of the previous major comments

1. Critical appraisal/interpretability of the human evidence — Addressed, with a small methodological clarification desirable.

The original concern was that the review made interpretive conclusions without allowing readers to judge differences in study reliability. The authors have now introduced a structured interpretability appraisal covering study design, exposure timing, definitions of exclusive vaping/dual use/non-use, biochemical validation, previous smoking, residual confounding, confounder adjustment and overall interpretability. They appropriately clarify that this is not intended to constitute a formal risk-of-bias score.

The Results also now explicitly direct readers to S4 Appendix and describe what the appraisal contains.

I would therefore regard the original concern as substantially addressed. One minor issue remains: the manuscript states that studies were classified as higher, moderate, moderate-to-lower, or lower interpretability, but the methodology presented in the manuscript does not clearly explain the decision rules used to assign these categories. A brief explanation of how these overall ratings were determined would improve reproducibility and avoid the appearance of subjective grading.

We thank the reviewer for this helpful clarification. We agree that the basis for assigning the overall interpretability categories should be described more explicitly to improve transparency and reproducibility. We have therefore expanded the Materials and methods section to describe the decision-making process used for the structured interpretability appraisal. The revised text now specifies that ratings considered study design and data source, prospective versus retrospective exposure ascertainment, timing and frequency of exposure assessment, exposure-group definitions, biochemical validation, previous smoking and switching behaviour, sample size, confounder adjustment and potential residual confounding.

We have also clarified how these domains informed the overall categories. Higher interpretability reflected stronger methodological features across these domains, while ratings were reduced where several limitations were present, including retrospective self-reported exposure, absence of biochemical validation, small vaping groups, incomplete characterisation of previous smoking or dual use, limited confounder adjustment or substantial residual confounding. We further clarify that categories were assigned holistically rather than using a weighted numerical score and were not intended to constitute a formal risk-of-bias or quality assessment.

Location in revised manuscript: Materials and methods p. 10 and S4 Appendix.

Reviewer #3:

This study addresses an important and clinically relevant question regarding the role of vaping as a potential smoking-cessation and harm-reduction strategy. Given the growing use of electronic cigarettes and ongoing uncertainty regarding their comparative effectiveness, safety, and clinical applicability, the evidence synthesized in this scoping review is of considerable value to clinicians, researchers, and policymakers.

A few minor issues should nevertheless be considered before publication. First, the limited evidence directly comparing vaping with nicotine replacement therapy (NRT), an established smoking-cessation intervention in several health systems, should be highlighted more explicitly as a key evidence gap because it limits the clinical interpretation of vaping as a harm-reduction option. Second, the rationale for screening the first 100 Google Scholar results should be briefly justified in relation to JBI guidance, with the possibility of missed grey literature, such as conference abstracts and regulatory reports, acknowledged as a limitation. Third, terminology describing the S4 Appendix appraisal should remain clearly distinguished from formal risk-of-bias or quality assessment to avoid misinterpretation. Fourth, the exploratory finding regarding menthol vaping and fetal death (OR 3.27) may warrant a brief cautionary statement in the Abstract, given its magnitude and the potential for selective interpretation. Finally, the clean and tracked-changes versions should be checked to ensure complete consistency in formatting, headings, citation style, and S1–S4 Appendix numbering.

We thank the reviewer for their positive assessment of the manuscript and for these helpful suggestions. We have addressed each of the minor comments as detailed below.

1. Evidence comparing vaping with NRT:

We thank the reviewer for this helpful suggestion. We have revised the Discussion to highlight more explicitly the limited evidence directly comparing vaping with NRT as a key evidence gap, and to clarify that this limits conclusions regarding the relative safety and clinical role of vaping compared with established smoking-cessation strategies in pregnancy.

Location in revised manuscript: Discussion p. 32.

2. Rationale for screening the first 100 Google Scholar results:

We have clarified in the Methods section that screening the first 100 Google Scholar results was undertaken as a pragmatic supplementary search informed by JBI guidance recommending review of the first 5–10 pages of supplementary Google search results. We have also added to the limitations that restricting screening to the first 100 results may have resulted in relevant grey literature, including conference abstracts or regulatory reports, being missed.

Location in revised manuscript: Materials and methods p. 8, Limitations p. 34.

3. Terminology describing the S4 Appendix appraisal:

We thank the reviewer for this helpful comment. We have ensured that the terminology used throughout the manuscript and S4 Appendix consistently describes this as a structured interpretability appraisal, and we have explicitly clarified in the Methods that it was not intended to constitute a formal risk-of-bias or quality assessment.

Location in revised manuscript: Materials and methods p. 10 and S4 Appendix.

4. The exploratory finding regarding menthol vaping and fetal death:

We thank the reviewer for this comment. The exploratory association between mint/menthol-flavoured vaping and fetal death is not reported in the Abstract, which presents the overall findings of the scoping review rather than individual exploratory associations. We have therefore not introduced this finding into the Abstract, as doing so could give disproportionate prominence to a small subgroup analysis. In the Discussion, where the finding is reported, we have retained explicit cautionary language noting the small subgroup size, limited adjustment for confounding, wide confidence intervals, and the need for cautious interpretation.

Location in revised manuscript: Discussion p. 32.

5. Clean and tracked-changes versions: consistency in formatting, headings, citation style, and S1–S4 Appendix numbering:

We thank the reviewer for this comment. We have checked the clean and tracked-changes versions to ensure consistency in formatting, headings, citation style, and S1–S4 Appendix numbering and corrected minor inconsistencies where identified.

Attachment Submitted filename: PLOS one response to reviewer comments 2_AH.docx

# Editor report: Decision Letter 2

**DOI:** 10.1371/journal.pone.0356305.r005

**Authors:** Eman Allam

8 Sep 2026

Vaping During Pregnancy and Maternal, Fetal, and Infant Health Outcomes: A Scoping Review of Human and Animal Studies

PONE-D-26-18186R2

Dear Dr. Fielder,

We’re pleased to inform you that your manuscript has been judged scientifically suitable for publication and will be formally accepted for publication once it meets all outstanding technical requirements.

Within one week, you’ll receive an e-mail detailing the required amendments. When these have been addressed, you’ll receive a formal acceptance letter and your manuscript will be scheduled for publication.

An invoice will be generated when your article is formally accepted. Please note, if your institution has a publishing partnership with PLOS and your article meets the relevant criteria, all or part of your publication costs will be covered. Please make sure your user information is up-to-date by logging into Editorial Manager at Editorial Manager® and clicking the ‘Update My Information' link at the top of the page. For questions related to billing, please contact billing support.

If your institution or institutions have a press office, please notify them about your upcoming paper to help maximize its impact. If they’ll be preparing press materials, please inform our press team as soon as possible -- no later than 48 hours after receiving the formal acceptance. Your manuscript will remain under strict press embargo until 2 pm Eastern Time on the date of publication. For more information, please contact onepress@plos.org.

Kind regards,

Eman Allam, PhD

Academic Editor

PLOS One

Additional Editor Comments (optional):

Reviewers' comments:

# Editor report: Acceptance letter

**DOI:** 10.1371/journal.pone.0356305.r006

**Authors:** Eman Allam

PONE-D-26-18186R2

PLOS One

Dear Dr. Fielder,

I'm pleased to inform you that your manuscript has been deemed suitable for publication in PLOS One. Congratulations! Your manuscript is now being handed over to our production team.

At this stage, our production department will prepare your paper for publication. This includes ensuring the following:

* All references, tables, and figures are properly cited

* All relevant supporting information is included in the manuscript submission,

* There are no issues that prevent the paper from being properly typeset

You will receive further instructions from the production team, including instructions on how to review your proof when it is ready. Please keep in mind that we are working through a large volume of accepted articles, so please give us a few days to review your paper and let you know the next and final steps.

Lastly, if your institution or institutions have a press office, please let them know about your upcoming paper now to help maximize its impact. If they'll be preparing press materials, please inform our press team within the next 48 hours. Your manuscript will remain under strict press embargo until 2 pm Eastern Time on the date of publication. For more information, please contact onepress@plos.org.

You will receive an invoice from PLOS for your publication fee after your manuscript has reached the completed accept phase. If you receive an email requesting payment before acceptance or for any other service, this may be a phishing scheme. Learn how to identify phishing emails and protect your accounts at https://explore.plos.org/phishing.

If we can help with anything else, please email us at customercare@plos.org.

Thank you for submitting your work to PLOS One and supporting open access.

Kind regards,

PLOS One Editorial Office Staff

on behalf of

Dr. Eman Allam

Academic Editor

PLOS One
